# Simulation and experimental research on trans-media vehicle water-entry motion characteristics at low speed

**DOI:** 10.1371/journal.pone.0178461

**Published:** 2017-05-30

**Authors:** Jian Yang, Yongli Li, Jinfu Feng, Junhua Hu, An Liu

**Affiliations:** 1 School of Aeronautics and Astronautics Engineering, Air Force Engineering University, Xi’an, Shaanxi, China; 2 Engineering University of CAPF, Xi’an, Shaanxi, China; Beihang University, CHINA

## Abstract

The motion characteristics of trans-media vehicles during the water-entry process were explored in this study in an effort to obtain the optimal water-entry condition of the vehicle for developing a novel, single control strategy integrating underwater non-control and in-air control. A water-entry dynamics model is established by combining the water-entry motion characteristics of the vehicle in uncontrolled conditions at low speed with time-varying parameters (e.g. buoyancy, added mass). A water-entry experiment is designed to confirm the effectiveness of the established model. After that, by comparing the experimental results with the simulated results, the model is further modified to more accurately reflect water-entry motion. The change laws of the vehicle’s attitude and position during the water-entry process are also obtained by analyzing the simulation of the modified model under different velocity, angle, and angle of attack conditions. The results presented here have guiding significance for the future realization of reaching the stable underwater navigation state of the vehicle after water-entry process.

## Introduction

Recent advancements in aviation and navigation technology have resulted in the extensive theoretical development of the trans-media vehicle; as suggested by its name, this type of vehicle can traverse the water-air interface underwater and air navigation requirements and achieve long-term alternate navigation between the two media [1,2]. The trans-media vehicle has already been the subject of substantial research interest [3]. Unlike the air-dropped torpedo, submarine-launched missile, or other single-medium crossing vehicles, the trans-media vehicle has the capability of crossing the water-air interface repeatedly. The full mission profile of the vehicle includes four stages: Air flight, water-entry crossing, underwater navigation, and water-exit crossing. In the stages of media crossing, many complex phenomena that caused by the great difference of characteristic between two kinds of medium have adverse effects on the stability of the vehicle attitude; this condition makes successful crossing between media extremely challenging [4]. Thus, designing a control system corresponding to the crossing process is a popular research objective. Due to the very brief time period during which media crossing takes place, However, designing a suitable system that effectively controls the attitude of the vehicle during crossing is difficult because of the brief time period during which media crossing occurs. A novel strategy for controlling the water-entry process of trans-media vehicle is proposed in this study. The strategy involves underwater non-control and in-air control during the water-entry crossing process. By studying the motion characteristics of the vehicle under different initial water-entry conditions, the optimal conditions for maintaining attitude stability and stable underwater navigation can be obtained. Then, the corresponding air-control system will be designed to ensure the vehicle reaches the correct conditions to realize desired state. In this paper, the motion characteristics of vehicle in water-entry process under the different initial conditions of the vehicle are studied.

For trans-media vehicles, the water-entry process is characterized by integrity and typicality as the air trajectory continues and the underwater trajectory begins. The problem of unsteady motion and interaction among air, water, and elastic objects is very complex [5]. It is a fluid dynamics problem involving a free surface and special cavitations, is a high speed impact problem that may cause the potential structural damage, and is a dynamics problem of elastic object motion. The study of the water-entry problem was originally from the beginning of the 20th century. At this time, the main research method was to use the means of experiment. Among them, Worthington [6] used a flash camera to carry out a large number of experimental observations on the flow phenomenon when sphere and other object enter the water vertically. Since then, the importance of water-entry research has gradually been recognized by the public after the 1950s. Therefore, many research institutions, colleges and universities carried out a large number of basic researches on the issue of water-entry. The main research contents included the flow phenomena about vertical and inclined water-entry, the development process of cavity, the dynamic variation law of the hydrodynamics for motion body and the stability of water-entry trajectory. The most representative studies are the experimental research about the phenomenon of whip, the changing process of water-entry cavity and its influencing factors which carried out by Waugh [7], Birkhoff [8] and May [9]. In recent years, with the rapid development of experimental equipment, experimental methods and experimental technology, the study of water-entry problem has more effective methods and ways. Therefore, the understanding of the water-entry problem has gradually become in-depth. Truscott [10,11] discussed the current progress of the research on the water-entry problem, carried out a large number of experimental research for the water-entry problem of the sphere and the cylinder, analyzed the influence of the coating and water-entry velocity on the formation, development and closure of cavity in detail, and obtained the flow characteristics of the cavity when the motion body enter the water with the conditions of different rotating angular velocity. Chuntao He [12] carried out a water-entry experiment of the cylinder at low-speed, analyzed the flow phenomenon when the cylinder vertically, aslant, serially and paratactically enter the water. And the relationship between the water-entry velocity and the way of cavity closure is investigated. So did the influence between the cavities when the body serially and paratactically enters the water. Fasuo Ya [13] and Alaoxi [14] respectively used the experiment to carefully observe impact load and flow field of the ball and conical body at the preliminary stage of water-entry, and summarized the changes of the flow field and the temporal and spatial distribution of the pressure at the preliminary stage of water-entry.

However, the higher difficulty and cost of the experiment and advanced computer technologies have facilitated an increasing number of numerical simulation methods (NSM) that are applicable for investigating the water-entry movement of vehicles. At present, the numerical calculation research on the water-entry problem is mainly focused on two directions: one is the research of the flow and load at the stage of water-entry smacking; the other is the whole flow process of the water-entry cavity flow field. In the first research direction, Geers [15] firstly proposed the boundary element method to study the water-entry impact of a revolving structure. Then Korobin [16]combined the finite element method with the boundary element method to study water-entry impact. ParkMansung [17] utilized the panel method to numerically calculate the impact load of the tangent arch at any water-entry angle, and carried on the numerical simulation analysis to the whip behavior of the arch. Hanbing Luo [18] explored the water-entry slamming of a two-dimensional rigid wedge based on the explicit finite element method and Lagrange algorithm. The research about numerical prediction was carried and the numerically simulated results were verified through experimentation. In the second research direction, J.M.Gordillo [19] used the potential flow theory to numerically simulate the variation of the cavity morphology with time under the high Reynolds number, and obtained the relationship between the shape of the cavity closure point, the cavity minimum radius and the closure time; Stephan Gekle [20] used the combination of numerical and experimental methods to establish the mathematical model of the high-speed jet-flow formation process when the disk vertically enter the water in uniform speed and the water-entry cavity is closed, and analyzed the cavity closure process and the jet-flow formation process by numerical simulation. The numerical simulation results are in good agreement with the experimental results. Qingpeng Ma [21] carried out the numerical simulation of the vertical high-speed water-entry problem for cone-shaped projectiles with different taper angles and obtained the influence of the projectile cone angle on the cavity formation and fluid dynamics.

Extant research on the water-entry dynamics is generally concerned with particular working conditions or individual aspects of the process. Meanwhile, comprehensive theoretical research on the trajectory characteristics of the water-entry process as a whole is scarce. According to that problem, in this study, under the assumption that the vehicle is moving at low speed, and that the effect of the factors such as the free surface and cavity are ignored, by the theoretical analysis, a water-entry dynamics model of the trans-media vehicle is established. Then, a water-entry experiment with varying initial vehicle conditions is utilized to verify the effectiveness and feasibility of the model. The model is slightly modified on the basis of the comparison between the experimental and simulated results. Then the motion law and trajectory of the vehicle in the water-entry process is investigated under the improved model.

## Dynamic water-entry model

### Force analysis of the vehicle in water-entry process

**Fig 1:**
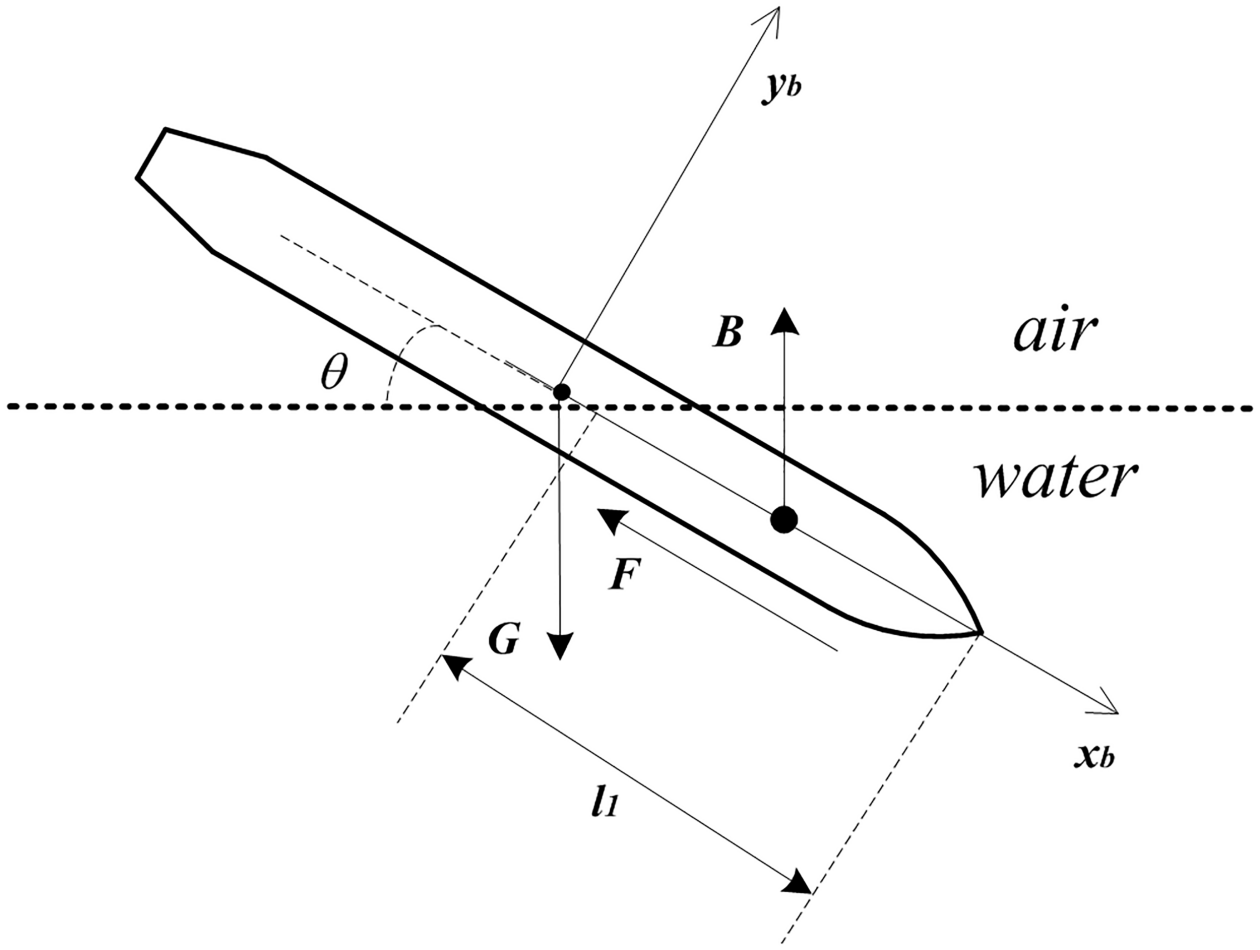
Force analysis in the water-entry process of the vehicle.

As shown in Fig 1, the O-*x*_*b*_*y*_*b*_*z*_*b*_ is the reference frame. At the same time, it is selected in this study that the *x*_*b*_ axis of the body coordinate system is forward along the vertical axis of the vehicle, *y*_*b*_ axis is in the longitudinal symmetry plane of the vehicle and perpendicular to the *x*_*b*_ axis, *z*_*b*_ axis is perpendicular to the body longitudinal symmetry plane and points to the right side of the vehicle body. The origin of the reference frame is the barycenter of vehicle. Air force is neglected, and the vehicle is affected by G, B and F. G represents for the gravity, which is a constant value. B represents for the buoyancy and F represents for the fluid force. In the water-entry process, the two kinds of force vary with the length of the underwater part of the vehicle which is represented by *l*_*1*_ in the paper.

The vehicle parameters and crossing motion model are built under the following basic assumptions and omissions:

1. The vehicle is a rigid body and is symmetrical about the x-y plane;
2. The mass distribution is uniform, and the barycenter is located at the longitudinal axis of the vehicle;
3. The fluid is infinite, and the vehicle is stationary before it crosses the media;
4. Regardless of the rotation of the earth or the curvature of the ball, the ground coordinate system is regarded as the inertial coordinate system;
5. The submerged volume of the vehicle is not affected by the free surface.
6. According to the conclusion in [22], the formation and collapse of cavity in the water-entry process of the structural body at low speed conditions have little effect on the motion characteristics. Therefore, the effect of cavity is neglected in the model in this study.

In this study, only the motion conditions of the vehicle in the longitudinal plane are considered. The initial water-entry kinetic model is obtained according to the force analysis:

$$\{\begin{array}{l} G_{x}+B_{x}+F_{x}=m(dv_{x}/dt-v_{y}\omega_{z})\\ G_{y}+B_{y}+F_{y}=m(dv_{y}/dt+v_{x}\omega_{z})\\ M_{z}=J_{z}\cdot d\omega_{z}/dt \end{array}$$ (1)

where the subscripts x and y denote the component of corresponding force in x axis and y axis in the body coordinates. *M*_*z*_ represents for the rotating moment of the vehicle around the z axis; *m* represents for the mass of the vehicle; *J*_*z*_ represents for the rotary inertia of the vehicle to the z axis of the body coordinates; *v* represents for the velocity of the vehicle; *a* represents for the acceleration of the vehicle; *ω*_*z*_ represents for the angular velocity around *z* axis.

### Relevant parameters of the vehicle

#### Configuration parameters of the vehicle

**Fig 2:**
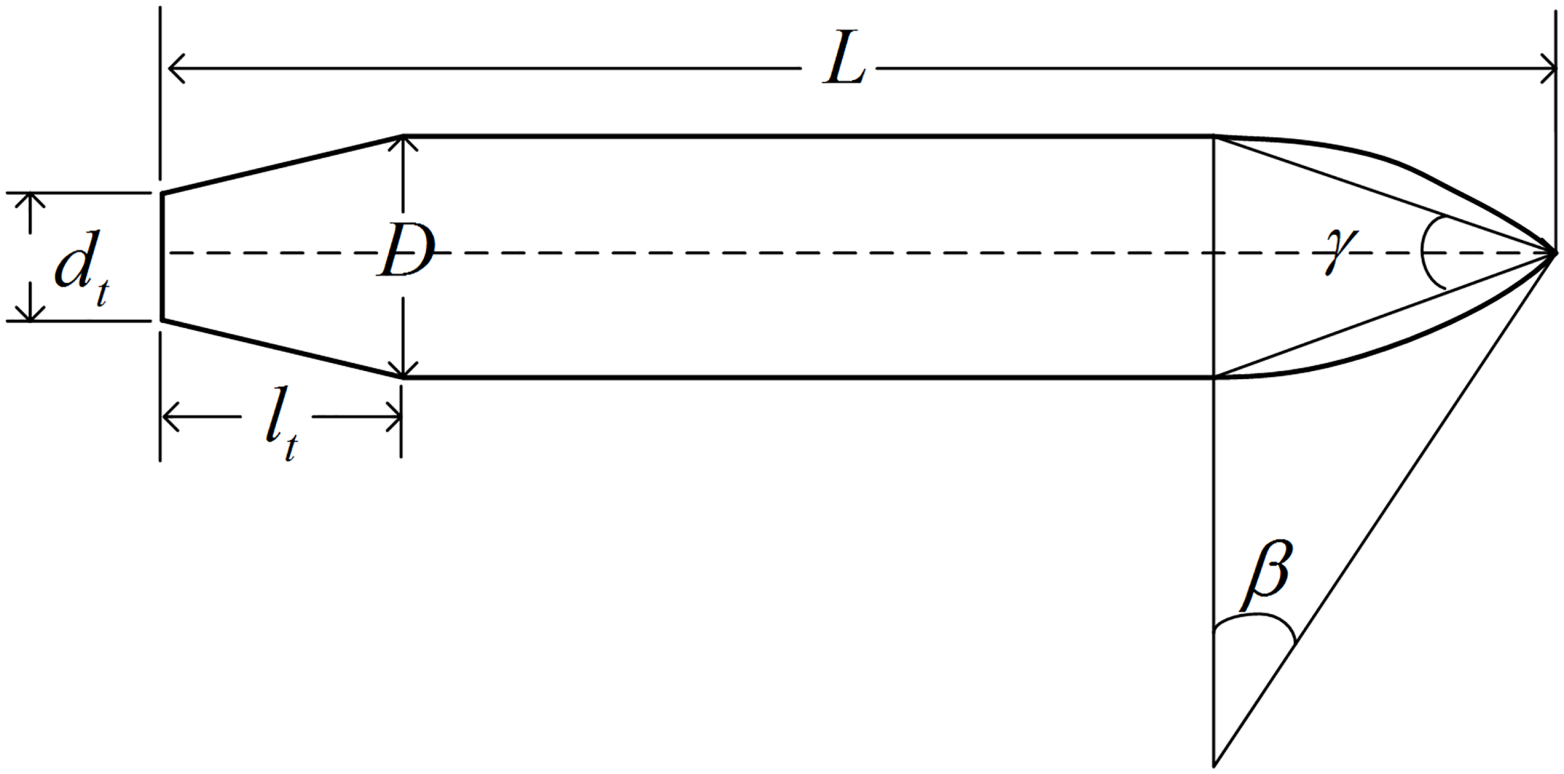
Contour of the vehicle (unit: m).

This study considers a vehicle composed of a rotating cylinder with a peaked-arch-shaped head and a linear-cutting-shaped tail. The mass distribution of the vehicle is uniform and the length *L* of the vehicle is 5.33m. The physics model and corresponding parameters of the vehicle are shown in Fig 2.

Apex angle *γ* = 30°, sweep angle *β* = 30°, tail length *l*_t_ = 0.6m, minimum tail diameter *d*_t_ = 0.2665m, maximum body diameter *D* = 0.533m, and scanning radius *r* = 1.9895m. The radius of any position of the body of the vehicle can be easily obtained according to these parameters.

#### Fluid force F

In the water-entry process, the fluid force is continuously distributed on the surface of the vehicle with some laws. The distribution laws are decided by the factors such as the motion states of the vehicle and the characteristics of the fluid. At present, the fluid force that the vehicle is subjected in the actual fluid movement is difficult to obtain directly. In order to simply the problem, by the theoretical calculation, and based on the suitable assumption, the fluid force can be separated as two kinds of force to be obtained. One force is in the ideal state which has no viscousness, and the other force is the fluid force with the viscousness [23].

1) Ideal fluid

For the ideal fluid force, according to the [24], in the water-entry process, the moment of momentum *K*_*f*_ and the momentum *Q*_*f*_ are as follow.

$$\{\begin{array}{c} Q_{f}=-\int_{\Omega }\rho \sigma nd\Omega \\ K_{f}=-\int_{\Omega }\rho (r\times n)\sigma nd\Omega \end{array}$$ (2)

where, *σ* is the velocity potential function of the field; *ρ* is density of the fluid; *r* is the radius vector at any point in the flow field; *n* is the unit outside normal vector on the wetting surface of the vehicle that is expressed as Ω. The object plane boundary condition of the ideal fluid can be expressed as:

$${\frac{\partial \sigma }{\partial n}|}_{\Omega }=v_{Ox}n_{x}+v_{Oy}n_{y}+v_{Oz}n_{z}+(z\omega_{y}-y\omega_{z})n_{x}+(x\omega_{z}-z\omega_{x})n_{y}+(y\omega_{x}-x\omega_{y})n_{z}$$ (3)

where, *v*_*Ox*_, *v*_*Ov*_, *v*_*Oz*_ are respectively the components of the velocity at the origin O of the body coordinate system on the three axes; *ω*_*x*_, *ω*_*v*_, *ω*_*z*_ are respectively the components of the angular velocity on the three axes. *x*, *y*, *z* are respectively the components of the vector *r* on the three axes. *n*_*x*_, *n*_*v*_, *n*_*z*_ are respectively the components of the unit outside normal vector on the three axes.

Using the Kirchhoff method and according to the superposition principle of potential flow, the velocity potential function of the ideal fluid can be expressed as:

$$\begin{array}{ll} \sigma (x,y,z,t) & =v_{Ox}(t)\sigma_{1}(x,y,z)+v_{Oy}(t)\sigma_{2}(x,y,z)+v_{Oz}(t)\sigma_{3}(x,y,z)+\\ & =\omega_{x}(t)\sigma_{4}(x,y,z)+\omega_{y}(t)\sigma_{5}(x,y,z)+\omega_{z}(t)\sigma_{6}(x,y,z) \end{array}$$ (4)

where, *σ*_1_, *σ*_2_, *σ*_3_ are respectively the unit velocity potential function of the translational motion in the direction of the three axes of the body coordinate system; *σ*_4_, *σ*_5_, *σ*_6_ are respectively the unit velocity potential function of the rotation motion movement in the direction of the three axes. Taking Eq (5) into Eq (2), it is obtained as follow:

$$\{\begin{array}{c} \frac{\partial \sigma_{1}}{\partial n}=n_{x},\frac{\partial \sigma_{2}}{\partial n}=n_{y},\frac{\partial \sigma_{3}}{\partial n}=n_{z}\\ \frac{\partial \sigma_{4}}{\partial n}=yn_{z}-zn_{y},\frac{\partial \sigma_{5}}{\partial n}=zn_{x}-xn_{z},\frac{\partial \sigma_{6}}{\partial n}=xn_{y}-yn_{x} \end{array}$$ (5)

Make the following settings:

$$\lambda_{ij}=-\int_{\Omega }\rho \sigma_{j}\frac{\partial \sigma_{i}}{\partial n}d\Omega ,\;i=1,2,\cdots ,6;j=1,2,\cdots ,6$$ (6)

So *K*_*f*_ and *Q*_*f*_ can be obtained as follow:

$$\left[\begin{array}{c} Q_{fx}\\ Q_{fy}\\ Q_{fz}\\ K_{fx}\\ K_{fy}\\ K_{fz} \end{array}\right]=\lambda \left[\begin{array}{c} v_{x}\\ v_{y}\\ v_{z}\\ \omega_{x}\\ \omega_{y}\\ \omega_{z} \end{array}\right],\;\lambda =\left[\begin{array}{cccccc} \lambda_{11} & \lambda_{12} & \lambda_{13} & \lambda_{14} & \lambda_{15} & \lambda_{16}\\ \lambda_{21} & \lambda_{22} & \lambda_{23} & \lambda_{24} & \lambda_{25} & \lambda_{26}\\ \lambda_{31} & \lambda_{32} & \lambda_{33} & \lambda_{34} & \lambda_{35} & \lambda_{36}\\ \lambda_{41} & \lambda_{42} & \lambda_{43} & \lambda_{44} & \lambda_{45} & \lambda_{46}\\ \lambda_{51} & \lambda_{52} & \lambda_{53} & \lambda_{54} & \lambda_{55} & \lambda_{56}\\ \lambda_{61} & \lambda_{62} & \lambda_{63} & \lambda_{64} & \lambda_{65} & \lambda_{66} \end{array}\right]$$ (7)

where, *v* is the velocity of the origin of the body coordinate system; *ω* is the rotating angular velocity of the vehicle; the subscripts *x*, *y* and *z* represent the components on the corresponding coordinate axes; *λ*_*ij*_ (*i* = 1, 2, 3, 4, 5, 6; *j* = 1, 2, 3, 4, 5, 6) is the added mass. And then the force of ideal fluid on the vehicle is obtained by Eq (8):

$$\left[\begin{array}{c} F_{ix}\\ F_{iy}\\ F_{iz}\\ M_{ix}\\ M_{iy}\\ M_{iz} \end{array}\right]=-\lambda \left[\begin{array}{c} \text{d}v_{x}/\text{d}t\\ \text{d}v_{y}/\text{d}t\\ \text{d}v_{z}/\text{d}t\\ \text{d}\omega_{x}/\text{d}t\\ \text{d}\omega_{y}/\text{d}t\\ \text{d}\omega_{z}/\text{d}t \end{array}\right]-\left[\begin{array}{cccccc} 0 & -\omega_{z} & \omega_{y} & 0 & 0 & 0\\ \omega_{z} & 0 & -\omega_{x} & 0 & 0 & 0\\ -\omega_{y} & \omega_{x} & 0 & 0 & 0 & 0\\ 0 & 0 & 0 & 0 & -\omega_{z} & \omega_{y}\\ 0 & 0 & 0 & \omega_{z} & 0 & -\omega_{x}\\ 0 & 0 & 0 & -\omega_{y} & \omega_{x} & 0 \end{array}\right]\left[\begin{array}{c} Q_{fx}\\ Q_{fy}\\ Q_{fz}\\ K_{fx}\\ K_{fy}\\ K_{fz} \end{array}\right]-\left[\begin{array}{cccccc} 0 & 0 & 0 & 0 & 0 & 0\\ 0 & 0 & 0 & 0 & 0 & 0\\ 0 & 0 & 0 & 0 & 0 & 0\\ 0 & -v_{z} & v_{y} & 0 & 0 & 0\\ v_{z} & 0 & -v_{x} & 0 & 0 & 0\\ -v_{y} & v_{x} & 0 & 0 & 0 & 0 \end{array}\right]\left[\begin{array}{c} Q_{fx}\\ Q_{fy}\\ Q_{fz}\\ K_{fx}\\ K_{fy}\\ K_{fz} \end{array}\right]$$ (8)

In addition, according to the structural characteristics of the vehicle, the added mass matrix can be changed to the following matrix [24]:

$$\lambda =\left[\begin{array}{cccccc} \lambda_{11} & 0 & 0 & 0 & 0 & 0\\ 0 & \lambda_{22} & 0 & 0 & 0 & \lambda_{26}\\ 0 & 0 & \lambda_{33} & 0 & \lambda_{35} & 0\\ 0 & 0 & 0 & \lambda_{44} & 0 & 0\\ 0 & 0 & \lambda_{53} & 0 & \lambda_{55} & 0\\ 0 & \lambda_{62} & 0 & 0 & 0 & \lambda_{66} \end{array}\right]$$ (9)

Taking Eqs (9) and (7) into Eq (8), considering that the study only focuses on the characteristics of the longitudinal motion, the fluid force of the ideal fluid acting on the vehicle is obtained.

$$\{\begin{array}{lll} F_{ix} & = & -\lambda_{11}{\dot{v}}_{x}\text{+}\omega_{z}\left(\lambda_{22}v_{y}+\lambda_{26}\omega_{z}\right)\\ F_{iy} & = & -\lambda_{22}{\dot{v}}_{y}-\lambda_{26}{\dot{\omega }}_{z}{}_{z}-\omega_{z}\lambda_{11}v_{x}\\ M_{iz} & = & -\lambda_{62}{\dot{v}}_{y}-\lambda_{66}{\dot{\omega }}_{z}+v_{y}\lambda_{11}v_{x}-v_{x}\left(\lambda_{22}v_{y}+\lambda_{26}\omega_{z}\right) \end{array}$$ (10)

According to the corresponding theorem of the slender body, the corresponding added mass in Eq (10) can be obtained by Eq (11).

$$\left\{ \begin{array}{l} \lambda_{22}=\pi \rho_{0}\int_{0}^{x_{a}}R^{2}\left(x\right)dx\\ \lambda_{26}=\lambda_{62}=\pi \rho_{0}\int_{0}^{x_{a}}R^{2}\left(x\right)xdx\\ \lambda_{66}=\pi \rho_{0}\int_{0}^{x_{a}}R^{2}\left(x\right)x^{2}dx \end{array}\right.$$ (11)

Because the value of *λ*_11_ is very small, this study treats it as zero. And unlike a single underwater vehicle, the added mass of the trans-media changes after it enters the water. Therefore, duo to the time-varying characteristics of the added mass, as described in detail previously [24], this study considered the its changing rate into the model. Then the Eq (10) is improved as follow:

$$\{\begin{array}{lll} F_{ix} & = & -\lambda_{11}{\dot{v}}_{x}-{\dot{\lambda }}_{11}v_{x}\text{+}\omega_{z}\left(\lambda_{22}v_{y}+\lambda_{26}\omega_{z}\right)\\ F_{iy} & = & -\lambda_{22}{\dot{v}}_{y}-{\dot{\lambda }}_{22}v_{y}-\lambda_{26}{\dot{\omega }}_{z}-{\dot{\lambda }}_{26}w_{z}-\omega_{z}\lambda_{11}v_{x}\\ M_{iz} & = & -\lambda_{62}{\dot{v}}_{y}-{\dot{\lambda }}_{62}v_{y}-\lambda_{66}{\dot{\omega }}_{z}-{\dot{\lambda }}_{66}w_{z}+v_{y}\lambda_{11}v_{x}-v_{x}\left(\lambda_{22}v_{y}+\lambda_{26}\omega_{z}\right) \end{array}$$ (12)

2) Viscous fluid force

The study is focused on the motion characteristics of the vehicle in low speed. Therefore, according to the conclusions in [25], in the process to calculate the viscous fluid force, the study only considers the effect of the velocity and the angle of attack. And it is assumed that the viscous fluid force coefficient is expressed by two independent functions multiplied, zero angle of attack dynamic coefficient and angle of attack variation functions; that is:

$$C_{x}=C_{x_{0}}\left(v\right)\cdot f_{x}\left(\alpha \right)$$ (13)

where C_*x*0_(*v*) is the viscous fluid force coefficient of the vehicle when its angle of attack *α* is zero and *f*_*x*_(*α*) is the effect of angle of attack *α* on the dynamic coefficient.

Unfold *f*_*x*_(*α*) nearby *α* = 0 via Taylor series:

$$f_{x}\left(\alpha \right)=f_{x}\left(0\right)+\sum_{n=1}^{\infty }{\left(\frac{d^{n}f_{x}}{d\alpha^{n}}\right)}_{0}\frac{\alpha^{n}}{n!}$$ (14)

Based on consideration that the magnitude of the viscous fluid force is independent of positive and negative *α* values, the viscous fluid force is an even function of *α*. Therefore, omitting the odd power in Eq (13) and taking *α* to the fourth power yields the following:

$$f_{x}\left(\alpha \right)=f_{x}\left(0\right)+{\left(\frac{d^{2}f_{x}}{d\alpha^{2}}\right)}_{0}\frac{\alpha^{2}}{2!}\text{+}{\left(\frac{d^{4}f_{x}}{d\alpha^{4}}\right)}_{0}\frac{\alpha^{4}}{4!}$$ (15)

The viscous fluid force when *α* = 0 is accounted for by Eq (15), where $S=2\pi \int_{0}^{L-l_{1}}R\left(x\right)dx$ is the submerged area of the vehicle.

$$F_{\mu x_{0}}=C_{x_{0}}\left(v\right)\cdot \frac{\rho v^{2}}{2}S$$ (16)

Therefore, *f*_*x*_ (0) = 1

Make

$${\left(\frac{d^{2}f_{x}}{d\alpha^{2}}\right)}_{0}=2k_{1}{\left(\frac{d^{4}f_{x}}{d\alpha^{4}}\right)}_{0}=24k_{2}$$ (17)

So

$$f_{x}\left(\alpha \right)=\left(1+k_{1}\alpha^{2}\text{+}k_{2}\alpha^{4}\right)$$ (18)

The viscous fluid force coefficient is calculated by Eq (18):

$$C_{x}=C_{x_{0}}\left(v\right)\cdot \left(1+k_{1}\alpha^{2}\text{+}k_{2}\alpha^{4}\right)$$ (19)

*C*_*x*0_(*v*) and the coefficient *k*_1_and *k*_2_ can be obtained via numerical calculation in CFX software. *C*_*x*0_(*v*) does not change obviously with speed when the vehicle is moving consistently at low speed. Here, *C*_*x*0_(*v*) is considered as a constant *C*_*x*0_. Thus:

$$C_{x}=C_{x_{0}}\cdot \left(1+k_{1}\alpha^{2}\text{+}k_{2}\alpha^{4}\right)$$ (20)

where, the main calculation process of parameters *k*_1_ and *k*_2_ is as follow. Firstly, many state points with different angles of attack are selected as the feature points of CFX fluid simulation. Then, after obtaining the viscous fluid dynamic coefficients of the vehicle corresponding to each selected feature point by CFD fluid simulation software, based on the idea of polynomial regression analysis, the Eq 19 is used as regression model and the obtained viscous fluid dynamic coefficients that correspond to the feature points are used as the sample data. After that by using the method of least squares estimation in the regression analysis, the parameters *k*_1_ and *k*_2_ in the model is estimated and then the estimated values of *k*_1_ and *k*_2_ are obtained.

The viscous fluid force can be obtained as follows:

$$F_{\mu }=C_{x}\cdot \frac{1}{2}\rho Sv^{2}$$ (21)

The viscous fluid position force imposed on the vehicle is:

$$\left\{ \begin{array}{l} F_{\mu x}=-F_{\mu }\cdot \text{cos}\alpha \\ F_{\mu y}=F_{\mu }\cdot \text{sin}\alpha \\ M_{\mu z}=-F_{\mu y}\cdot \frac{1}{2}l_{1} \end{array}\right.$$ (22)

where *F*_*μx*_, *F*_*μy*_, and *M*_*μz*_ are the drag, lift, and pitching moment, respectively, caused by the action of the viscous fluid.

#### Analysis of other forces of the vehicle and related parameters

For the other forces and the related parameters of the vehicle, as described in detail previously [24], B and its location of the vehicle can be calculated as follows:

$$B=\pi \rho_{0}g\int_{0}^{l_{1}}R^{2}\left(x\right)dx$$ (23)

$$x_{b0}=\frac{\pi \rho_{0}g\int_{0}^{l_{1}}R^{2}\left(x\right)xdx}{B}$$ (24)

Where *ρ*_0_ = 1.0 × 10^3^ *kg*/*m*^3^ is the density of water.

Gravity *G* remains unchanged during the whole water-entry process.

$$G=mg=\rho Vg=\rho \pi g\int_{0}^{L}R^{2}\left(x\right)dx$$ (25)

where *ρ* = 1.2 × 10^3^ *kg*/*m*^3^ is the vehicle density. The location of the center of mass is:

$$x_{0}=\frac{\int_{0}^{L}R^{2}\left(x\right)xdx}{\int_{0}^{L}R^{2}\left(x\right)dx}$$ (26)

The rotating moment around the z axis of the vehicle is the moment *M*_z_ produced by the action of the fluid. The rotary inertia of the vehicle to the z axis of the body coordinate *J*_*z*_ can be obtained via the parallel axis theorem and by calculating the rotary inertia of a disc-shaped object:

$$J_{z}=\pi \rho \int_{0}^{L}R^{2}(x)\left[1/4R^{2}(x)+{\left(x-x_{0}\right)}^{2}\right]dx$$ (27)

### Water-entry dynamic model

In summary, the water-entry process dynamic model based on Eq (1) is established below:

$$\left\{ \begin{array}{l} F_{ix}+F_{\mu x}+(B-G)\text{sin}\theta =m(dv_{x}/dt-v_{y}\omega_{z})\\ F_{iy}+F_{\mu y}+(B-G)\text{cos}\theta =m(dv_{y}/dt+v_{x}\omega_{z})\\ M_{iz}+M_{\mu z}+Bx_{b}\text{cos}\theta =J\cdot d\omega_{z}/dt \end{array}\right.$$ (28)

The equations above form the water-entry dynamic model of the vehicle at low speed, and they can be solved by given the corresponding initial value conditions.

## Model validations

The low-speed water-entry motion characteristics were investigated experimentally in order to validate the dynamic model discussed above. We built a water-entry experiment environment for the vehicle and ran experiments under the given initial conditions, then recorded and processed the experiment data to determine the actual vehicle movement during the water-entry process. We then compared the experimental and theoretical results under the same conditions to validate the accuracy of the proposed model in the paper.

### Experimental procedure

**Fig 3:**
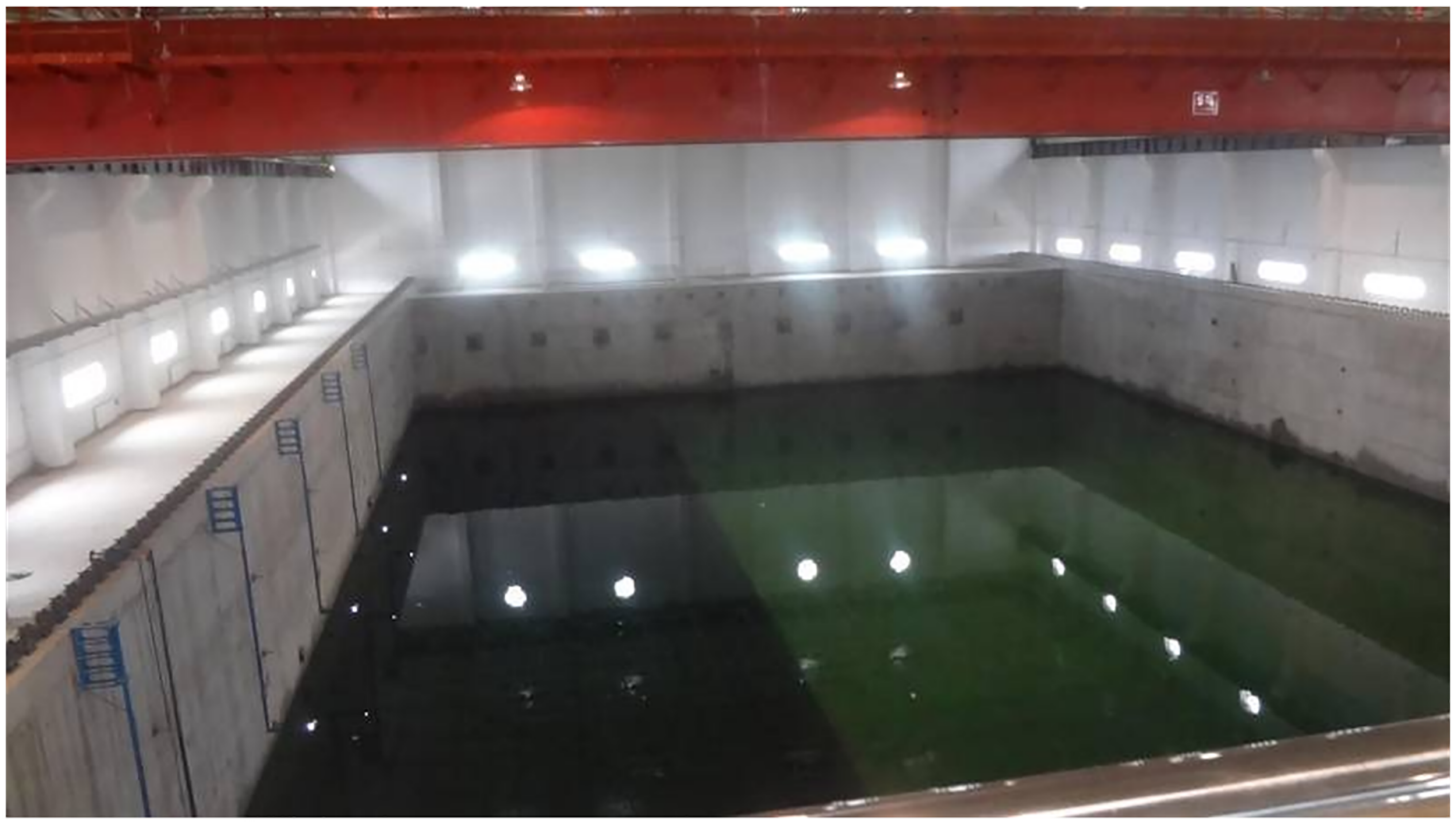
The indoor experiment pool.

The experiment was conducted in an indoor pool that is 80 m long, 30 m wide and 10 m deep. The specific pool environment is shown in Fig 3.

**Fig 4:**
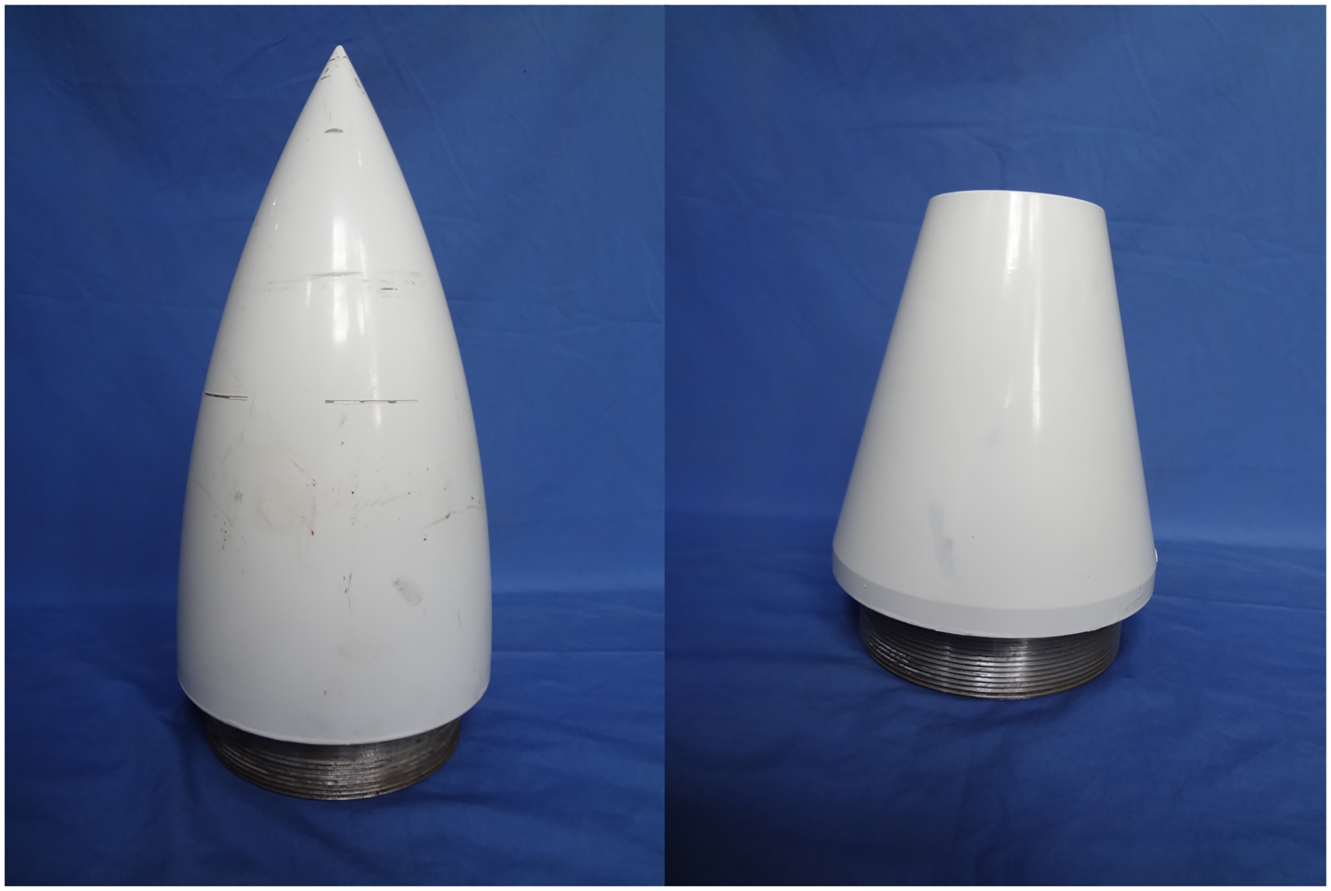
The machined head and tail.

**Fig 5:**
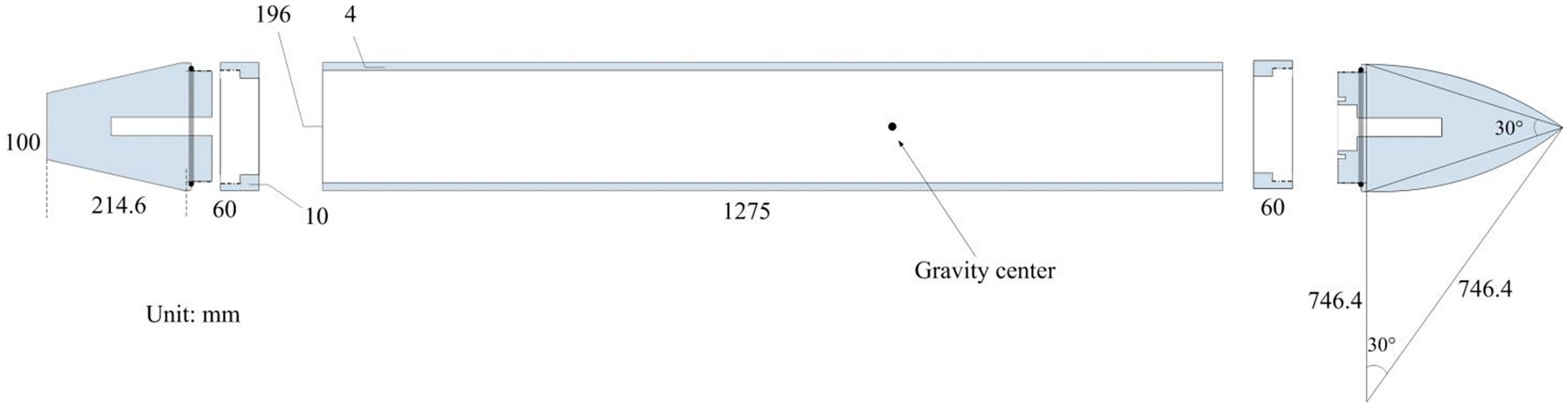
Schematic of AUV dimensions.

The device shows in Figs 4 and 5. The Fig 4 shows the machined head and tail of the experimental model. As shown in Fig 5, the experimental vehicle can be divided into three parts: Head, tail, and body. The body was machined from steel pipe, the head and tail are aluminum, and the three parts are connected by screw thread. Cylindrical and deep grooves made on the head and tail reduces the weight of the model and cylindrical grooves with a larger diameter at the rear end of the head are used to mount the acceleration sensor. The weight of the model after assembly is 64kg, volume is 0.05m^2^, average density is 1.2kg/m^2^, and the moment of inertia is 33.5kg • m^2^. The length of AUV is 2m and the center of gravity is located 1.1m from the tail(0.9m away from the head). The concrete model dimensions are shown in Fig 5.

The devices required for the experiment include the experimental observation system, model-launching system, and data acquisition system. The experimental observation system is mainly composed of an onshore high-speed camera, an underwater high-speed camera, a control operating platform, and other accessory devices. The model launching system mainly includes a high-pressure gas device, a launch tube and its support mechanism, and a mobile device. The data acquisition system mainly includes an acceleration sensor, data transmission processing software, and computer.

**Fig 6:**
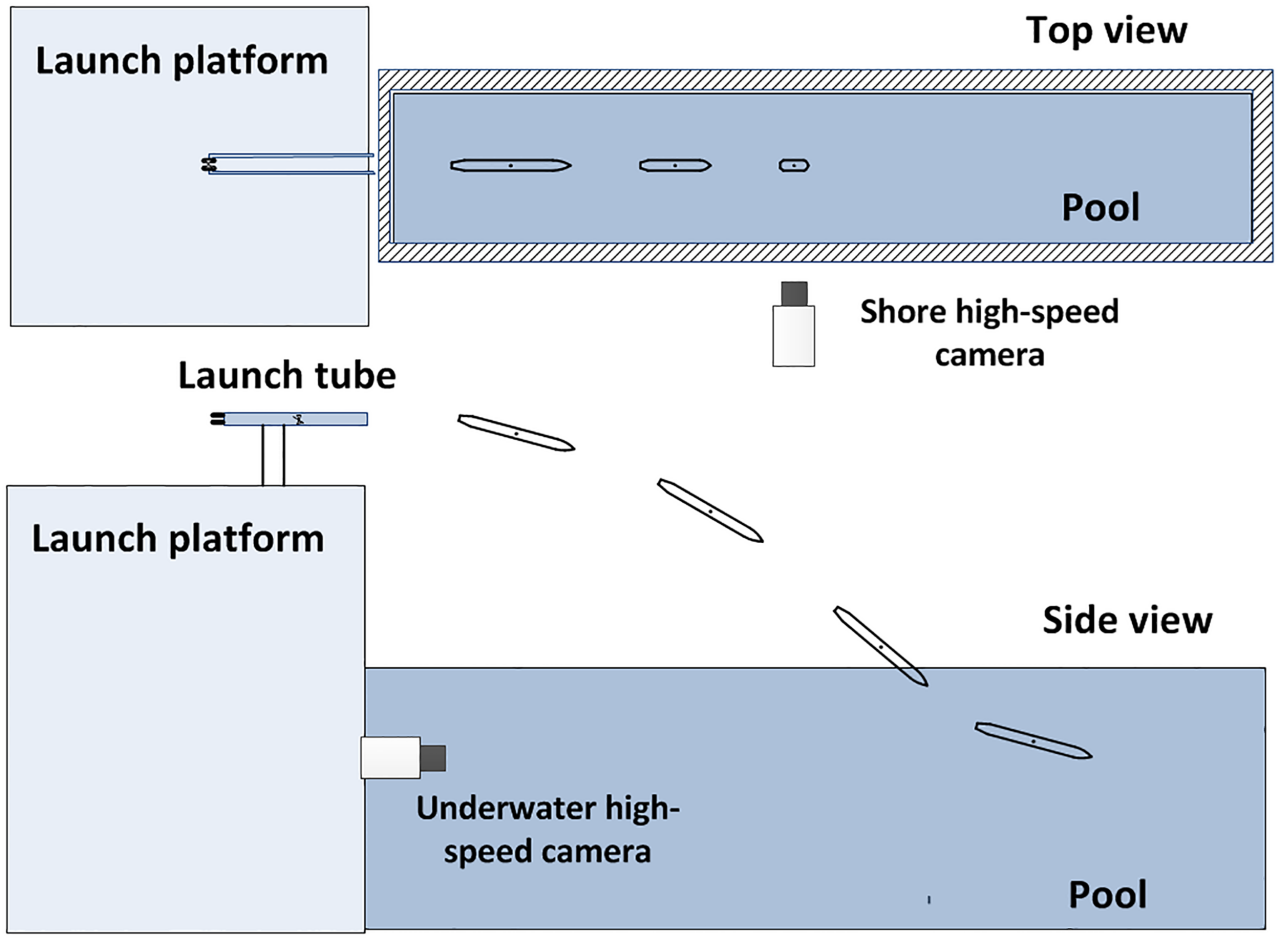
Schematic diagram of the experiment system.

As shown in Fig 6, the high-pressure launcher provided sufficient power to launch the model through the tube into the pool. Prior to the launch, the initial pressure of the air cannon in the launcher was adjusted with the air compressor and the angle of the launch tube bracket was altered as necessary to meet the requirements of the water-entry experiment for the model under different initial conditions. After the launch, an onshore high-speed camera was utilized to capture the trajectory and the attitude of the experimental model in the air and an underwater high-speed camera was utilized to capture the same data after entering the water. The acceleration sensor at the experimental model’s forepart recorded changes in acceleration throughout the process. The movement situations of the vehicle in the water-entry process under varying initial conditions were determined after the experiment by analyzing video data from the camera and numerical data from the acceleration sensor.

**Fig 7:**
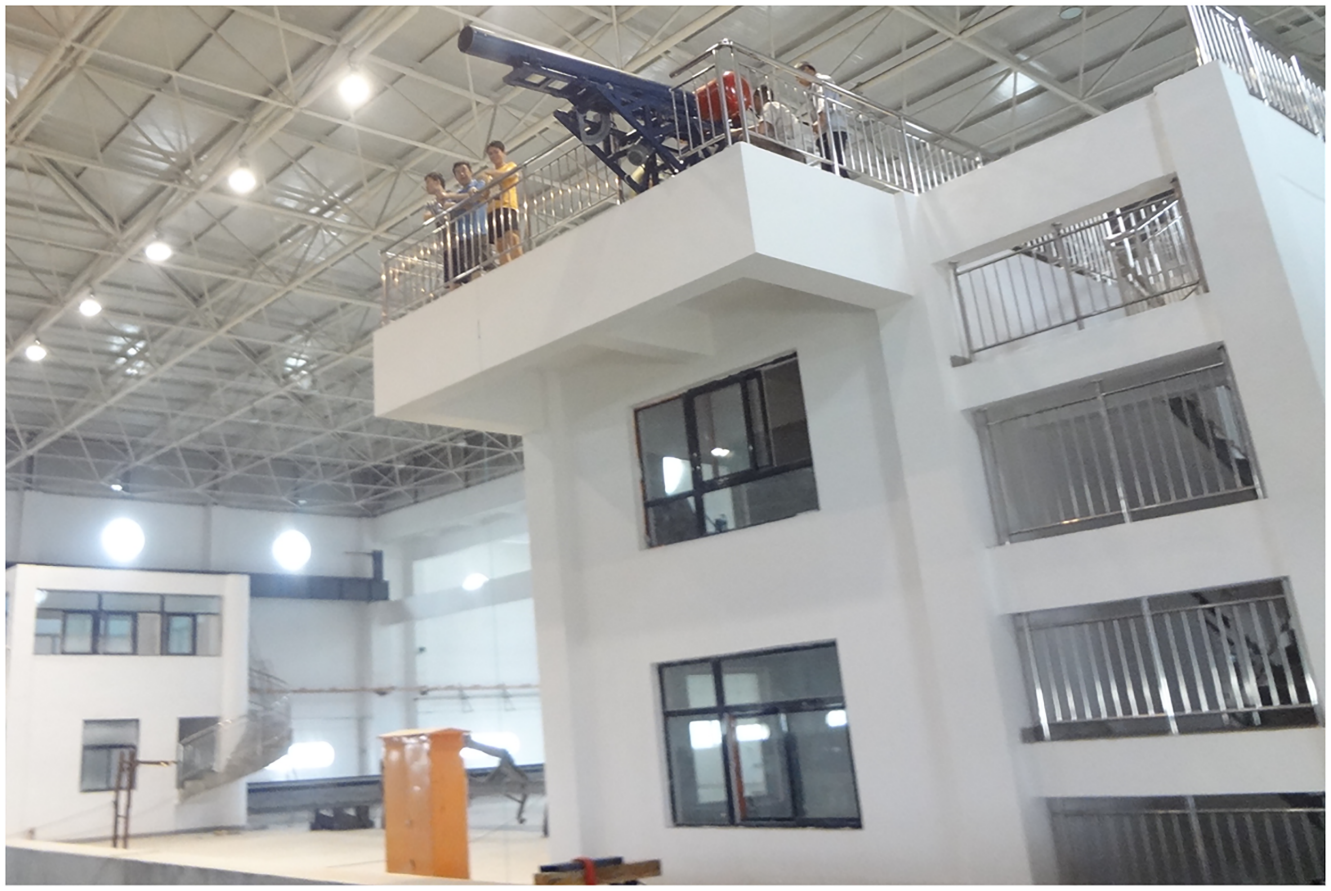
Launching platform.

The relevant required equipment during the experiment is described separately as follow. In the model launch system, the launch platform is located at one end of the deep water area of the pool, and at the top of a two-story building relative to the pool platform, as shown in Fig 7

**Fig 8:**
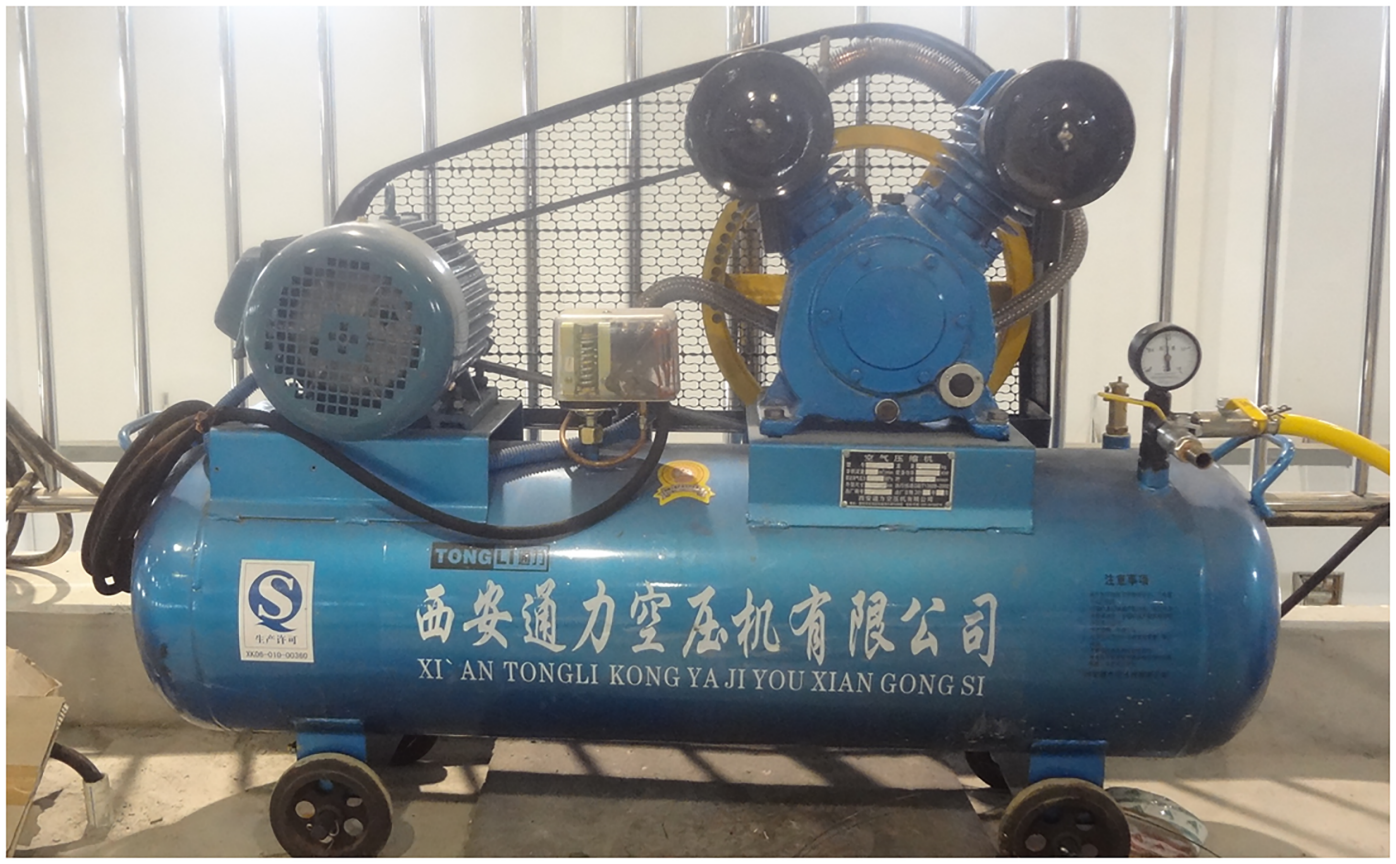
Air compressor.

The launch platform is mainly composed of air compressor, air gun, launching tube, launching cradle and track. The air compressor is shown in Fig 8. Its main function is compressing air, which inflates the air gun and provides a certain pressure air source. It can provide compressed air with the maximum pressure of 1.8Mpa.

**Fig 9:**
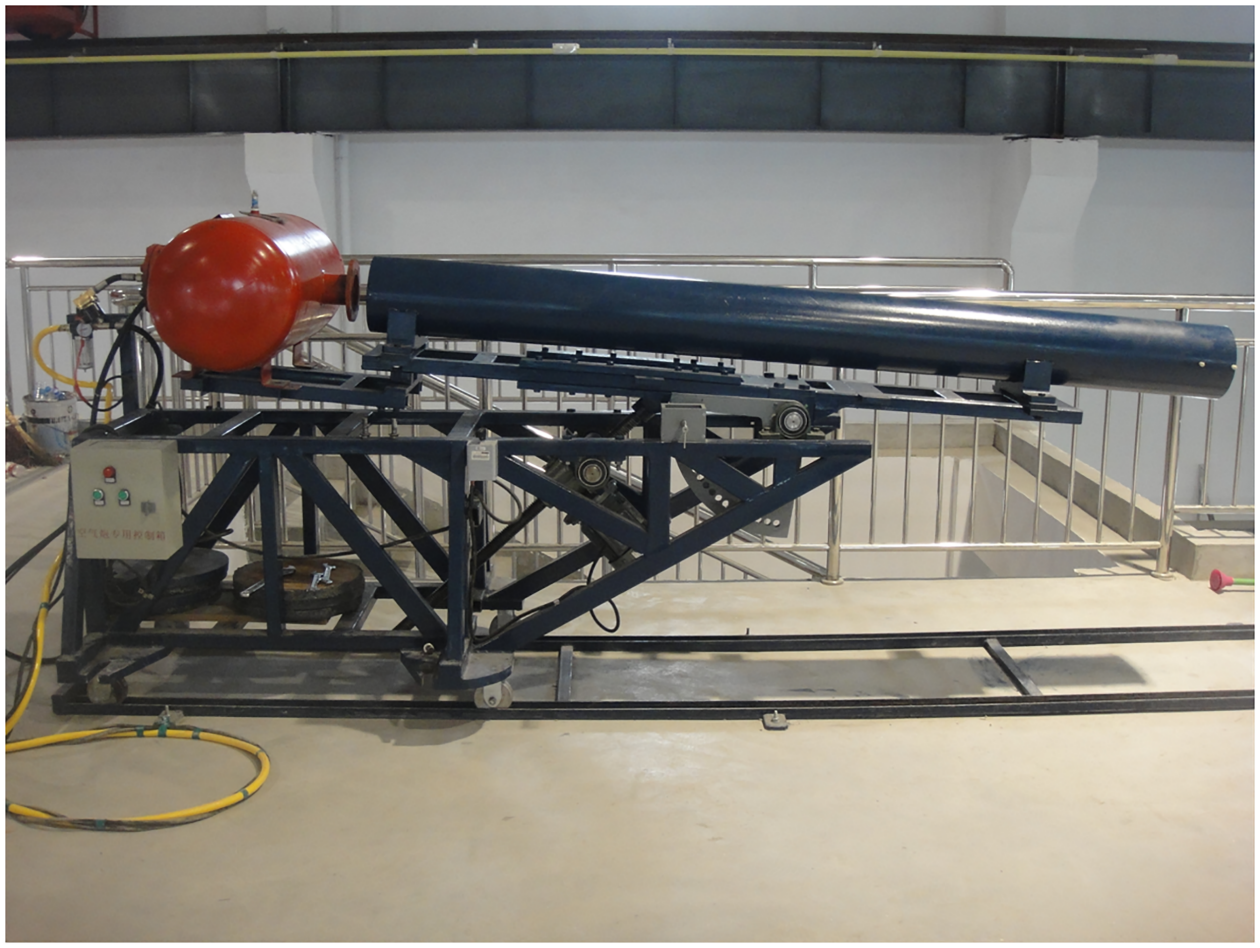
The overall structure of the spray gun.

The air gun and the launching tube form the spray gun as shown in Fig 9. It mainly stores the high-pressure air for launching the model. It can store up to 0.8Mpa air pressure, and the volume of it is 10L. The firing port of the air gun has a solenoid valve control switch, and directly connected with the launching tube. The launching tube is 2.5m length, 205mm diameter, so that it just can be loaded with 200mm diameter model. The lower part of the launching tube is connected to the launching cradle through the motor shaft and the rotating shaft. The motor and the shaft can control the launching angle of the launching tube. Through the control of the motor lift switch, the rough angle adjustment can be achieved. And through manual fine-tuning, the specific angle adjustment can be also achieved.

**Fig 10:**
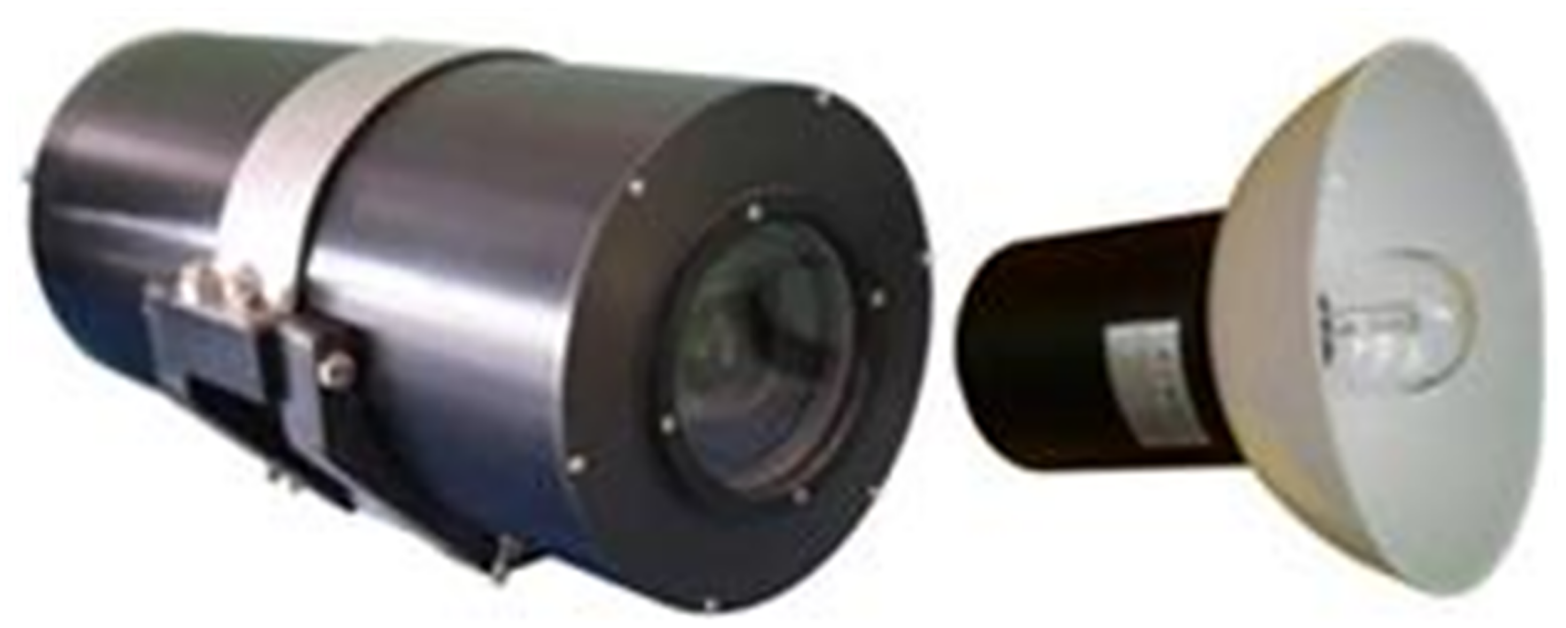
SXGS-1 underwater high speed camera and underwater lighting.

In the experimental observation system, the ordinary high-speed camera using the United States Phantom-v2511 high-speed digital camera, which erected in the experimental pool shore, can shoot the entire changing process of the attitude and trajectory of the vehicle from launching to enter into water. The underwater high-speed camera system is mainly used for underwater high-speed photography of the model at the time of water-entry, and to transmit, process and record the captured image. The whole underwater high-speed camera system consists of high-speed image transmission and processing system (display and control console), SXGS-1 underwater high-speed camera (shown in Fig 10), data cable, control cable and underwater bright LED lighting. And all whole control and data processing of the underwater high-speed camera are completed on the display and control console.

**Fig 11:**
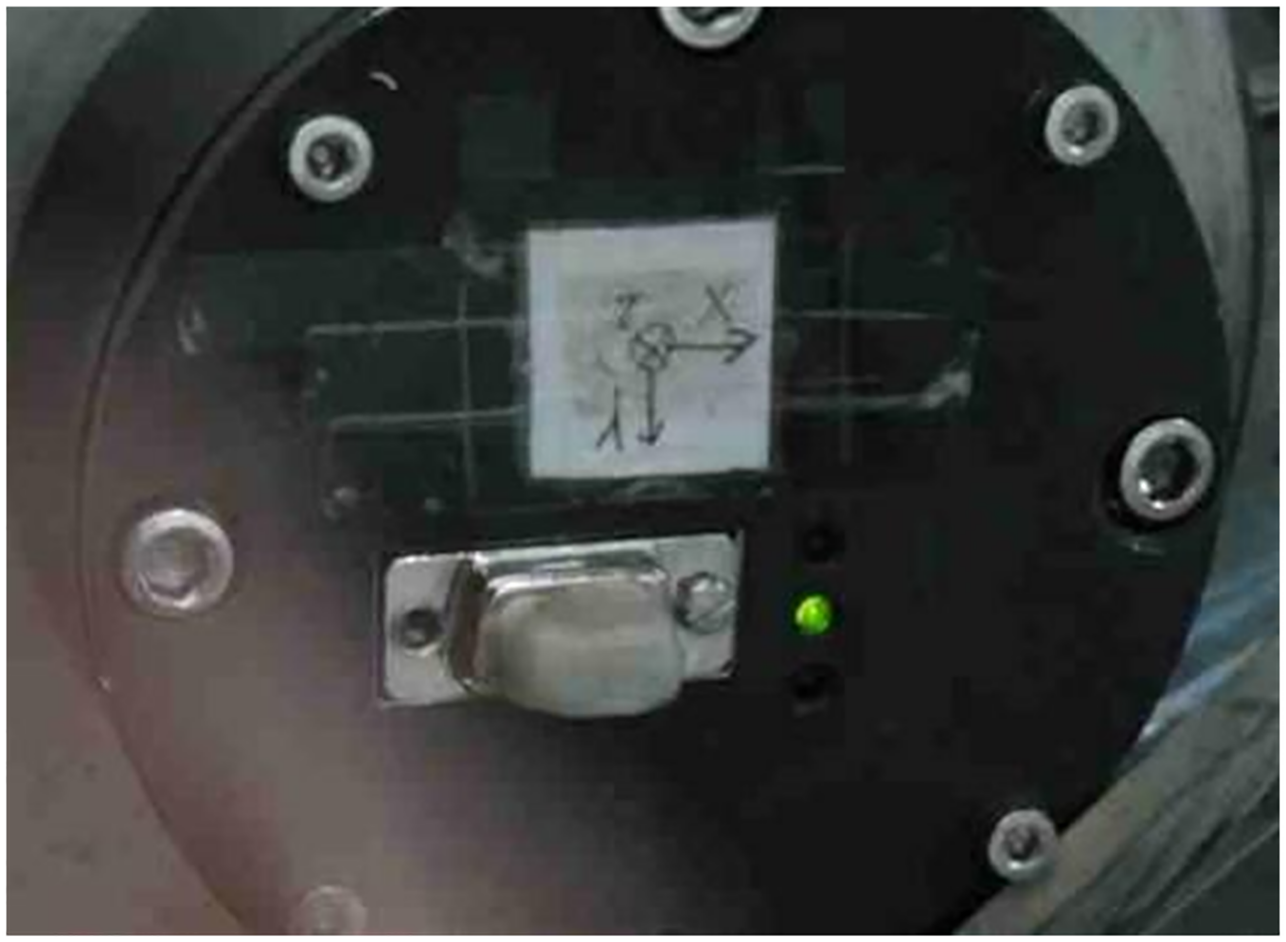
Acceleration sensor recorder.

In the data acquisition system, the used acceleration sensor recorder is shown in Fig 11. Acceleration sensor recorder is installed in the head of the experimental model, and the battery is put inside it. The measuring range of it is 2000g, the random error is about 1g, and the sampling frequency is 60 kHz.

### Experiment data processing

#### Image data processing

Image data from the onshore high-speed camera was processed by analyzing the actual trajectory of the experimental vehicle in the air from the images, obtaining the necessary trajectory data, and estimating the state of motion of the experimental model at the moment of water entry. Images were sampled every 50 frames (Δ*t* = 0.05s)from the 1000 frames per second taken by the high-speed camera to obtain the coordinates (*X*_*head*_, Y_*head*_) and (*X*_*tail*_,*Y*_*tail*_) of the head and tail of the experimental model relative to the exit of the launch tube. The coordinate values of the center of gravity (*X*_*c*_, *Y*_*c*_) and the pitch angle *θ* of the experimental vehicle at different moments were calculated according to the relative position of the center of gravity on the model and the coordinate values (*X*_*head*_, *Y*_*head*_) and (*X*_*tail*_, *Y*_*tail*_). Then, by ignoring air resistance and assuming that the model is only subjected to gravity in the air, the relationship between the coordinate value of the center of gravity, inclination angle, and initial state of in-air movement was obtained; on this basis, the initial state of air movement (*X*_*0*_,*Y*_*0*_,*v*_*x*0_,*v*_*y*0_,*θ*_*0*_,*ω*_0_) was calculated via the linear regression algorithm. Finally, the state of motion of the model (*X*_*in*_,*Y*_*in*_,*v*_*xin*_,*v*_*yin*_,*θ*_*in*_,*ω*_in_) was calculated at the moment of waterentry*t*_0_. The horizontal velocity *v*_*xin*_ = *v*_*x*0_, vertical velocity *v*_*yin*_ = *v*_*y*0_ + *gt*_*in*_, position coordinates *X*_*in*_, *Y*_*in*_, inclination angle *θ*, and rotating angular velocity *ω*_*in*_ = *ω*_0_ were obtained from the sampled images at the moment of water-entry.

The changing process of the state of the experimental model in water-entry motion reflected by video data from the underwater high-speed camera was analyzed to determine accurate changes in the position and attitude of the model during the water-entry process. Given that the underwater camera has a limited field of view through which the model moves for only a short time, the video was sampled every five frames(Δ*t* = 0.005*s*).

**Fig 12:**
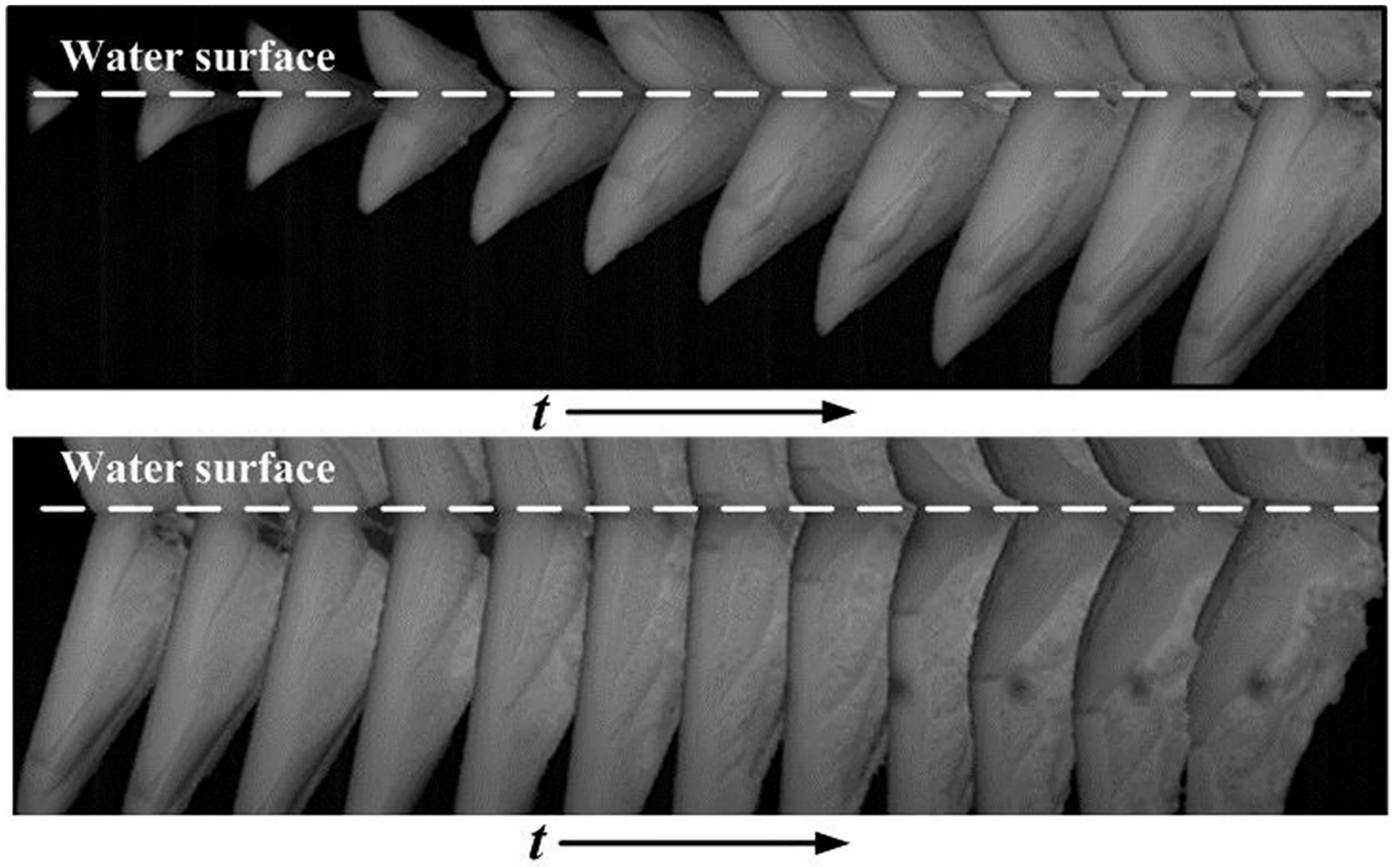
Underwater image of the whole water-entry process of the model.

Sampled images of the entire water-entry process were pieced together to create a full picture of the process; an image of the time-varying attitudes of the model were drawn accordingly, as shown in Fig 12.

**Fig 13:**
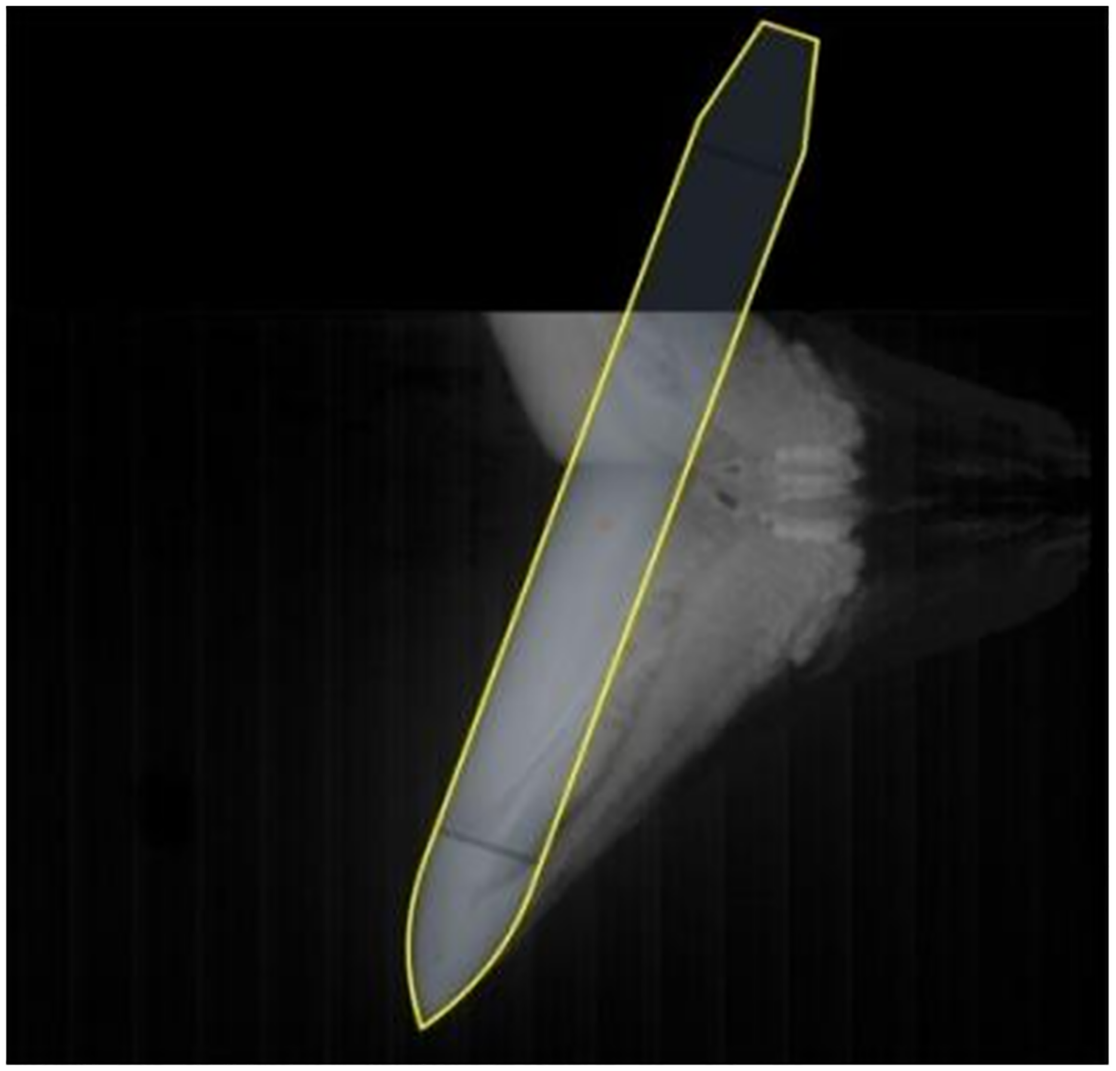
Determination of the outline size of the model.

**Fig 14:**
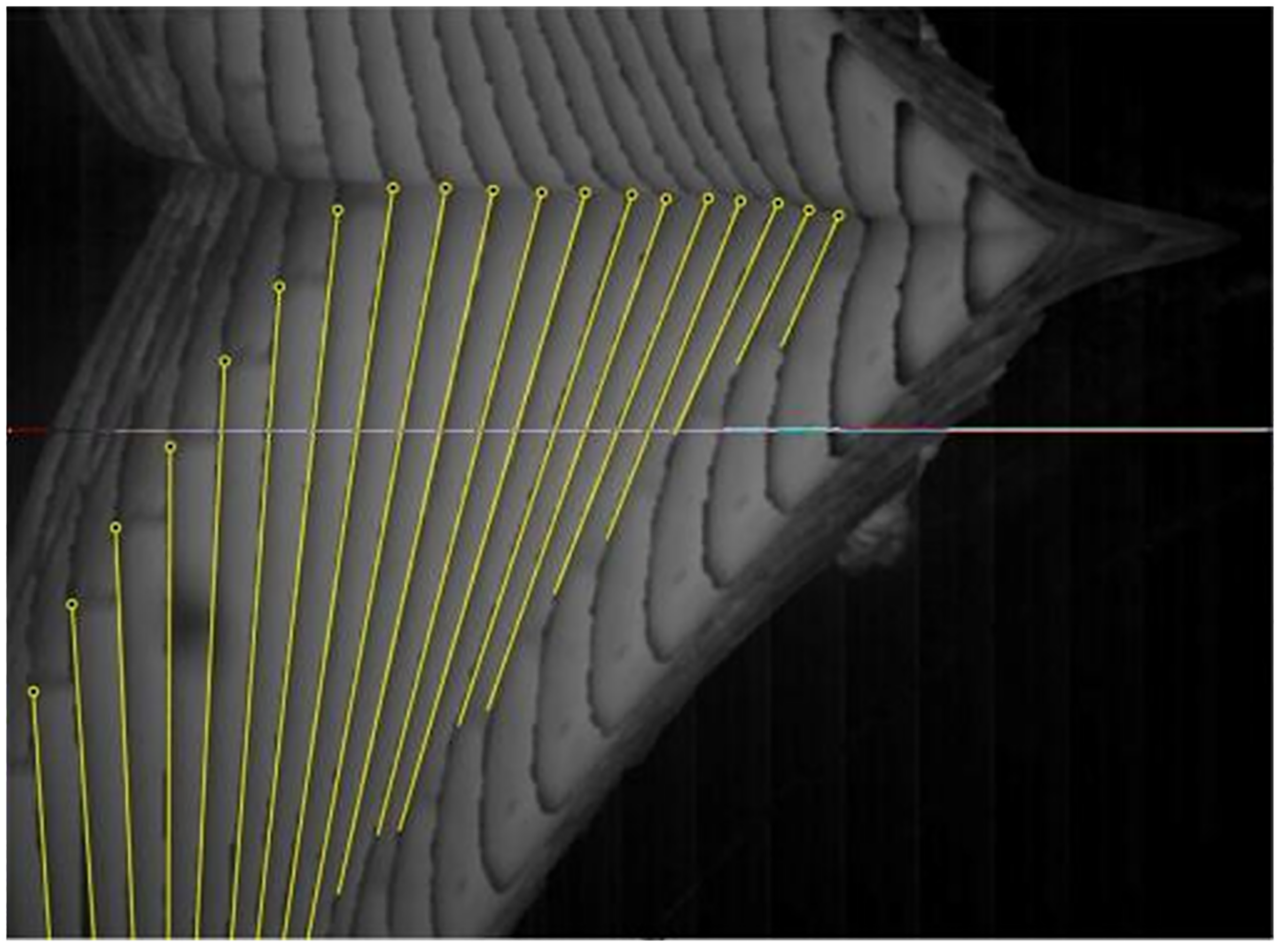
Determination of the tilt angle of the model.

**Fig 15:**
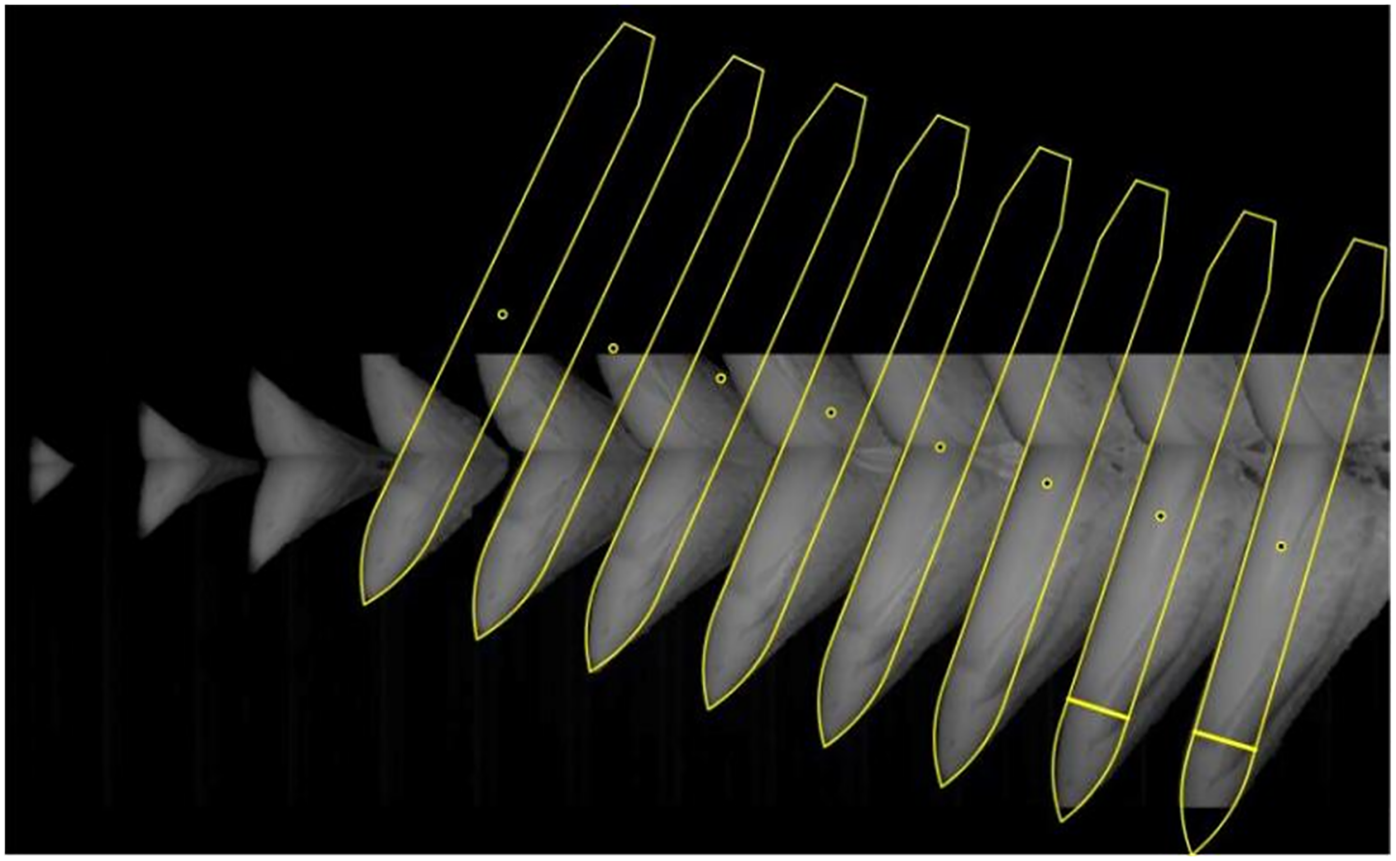
Determination of the position of model at the moment of water-entry.

The attitude and position of the center of gravity of the model were determined at each moment by analyzing the sampled image data. The most complete sample image was used as a reference. According to the proportion of the different parts of the model, an outline of the model as a whole was created as shown in Fig 13. Subsequently, the frontal profile of the model which is clear in the images was used to as a reference in analysis to determine the inclination angle of the model from the series of images as shown in Fig 14. The length of the model in the first three frames of the images is short, thereby causing difficulty in identifying the inclination angle; consequently, the data processing can result in error. Therefore, the first three frames were neglected during data processing. Finally, the position and the attitude of the model were located according to the outline size and inclination angle as shown in Fig 15. The position of the center of gravity of the model was also obtained by ratio scale conversion.

#### Acceleration sensor data processing

The acceleration sensor records the variations in the impact load of the model during the entire process from launching to entering water. This study specifically focuses on the behavior of the vehicle right after crossing the water surface. The underwater camera data show that the entire water-entry process took less than 0.1 s. Thus, this study focused on analyzing the acceleration data near that time period. The acceleration data within 0.2 s after water-entry was cut out to be investigated.

### Comparison and analysis of experimental and simulation results

The results of the experimental and simulated water-entry characteristics under the same initial conditions were compared to confirm the effectiveness of the established model.

According to the requirements of the experiment and the actual installation position of the underwater camera in the experiment pool, the condition that the launching angle is 30°0°and the pressure is 0.7Mpa is selected as the initial condition of the water-entry experiment of the vehicle. Under this condition, three water-entry experiments were carried out and three groups of the experimental results were obtained. After the data processing, comparing the three experiments under the same condition, the difference between the obtained changing trajectory of the inclination angles and the centre-of-gravity positions as well as the acceleration sensor data of the vehicle in three experiments is relatively small. Therefore, this paper selects one of the three groups of experimental data to carry out the model validation.

Experimental data with launch angle of 30° and pressure of 0.7 Mpa were selected for comparison. Under these launch conditions, by the method of data processing described above, the initial state of the vehicle at the moment of water-entry included the rotating angular velocity, horizontal velocity, vertical velocity, and inclination angle were obtained. And the inclination angles of the vehicle and the positions of the center of gravity that change with time were also obtained. The coordinate value of the center of gravity was obtained by treating the lower left corner of the underwater camera field of view as the reference origin and converting the scale as necessary.

**Fig 16:**
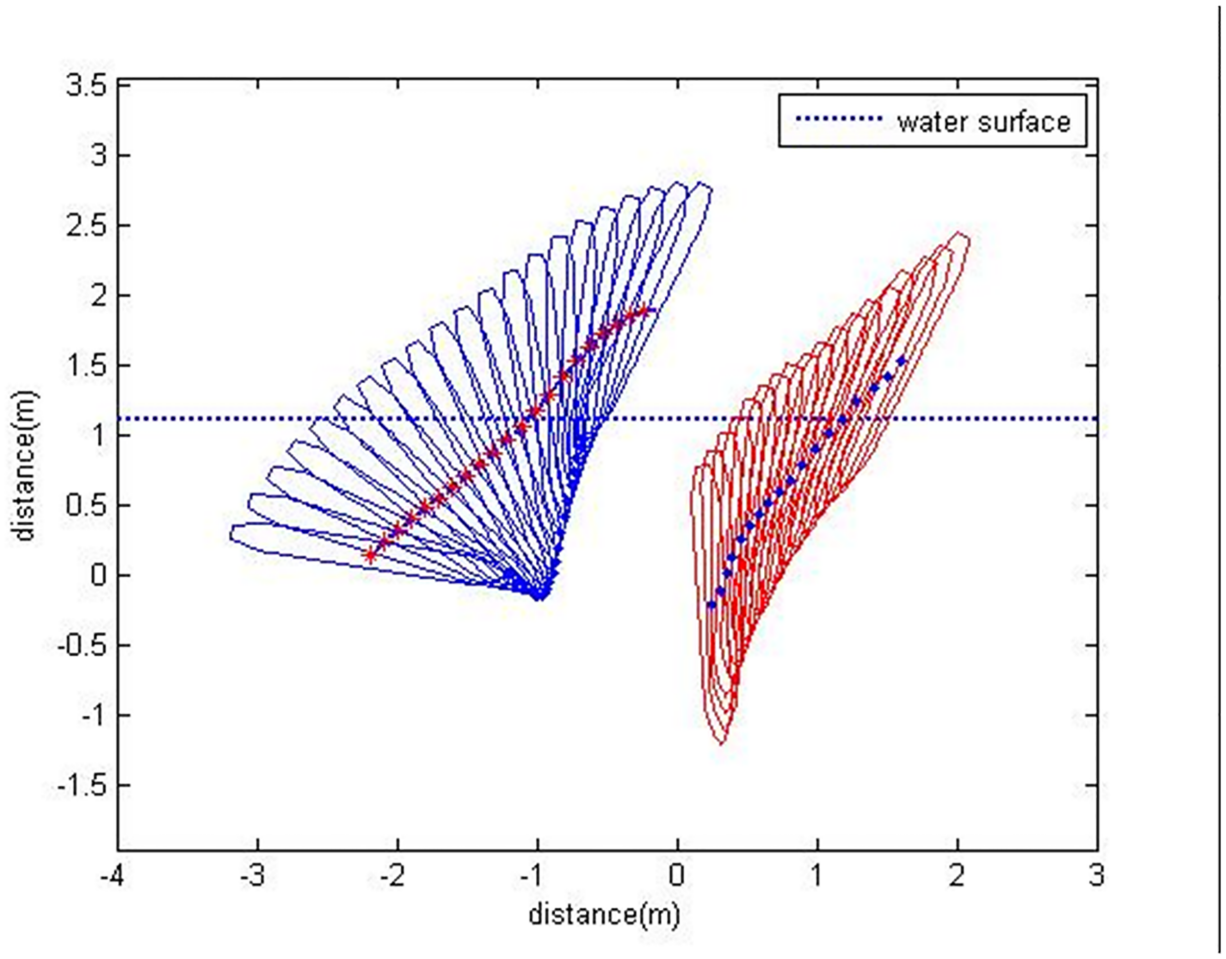
Comparison of the water-entry trajectories between experiment and simulation.

A 0.15 simulation was run to determine the changes in attitude and position of the vehicle during water-entry under the same conditions as the experiment for comparison. The simulation results were graphed in the same manner as the experimental results after processing the coordinate transformation to facilitate the comparison (Fig 16). The water-entry trajectory of the vehicle drawn by the inclination angle and the coordinates of the center of gravity in the experiment are on the right side of the figure; the water-entry trajectory obtained from the simulation is on the left side of the figure.

**Fig 17:**
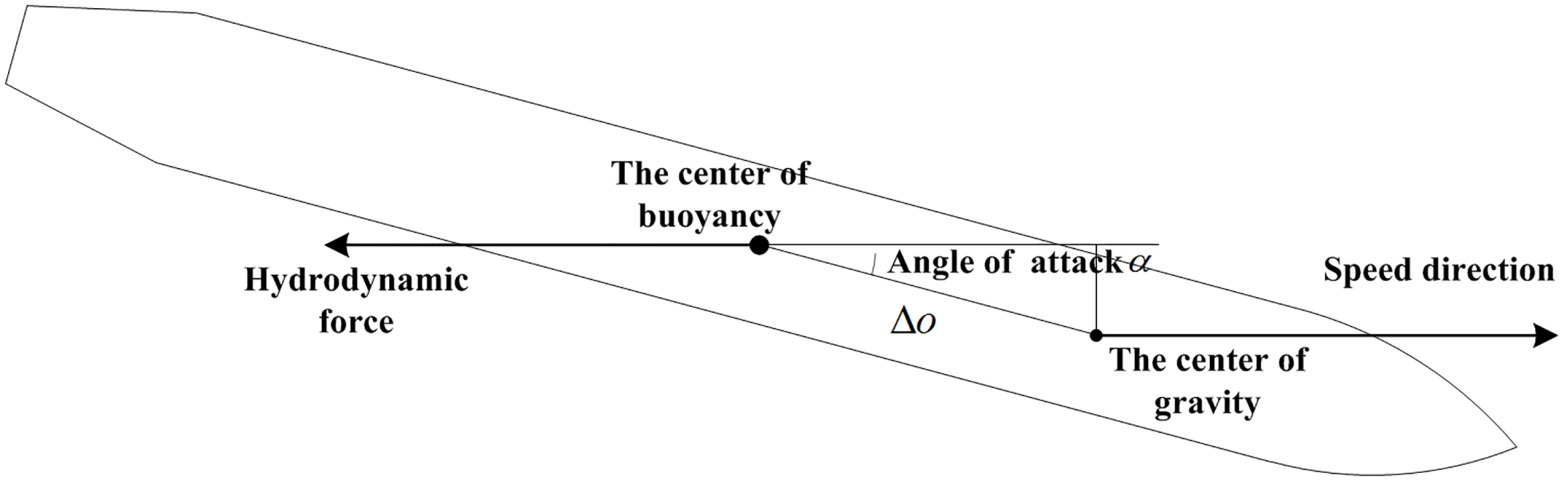
Sketch diagram of the center of gravity and the center of buoyancy.

The figure shows notable differences between the simulation and experiment. Most importantly, the changes in inclination angle are not uniform. The inclination angle obtained via simulation changes rapidly, this is, the vehicle rotates more intensely in the simulation. This finding can be attributed to a non-uniform density distribution of the experimental vehicle. Such distribution, forced the center of gravity to become close to the head, thereby leading to a moment produced by the hydrodynamic force. This phenomenon restrained the rotation of the vehicle after it entered the pool. Conversely, in modeling of the water-entry, the center of gravity of the vehicle was assumed to be located at its geometric center. Thus, when the vehicle is fully submerged, the hydrodynamic force does not produce any moment that restrains vehicle rotation. The rotation moments caused by the non-uniform center of gravity and center of buoyancy are shown in Fig 17.

**Fig 18:**
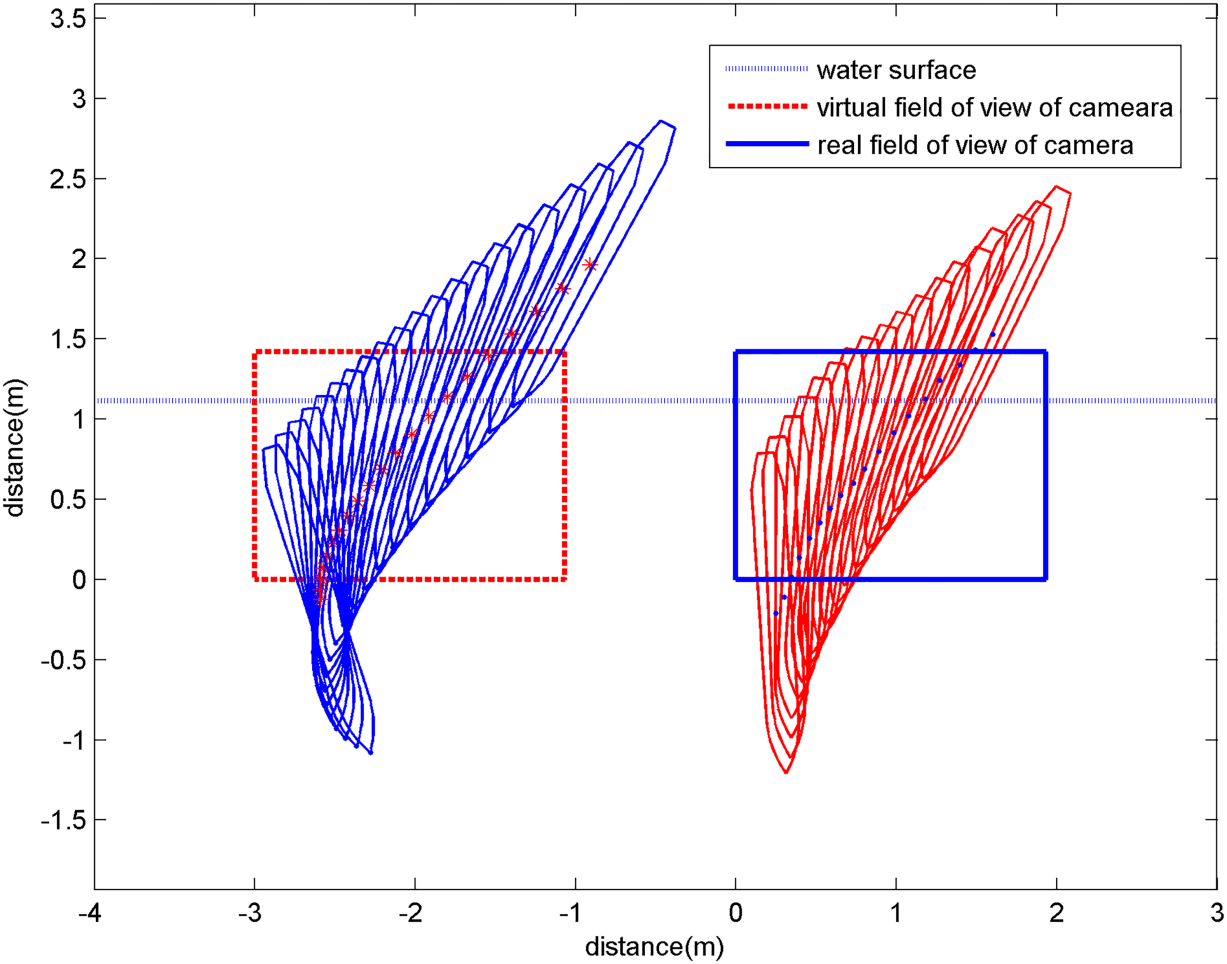
Comparison of the water-entry trajectories.

When the vehicle moves in the direction shown in Fig 18, the vehicle is subjected to hydrodynamic forces acting on the center of buoyancy; the force is opposite to the direction of the vehicle. When the vehicle has a certain angle of attack, this force produces a moment rotating around the center of gravity. The water-entry dynamic model was improved to account for this phenomenon. The rotation moment in Eq (28) was rewritten as follows:

$$\{\begin{array}{c} F_{\mu x}=-F_{\mu }\cdot \text{cos}\alpha \\ F_{\mu y}=F_{\mu }\cdot \text{sin}\alpha \\ M_{\mu z}=F_{\mu y}\cdot \left(l_{a}/2+\Delta o\right) \end{array}$$ (29)

**Fig 19:**
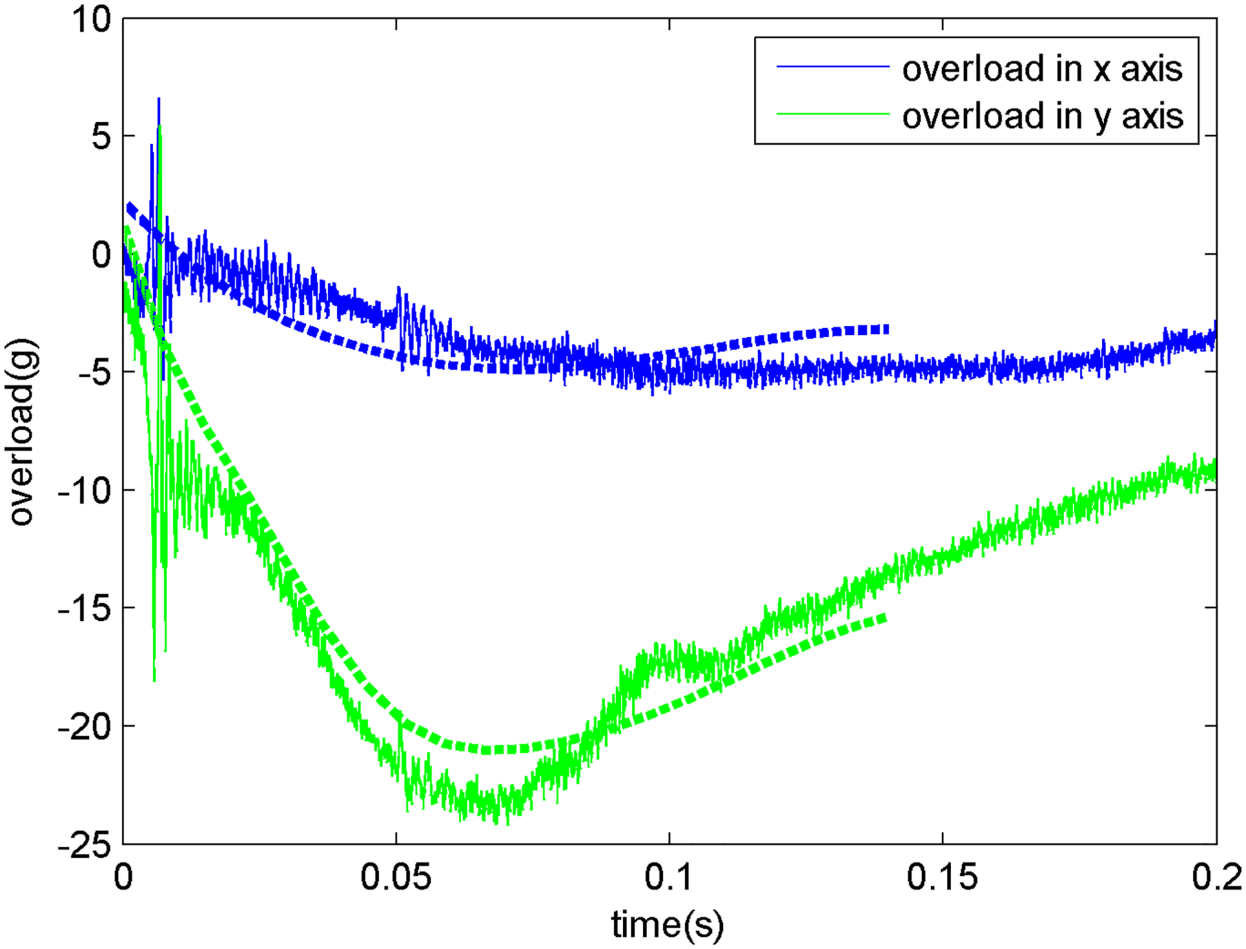
Comparison of the water-entry overloads.

The simulation was run again with the modified model, and the results were again compared with experimental results as shown in Fig 18. And based on the relative position between the camera itself and the water-entry point of the model, the underwater real shooting field (right side) and the virtual shooting field (left side) were ascertained as the figure showed. And the Fig 19 shows the comparison between the overload of the vehicle in the water-entry process and recorded data from the accelerometer. The hopping data also shown in the figure were recorded by the accelerometer. The simulation results create a smoother curve than the experimental results and are represented as a dotted curve.

**Fig 20:**
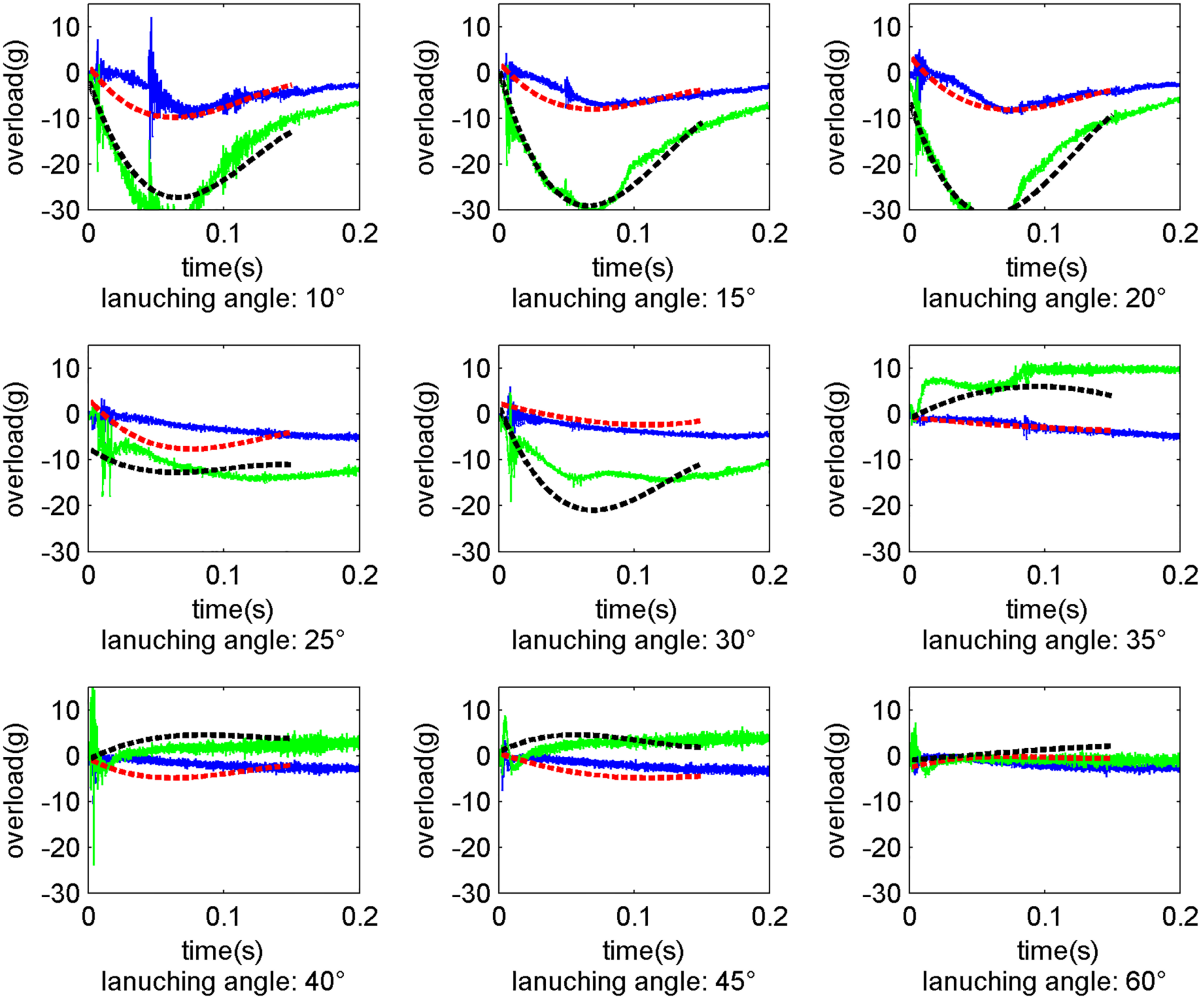
Comparison of the water-entry overloads in different conditions.

Under the same initial water-entry conditions, the change laws of the center of gravity, attitude, and overload in the water-entry process are basically consistent between the experimental and simulated results. The corresponding initial conditions of the water-entry were set up and re-examined to further verify the accuracy of the model. The overload recorded by the acceleration recorder and the overload as-calculated via the proposed model are shown in Fig 20, where launching pressure is 0.6 Mpa and the launching angles are 10°, 15°, 20°, 25°, 30°, 35°, 40°, 45°, and 60°. The hopping data with jitter characteristics were recorded by the accelerometer. Again, the smooth data were calculated by model simulation. And in the figure, for the simulation result, the red dotted curve indicates the overload in the x-axis direction and the black dotted curve indicates the overload in the y-axis direction.

In summary, the overload value calculated by the theoretical model is consistent with the overload value obtained via experimentation. This consistency further verifies the accuracy of the dynamic model proposed in this study. The proposed model can indeed be utilized to analyze the water-entry motion laws of the trans-media vehicle, although some notable errors are observed in the figures. These errors were caused by two main factors: 1) Inevitable issues with the data acquisition and processing, and2) perturbation of the water surface and the influence of splashing and cavity in the water-entry process were neglected in the proposed model while the viscous hydrodynamic coefficients were obtained by numerical simulation and interpolation.

## Model simulations

On the basis of the established model, a series of simulations was run and compared to analyze the motion laws of the vehicle.

### Simulation 1: Effects of initial water-entry velocity

**Fig 21:**
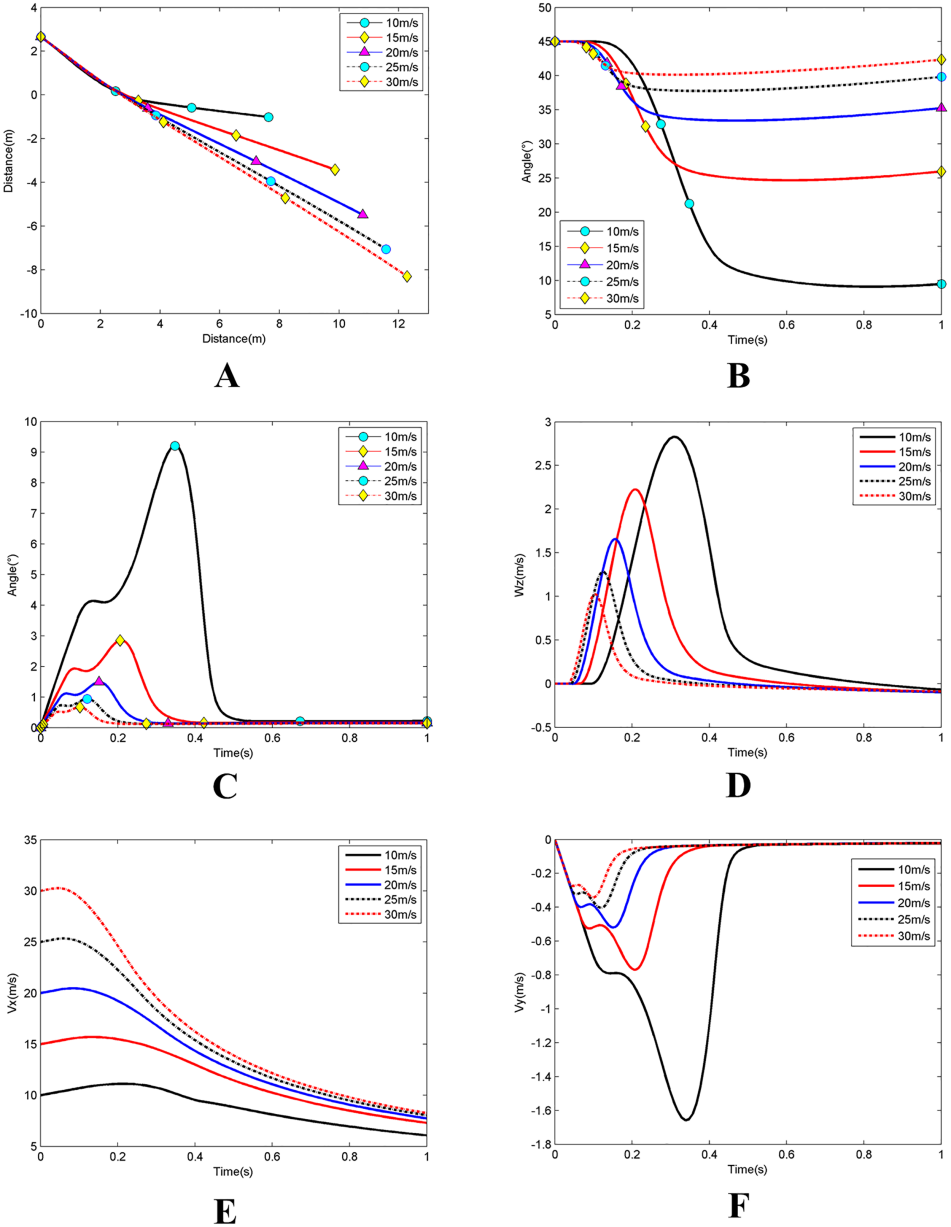
Effects of initial water entry velocity. **(A)** The change of the barycentre trajectory;**(B)** The change of the inclination angle; **(C)** The change of the angle of attack; **(D)** The change of the rotating angular velocity; **(E)** The change of the axial velocity; **(F)** The change of the radial velocity.

Simulation 1conditions included initial water entry angle *θ*_0_ = 45°; initial position *x*_*a*0_ = −1m; initial angle of attack *α*_0_ = 0; initial rotating angular velocity *ω*_*z*0_ = 0. Then, the initial water entry velocity was set to be gradually increased from 10m/s to 30m/s by 5m/s. Fig 21 shows the simulation results.

As shown in Fig 21, with same water entry angle, high initial entry speed yields significant change in axial velocity but insignificant changes in radial velocity and vehicle attitude (including inclination angle and rotating angular velocity). Thus, the change of the corresponding angle of attack is smaller. The trajectory is highly stable under these conditions, and the water depth and horizontal displacement are relatively large.

At the beginning of the process, at a certain some distance from the top of the vehicle to the surface of the water and is only subject to gravity, the axial and radial velocities increase within a small margin while a small forward angle of attack is formed. With same water entry angle, the axial distance from the top of the vehicle to the surface of the water is equal. As the initial water entry velocity increases, the moment at which the vehicle first touches the water become earlier; the time it spends under the action of gravity become shorter; the increments of angle of attack, axial velocity, and radial velocity decrease, and the trajectory, inclination angle, rotating angular velocity remain unchanged. When the vehicle begins to touch the surface of the water, the normal force and the upward moments at the lower surface of the head of the vehicle form. At this moment, the angle of attack, axial velocity, and radial velocity continue to increase, the rotating angular velocity begins to increase, the inclination angle begins to decrease, and the trajectory begins to curve upward. A greater the water-entry velocity means a short the time for the upward moment to act on the vehicle. Thus, based on the moment of momentum theorem, the obtained angular velocity becomes small. The changes in the inclination angle, angle of attack, axial velocity, radial velocity, and the extent of trajectory curving upward also become small. No upward moment is provided after the head of the vehicle has become completely submerged into the water. The vehicle continues to roll because of the previous rotation angular velocity. At the same time, the rotating angular velocity rapidly decreases, the radius of gyration increases, the trajectory tends to become straight, inclination angle continues to decrease, and the angle of attack, axial velocity, and radial velocity also rapidly decrease.

### Simulation 2: Effects of initial water-entry angle

**Fig 22:**
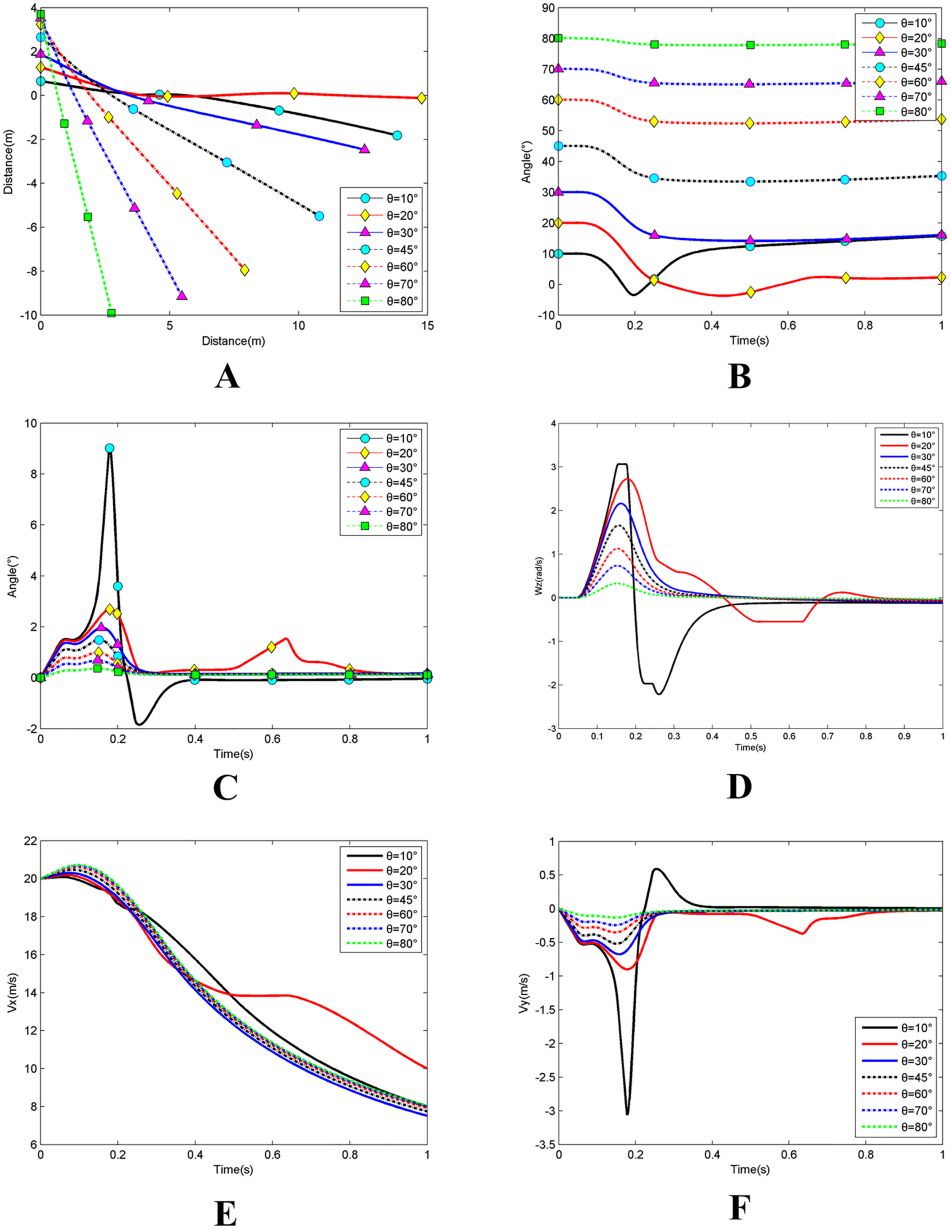
Effects of initial water entry angle. **(A)** The change of the barycentre trajectory;**(B)** The change of the inclination angle; **(C)** The change of the angle of attack; **(D)** The change of the rotating angular velocity; **(E)** The change of the axial velocity; **(F)** The change of the radial velocity.

Simulation 2conditions included initial angle of attack *α*_0_; initial position *x*_*a*0_ = −1m; initial water entry velocity *v*_0_ = 20m/s; initial rotating angular velocity *ω*_*z*0_ = 0. The initial water entry angle was set to be gradually increased from 10° to 80° by 10°. Fig 22 shows the results.

When the entry angles of the vehicle are 10° and 20°, the top of the vehicle is not yet fully submerged, whereas the tail has contacted the surface of the water. The two conditions make the water-entry process highly unstable. Thus, these conditions were neglected in this portion of our analysis. As shown in Fig 22, under the same water entry speed, when the initial water entry angle becomes large, the rotating angular velocity becomes small and the changes in inclination angle and attitude become small. The corresponding angle of attack and radial velocity also show small changes, whereas the axial velocity stays basically consistent. The trajectory easily keeps stable and is unlikely to curve, the water depth is larger, and the horizontal displacement is smaller under these conditions.

At the beginning of entering the water, the certain height from the top of the vehicle to the water surface and the axial distance are equal under the various water entry angles. Under the effect of gravity, if the water entry angle is large, then the force of gravity on the axial force component is also large, whereas the radial component force is small. Consequently, a larger increase in axial velocity and a small increase in radial velocity and angle of attack are observed. Meanwhile the trajectory, inclination angle, and rotating angular velocity remain unchanged. When the vehicle begins to touch the surface of the water, normal force and upward moments form underneath the top of the vehicle. Under the comprehensive effect of these factors, the angle of attack continues to increase, the rotating angular velocity begins to increase and the trajectory begins to curve upward. Then the inclination angle and axial velocity begin to decrease, and the radial velocity continues to increase. When water entry angle is large, the time necessary for the vehicle to be completely submerged after the top of the vehicle touches the water surface is truncated; that is, the time that the upward moment acts on the vehicle is also shortened. Based on the moment of momentum theorem, the obtained rotating angular velocity of the vehicle is small while the changes in inclination angle, angle of attack, axial velocity, and radial velocity are small and the trajectory is straight. After the top of the vehicle is completely submerged, the upward moment disappears. The vehicle continues to roll based on the previous rotating angular velocity, the rotating angular velocity, angle of attack, and radial velocity decrease to zero, the axial velocity decreases rapidly, the inclination angle tends toward a constant value, and the trajectory tends to become straight.

### Simulation 3: Effects of initial angle of attack

**Fig 23:**
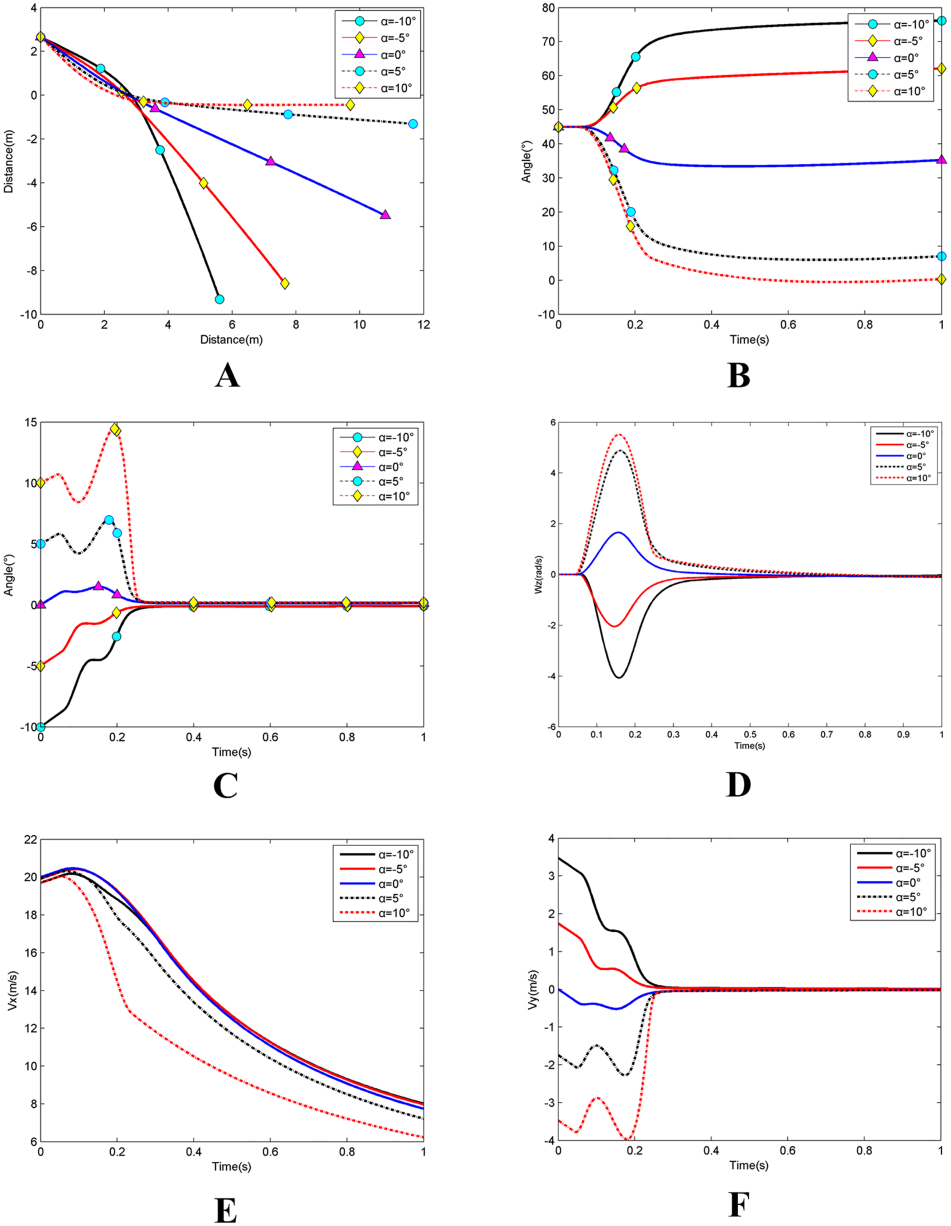
Effects of initial angle of attack. **(A)** The change of the barycentre trajectory;**(B)** The change of the inclination angle; **(C)** The change of the angle of attack; **(D)** The change of the rotating angular velocity; **(E)** The change of the axial velocity; **(F)** The change of the radial velocity.

Simulation 3 conditions included initial position *x*_*a*0_ = −1m; initial water entry velocity *v*_0_ = 20m/s; initial water entry angle *θ*_0_ = 45°; and initial rotating angular velocity *ω*_*z*0_ = 0. The studied the trans-media vehicle is similar with an air-dropped torpedo in the water entry process. The entry angle of attack should be relatively small to reduce the water entry impact of the internal structure; the initial angle of attack varied in this simulation from 0°, ±5°, and ±10°. Fig 23 shows the simulation results.

As shown in Fig 23, when the angle of attack *α* ≥ 0°, as it enlarges, the decreasing trend of the inclination angle and the positive direction rotating angular velocity both become significant. Meanwhile the changes in the corresponding angle of attack are large. The decreasing trend of the axial and radial velocities is also significant. Under these conditions, the trajectory is easily curved upward and the water depth and horizontal displacement are smaller.

When the angle of attack *α* < 0°, and as angle of attack enlarges, the increasing trend of the inclination angle and the negative direction rotating angular velocity both become significant. Meanwhile, the changes in the corresponding angle of attack are also significant. The decreasing trend of radial velocity is obvious, whereas the decreasing trend of axial velocity remains fairly stable. Under these conditions, the trajectory is more easily curved downward, the water depth is large, and the horizontal displacement is small.

When velocity is constant at the beginning of the water entry process, a large angle of attack results in a larger axial velocity and a small radial velocity. At a certain distance from the top of the vehicle to the water surface, and under the effect of gravity, a slight increase in axial velocity in the positive direction and a slight reduction in radial velocity in the negative direction occur, whereas a slightly positive angle of attack forms. Thus, when *α* ≥ 0°, the angle of attack, axial speed, and radial speed all increase to some extent while the trajectory curves upward; the trajectory curving becomes intense at a large angle of attack. When *α* < 0°, the axial velocity increases slightly, whereas the angle of attack and radial velocity decrease slightly and the trajectory curves downward to a great extent at a larger angle of attack. At the moment the vehicle contacts the water surface, normal force and upward moments form underneath the top of the vehicle. When *α* ≥ 0°, an upward fluid moment is created by the positive angle of attack. Under the common effect of two moments, the angle of attack first increases and then decreases as rotating angular velocity begins to increase. The trajectory continues to curve upward, the inclination angle decreases rapidly, and both axial and radial velocities begin to decrease. When *α* < 0°, a down ward fluid moment is created by the negative angle of attack; under the mutual effect of two moments, the angle of attack continues to decrease, whereas the rotating angular velocity begins to increase in the negative direction. As the trajectory keeps curving downward, the inclination angle increases, whereas the axial and radial velocities decrease. A great angle of attack creates large fluid moments and rapid changes in trajectory, inclination angle, attack angle, rotating angular velocity, axial velocity, and radial velocity. No upward moment occurs after the top of the vehicle is entirely submerged. Before the angle attack continues to decrease, the fluid moments decrease to zero, while the rotating angular velocity begins to decrease and the angle of attack, axial velocity, and radial velocity rapidly decrease. The inclination angle ultimately tends toward a constant and the trajectory gradually becomes horizontal and straight.

## Conclusion

A single control strategy composed of underwater non-control and in-air control was proposed in this study to avoid the difficulty in controlling trans-media vehicles during the crossing process. Thus, the water-entry process of the vehicle under the uncontrolled condition was mainly studied. By analyzing the characteristics of the water-entry process and introducing time-varying parameters (e.g., buoyancy and added mass), a suitable dynamic water-entry model of the vehicle at low speed was constructed to study the motion characteristics of the vehicle during water-entry. A low-speed water-entry experiment was conducted and the results were compared with results obtained by the proposed model. The model was modified slightly on the basis of comparison to ensure that the simulated results coincide with experimental result. Then the new dynamic model was utilized to study the motion laws of the vehicle in the water-entry process.

The conclusions of the study can be summarized as follows.

- In the water-entry process, the motion of the vehicle is significantly influenced by the initial state.
- A great initial velocity causes less intense changes in vehicle attitude during water-entry, stable trajectory, and large vertical and horizontal displacement.
- A large initial inclination angle creates a vehicle trajectory that is stable and less prone to curving as well as large vertical displacement and smaller horizontal displacement.
- The value and direction of the angle of attack play a significant role in the water-entry process. A large angle of attack creates intense changes in vehicle attitude and makes the trajectory prone to curving.

## Supporting information

S1 Table Experimental overload data of the vehicle. (XLSX)

S2 Table Experimental attitude and trajectory data of the vehicle. (XLSX)

S1 File Image data in the air of the experiment. (ZIP)

S2 File Underwater image data of the experiment. (ZIP)
