# Supplementary figures and images for: Simulation and experimental research on trans-media vehicle water-entry motion characteristics at low speed
Source: PLoS One. 2017 May 30;12(5):e0178461. doi: 10.1371/journal.pone.0178461 (PMC5448784; doi:10.1371/journal.pone.0178461)

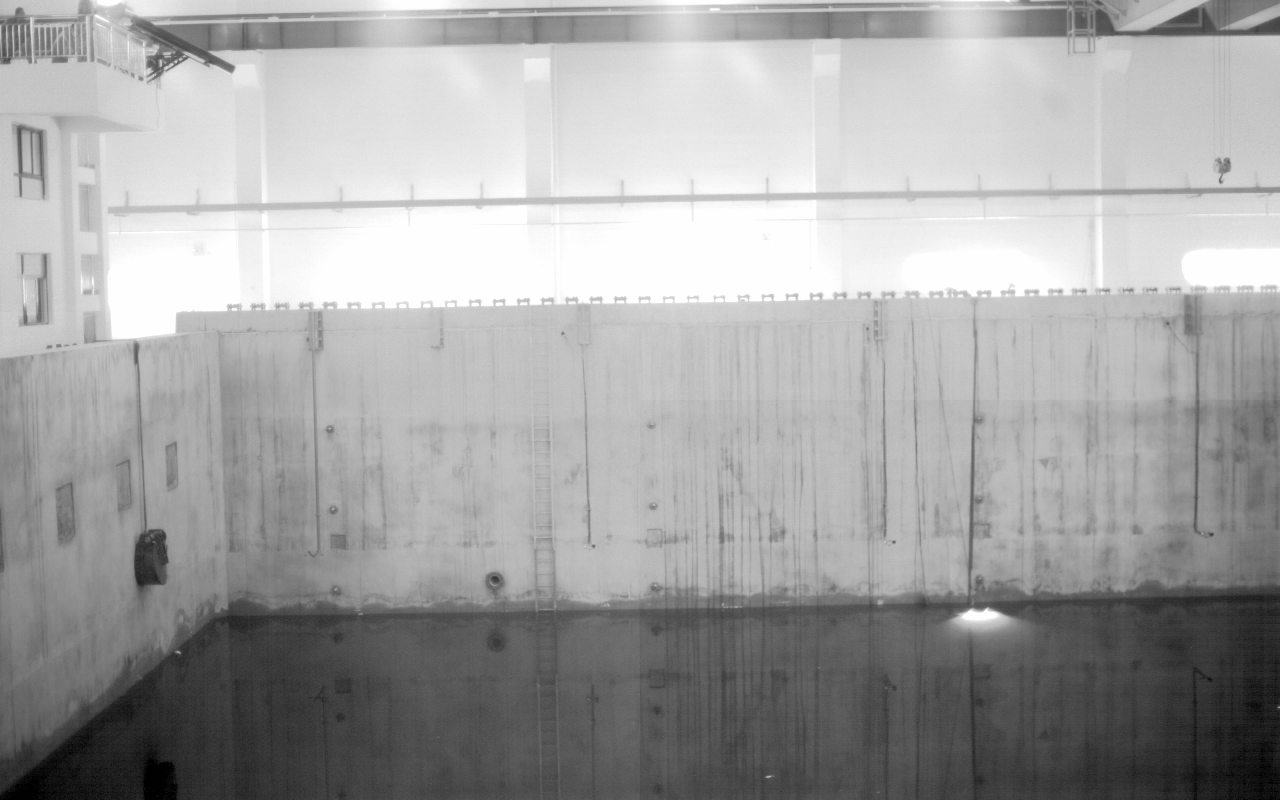

Supplement: S1 File — (ZIP) [file pone.0178461.s003.zip › image data in the air/1.tif]

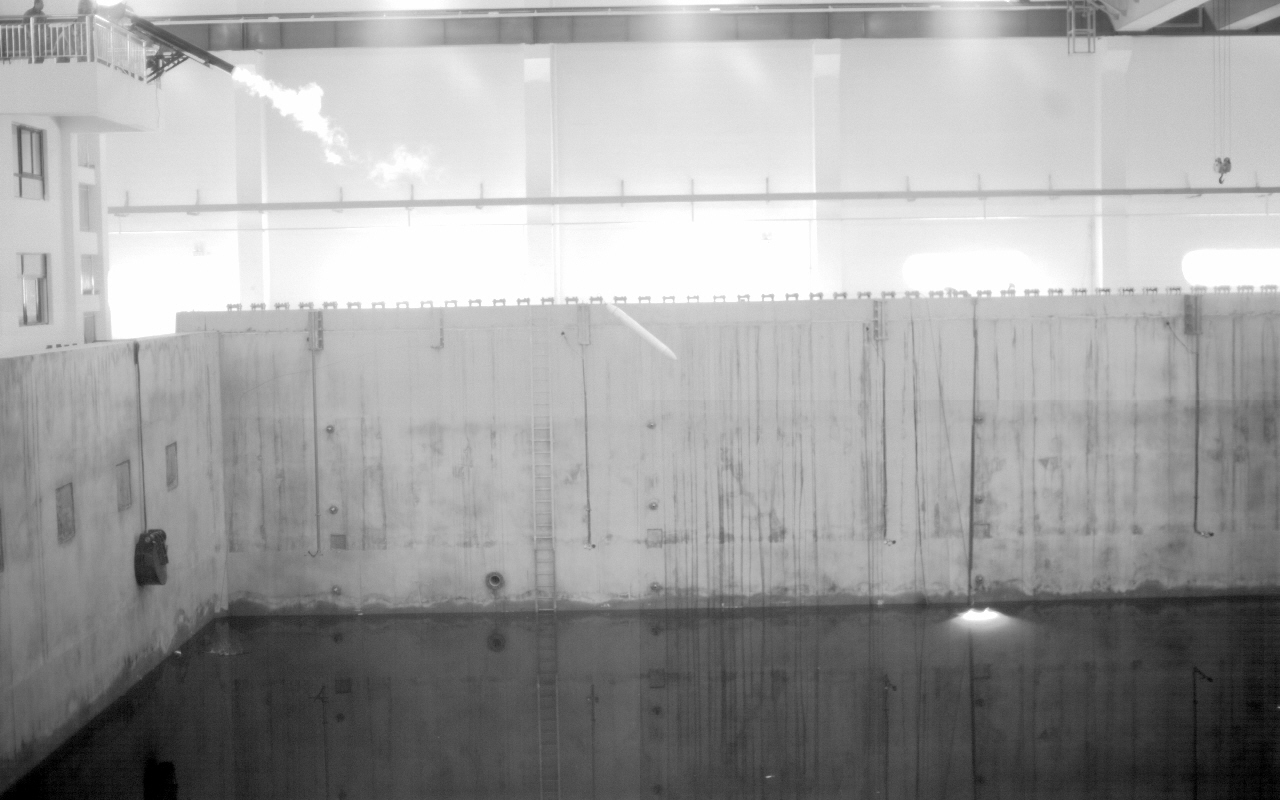

Supplement: S1 File — (ZIP) [file pone.0178461.s003.zip › image data in the air/10.tif]

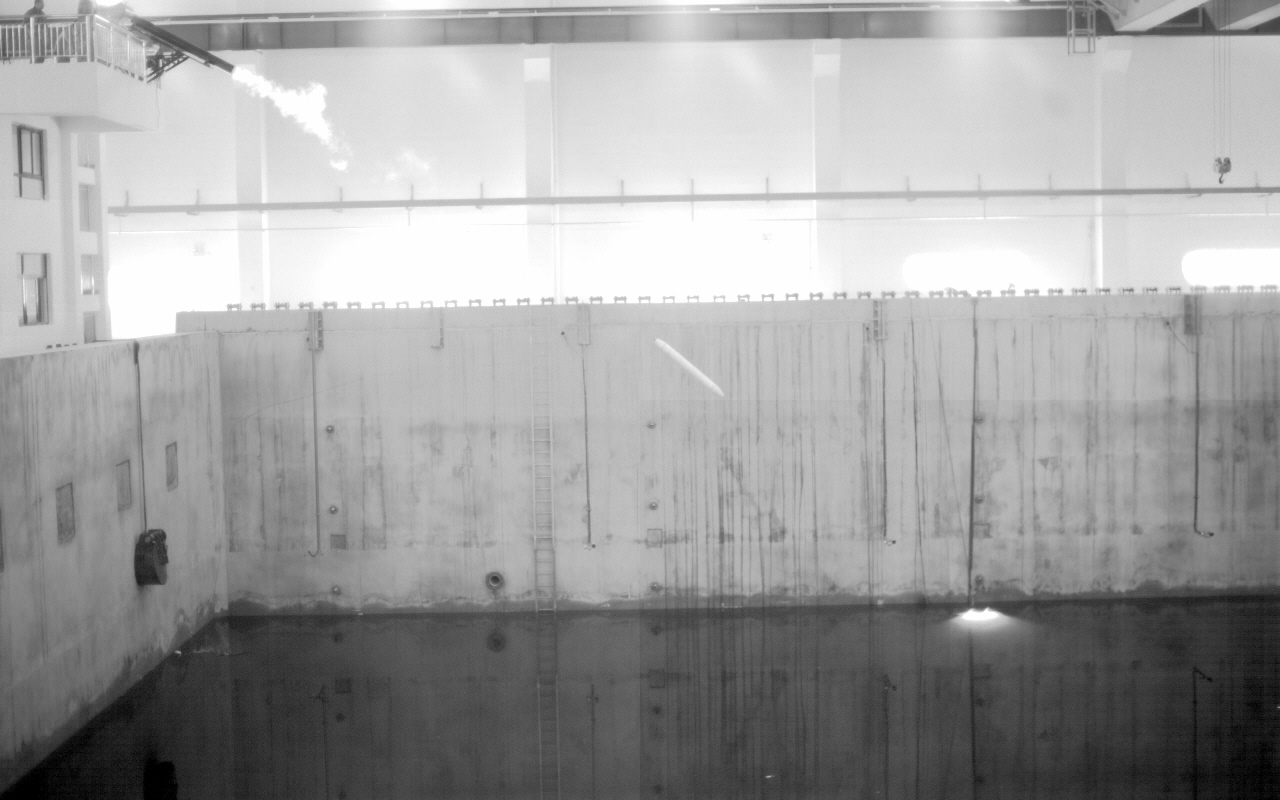

Supplement: S1 File — (ZIP) [file pone.0178461.s003.zip › image data in the air/11.tif]

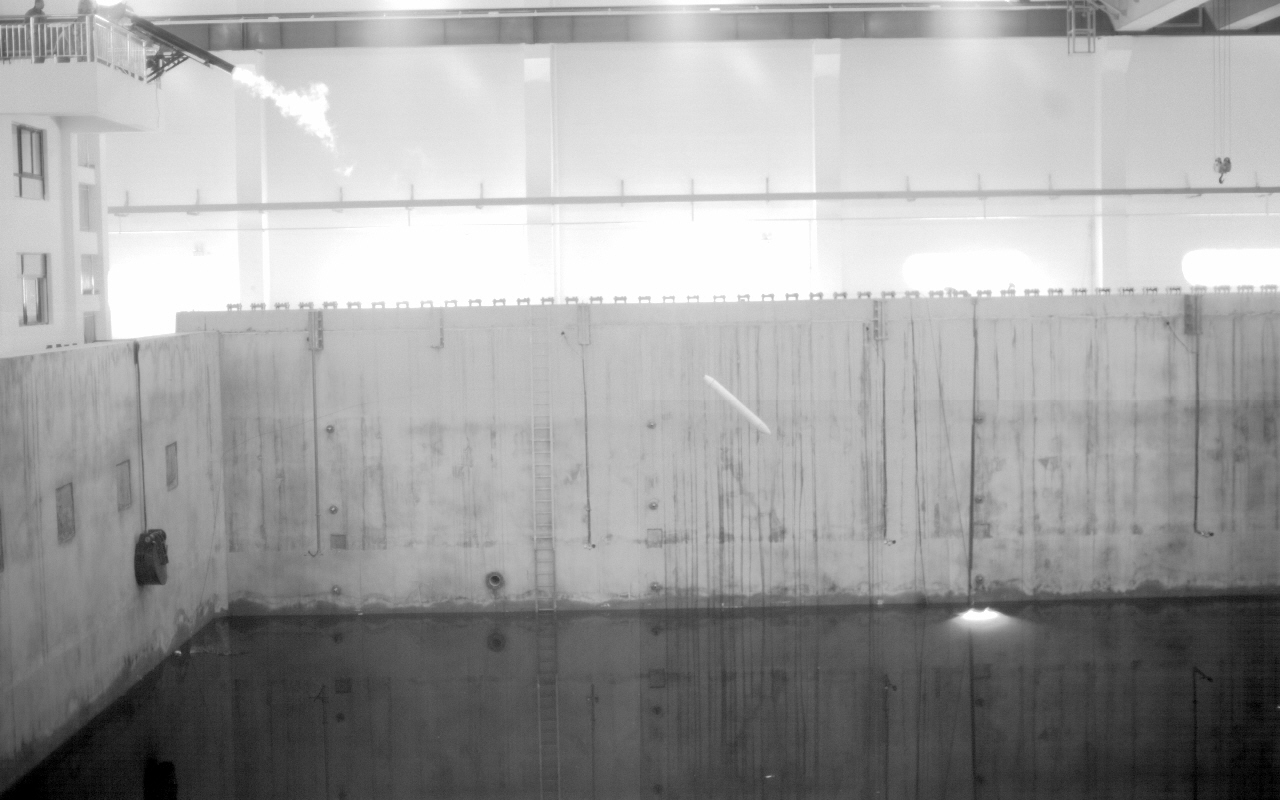

Supplement: S1 File — (ZIP) [file pone.0178461.s003.zip › image data in the air/12.tif]

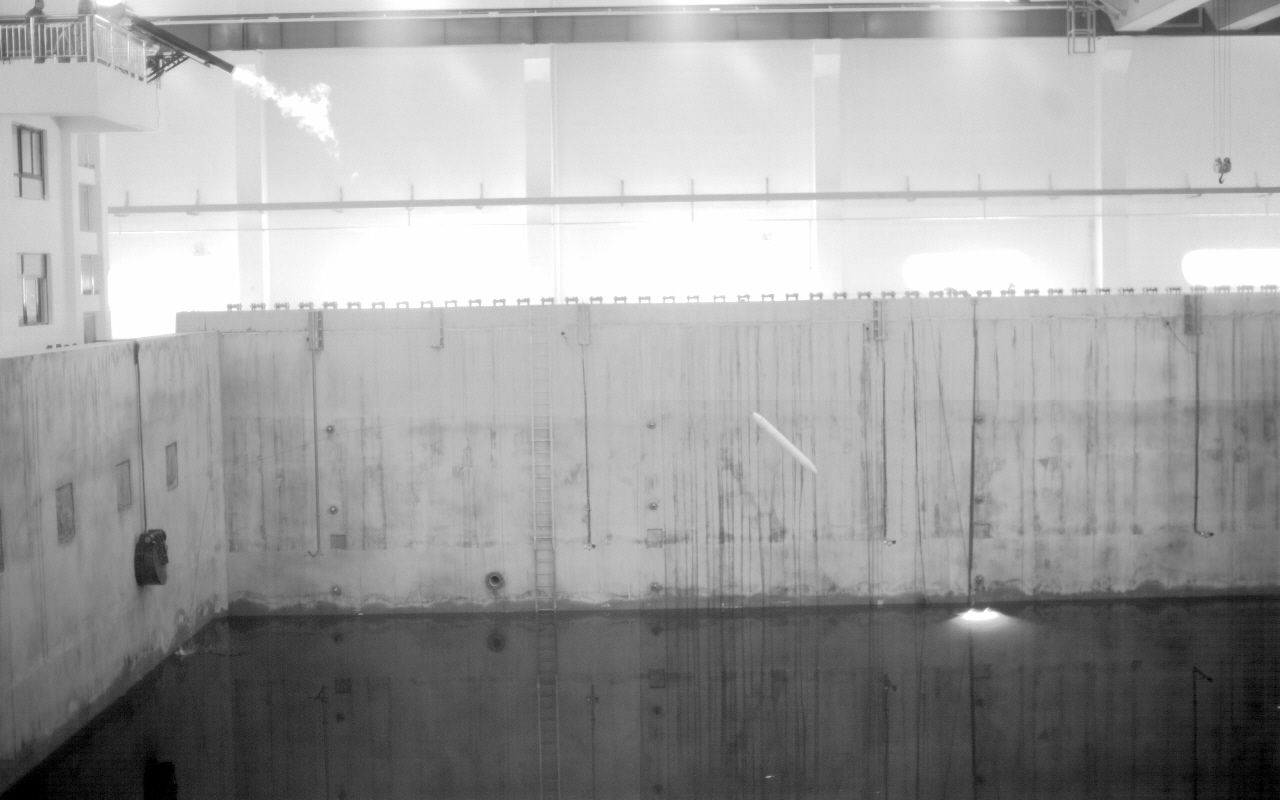

Supplement: S1 File — (ZIP) [file pone.0178461.s003.zip › image data in the air/13.tif]

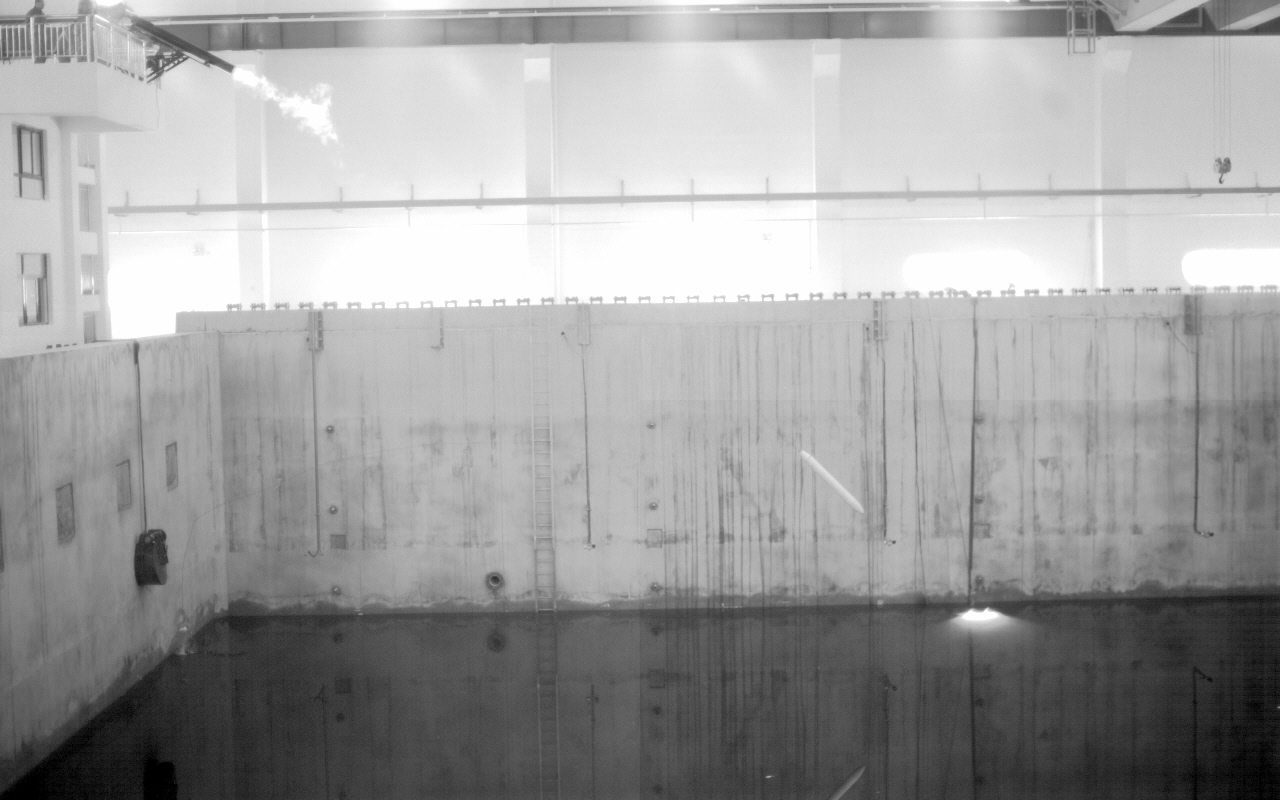

Supplement: S1 File — (ZIP) [file pone.0178461.s003.zip › image data in the air/14.tif]

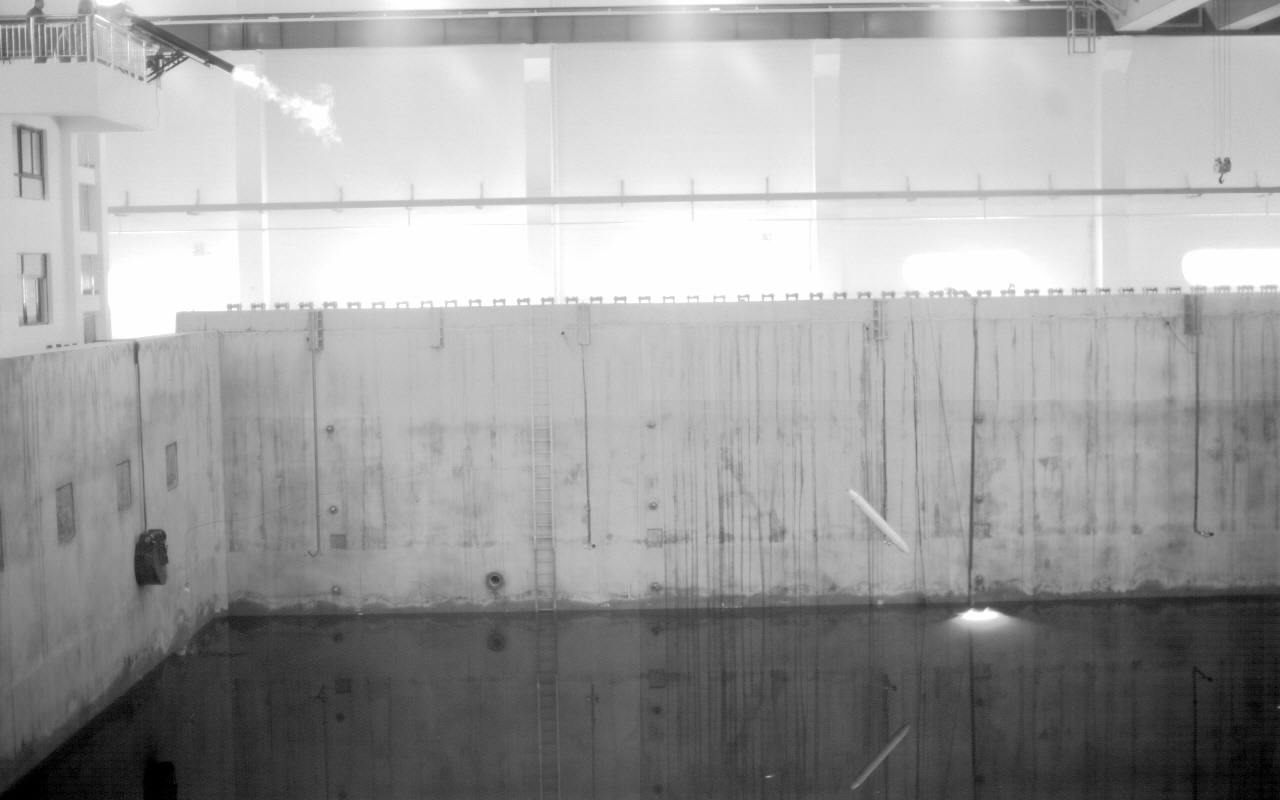

Supplement: S1 File — (ZIP) [file pone.0178461.s003.zip › image data in the air/15.tif]

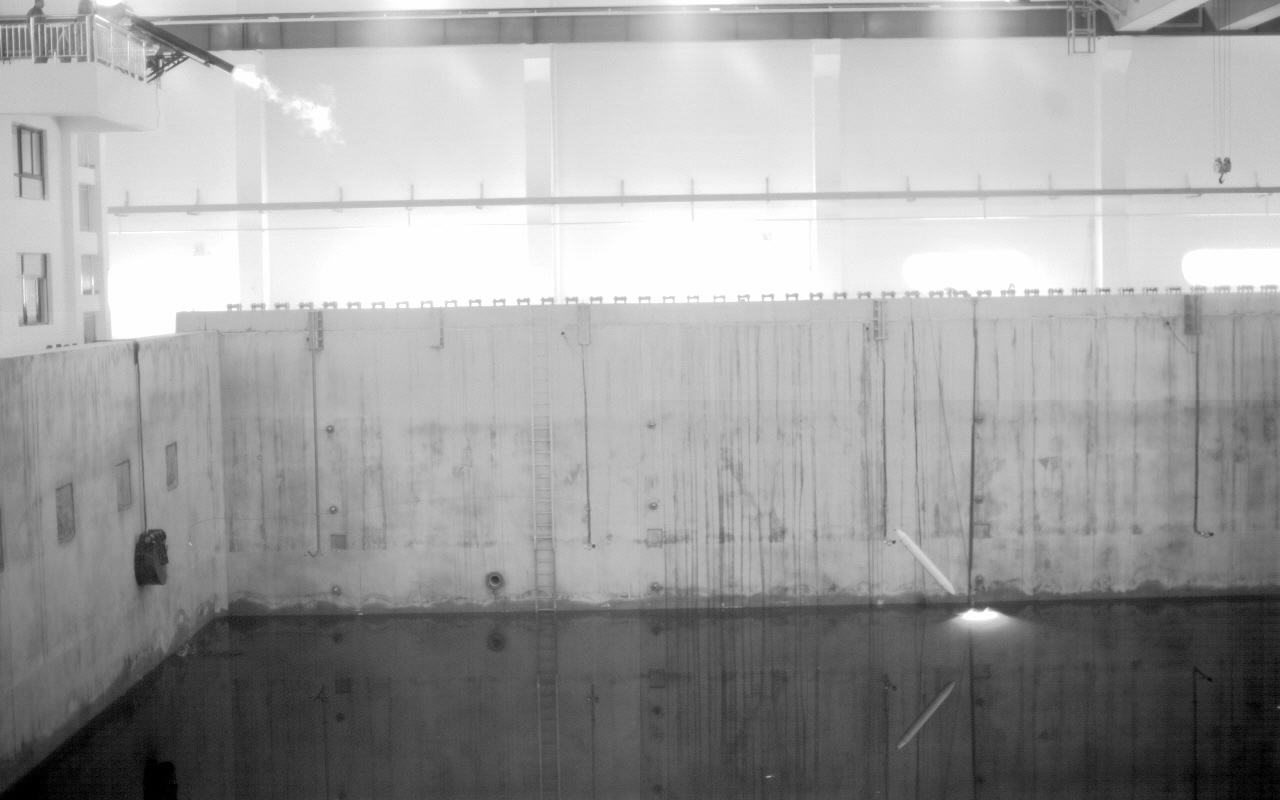

Supplement: S1 File — (ZIP) [file pone.0178461.s003.zip › image data in the air/16.tif]

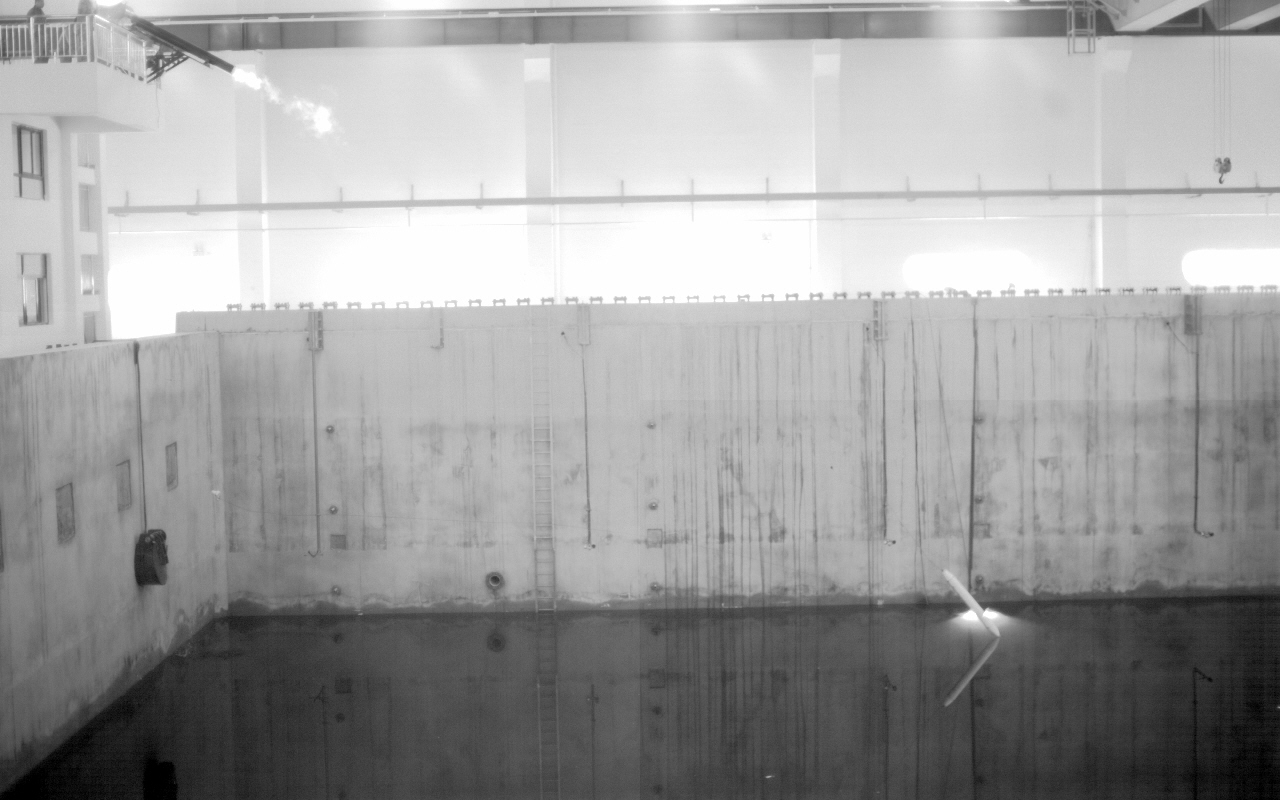

Supplement: S1 File — (ZIP) [file pone.0178461.s003.zip › image data in the air/17.tif]

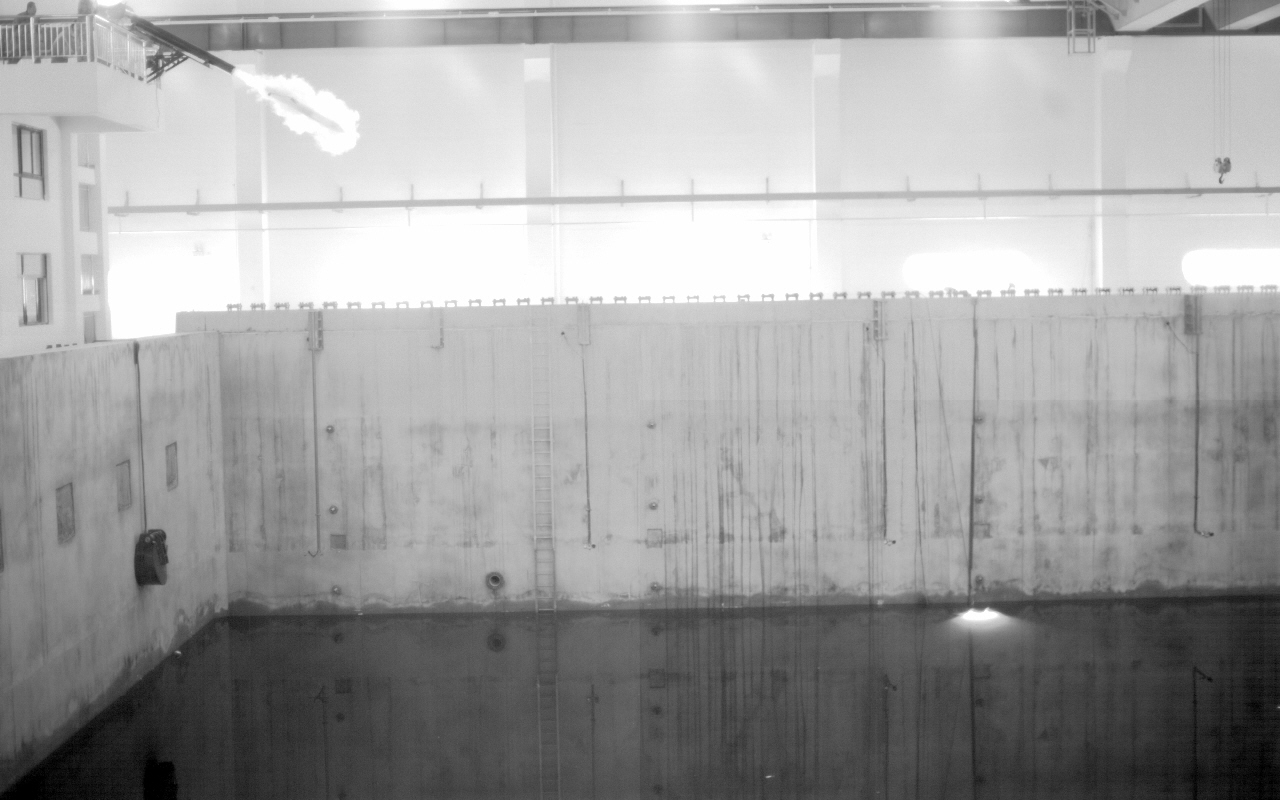

Supplement: S1 File — (ZIP) [file pone.0178461.s003.zip › image data in the air/2.tif]

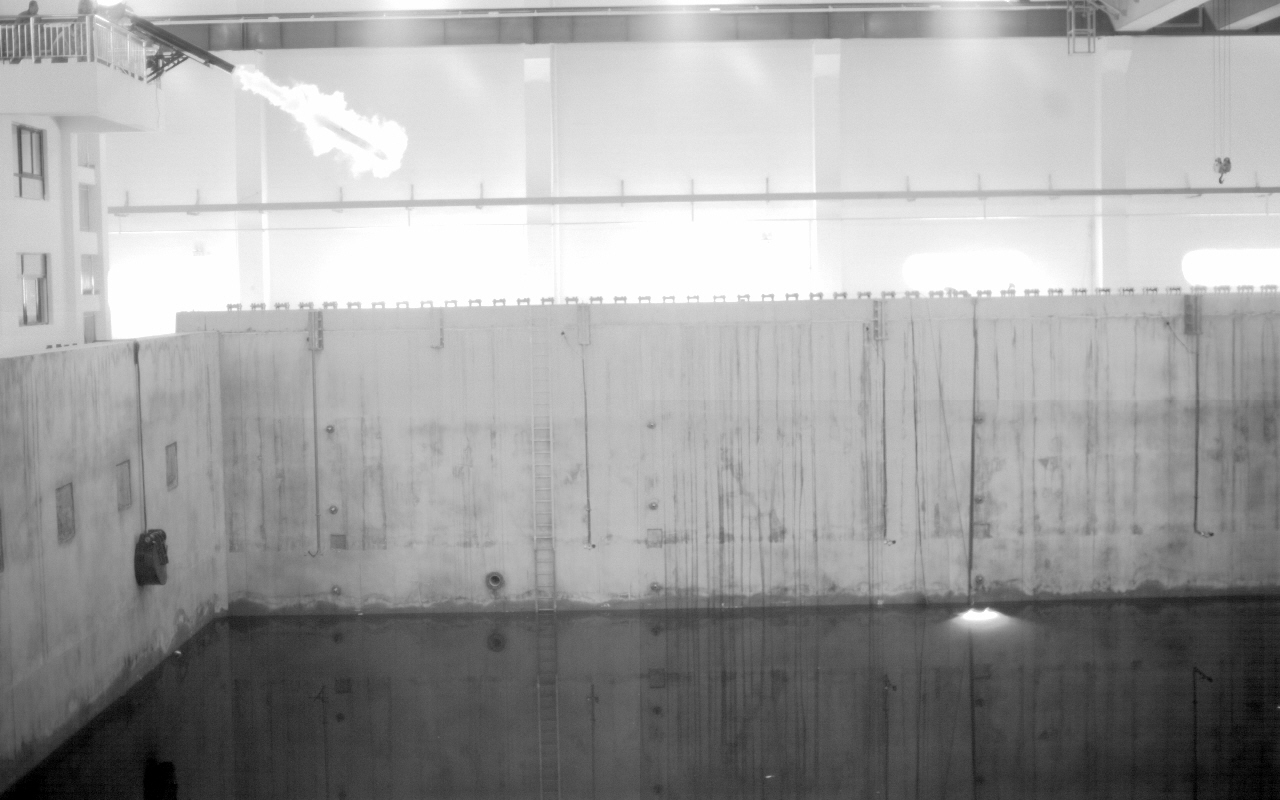

Supplement: S1 File — (ZIP) [file pone.0178461.s003.zip › image data in the air/4.tif]

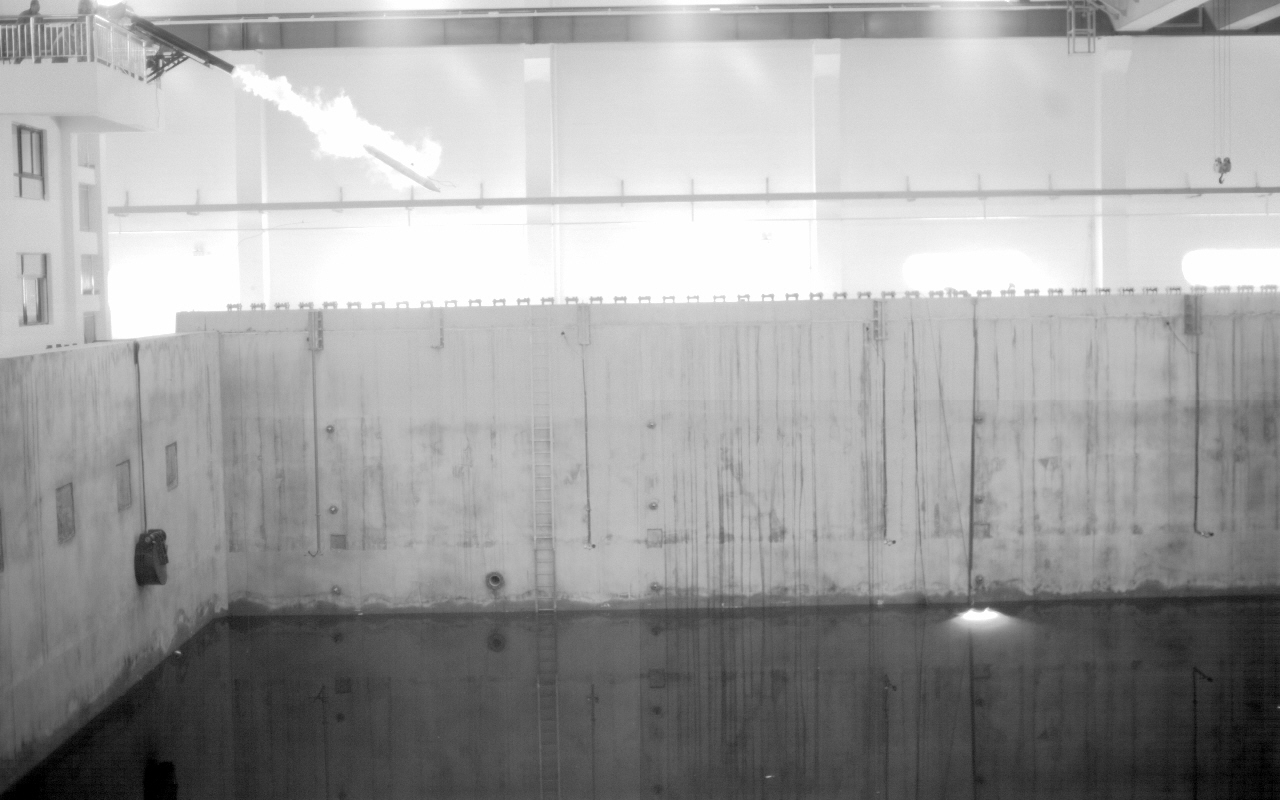

Supplement: S1 File — (ZIP) [file pone.0178461.s003.zip › image data in the air/5.tif]

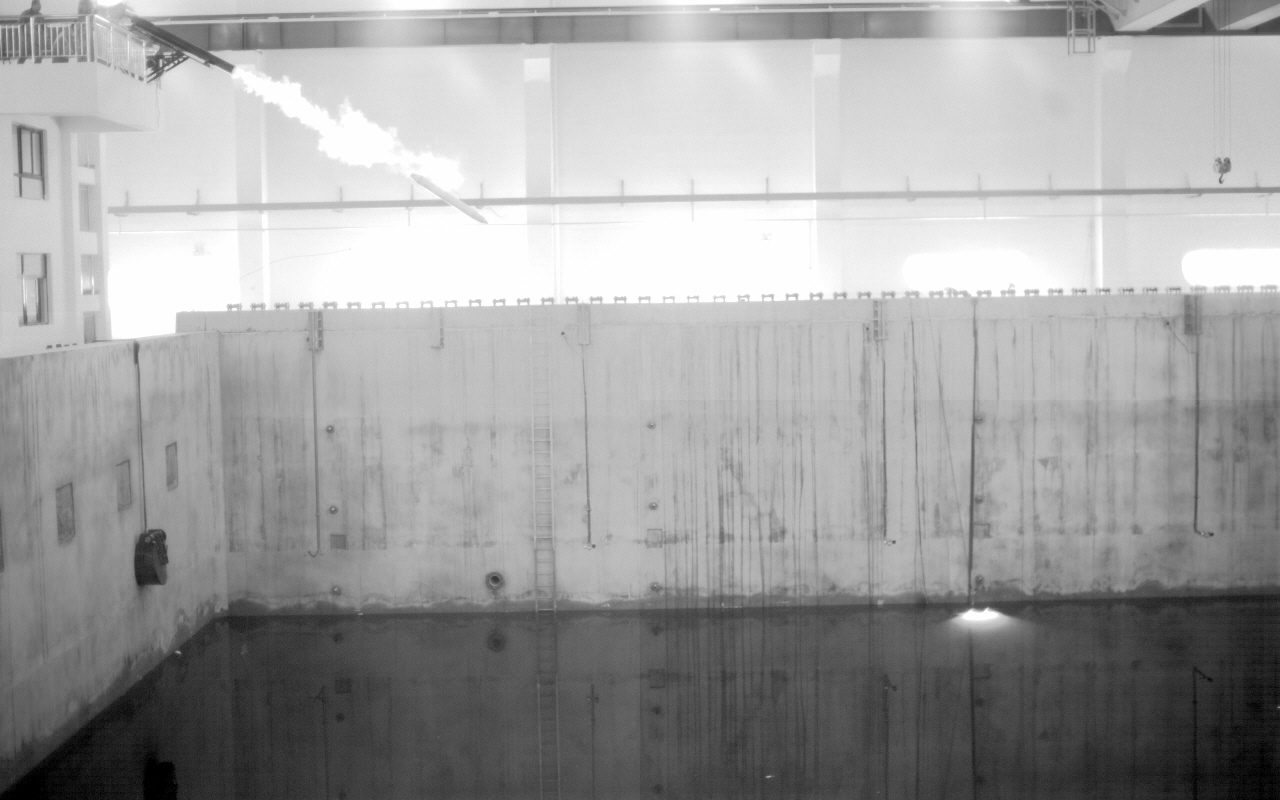

Supplement: S1 File — (ZIP) [file pone.0178461.s003.zip › image data in the air/6.tif]

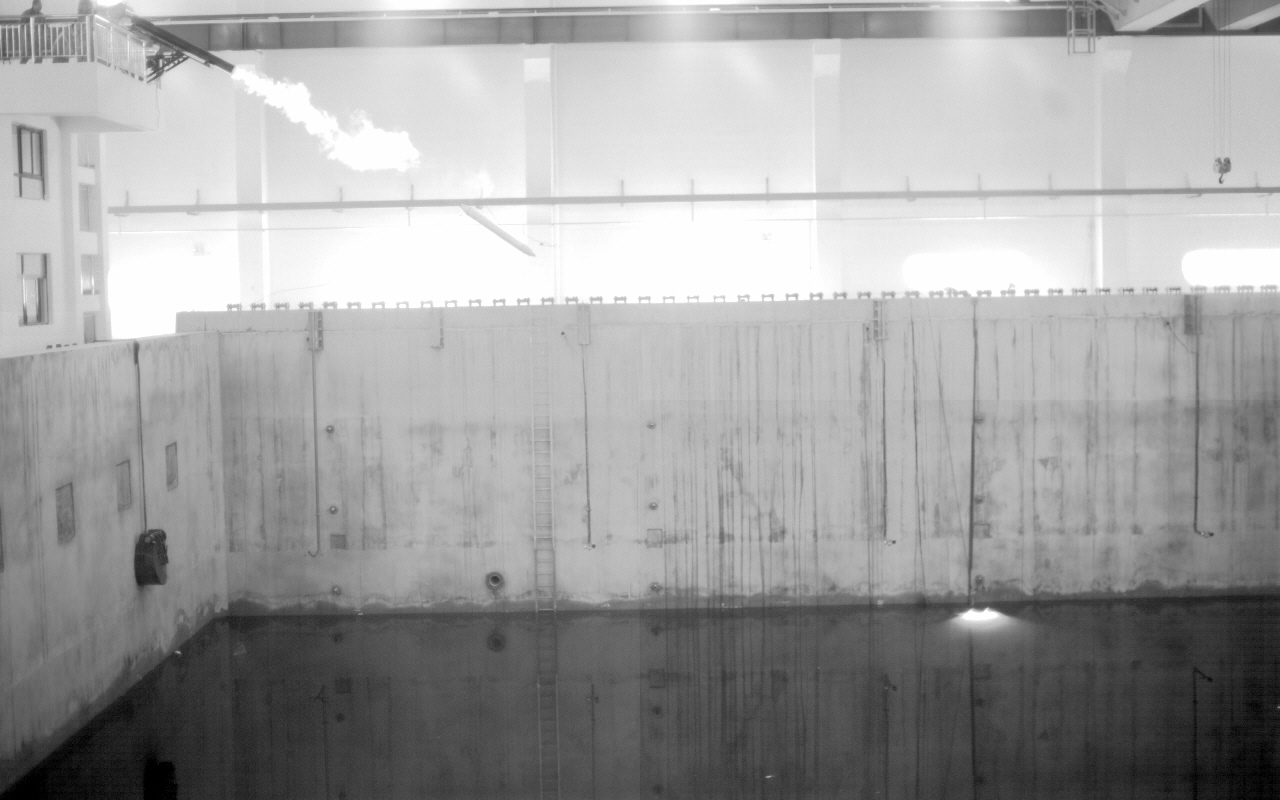

Supplement: S1 File — (ZIP) [file pone.0178461.s003.zip › image data in the air/7.tif]

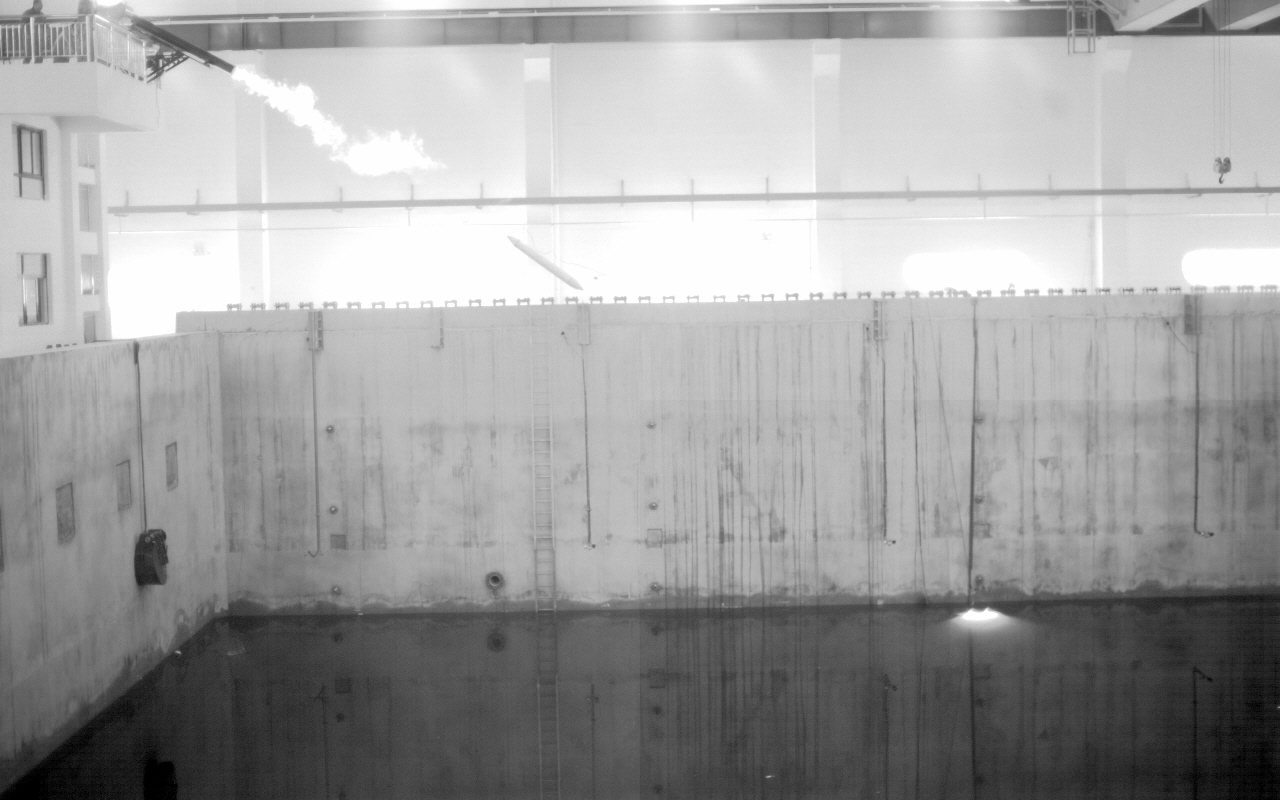

Supplement: S1 File — (ZIP) [file pone.0178461.s003.zip › image data in the air/8.tif]

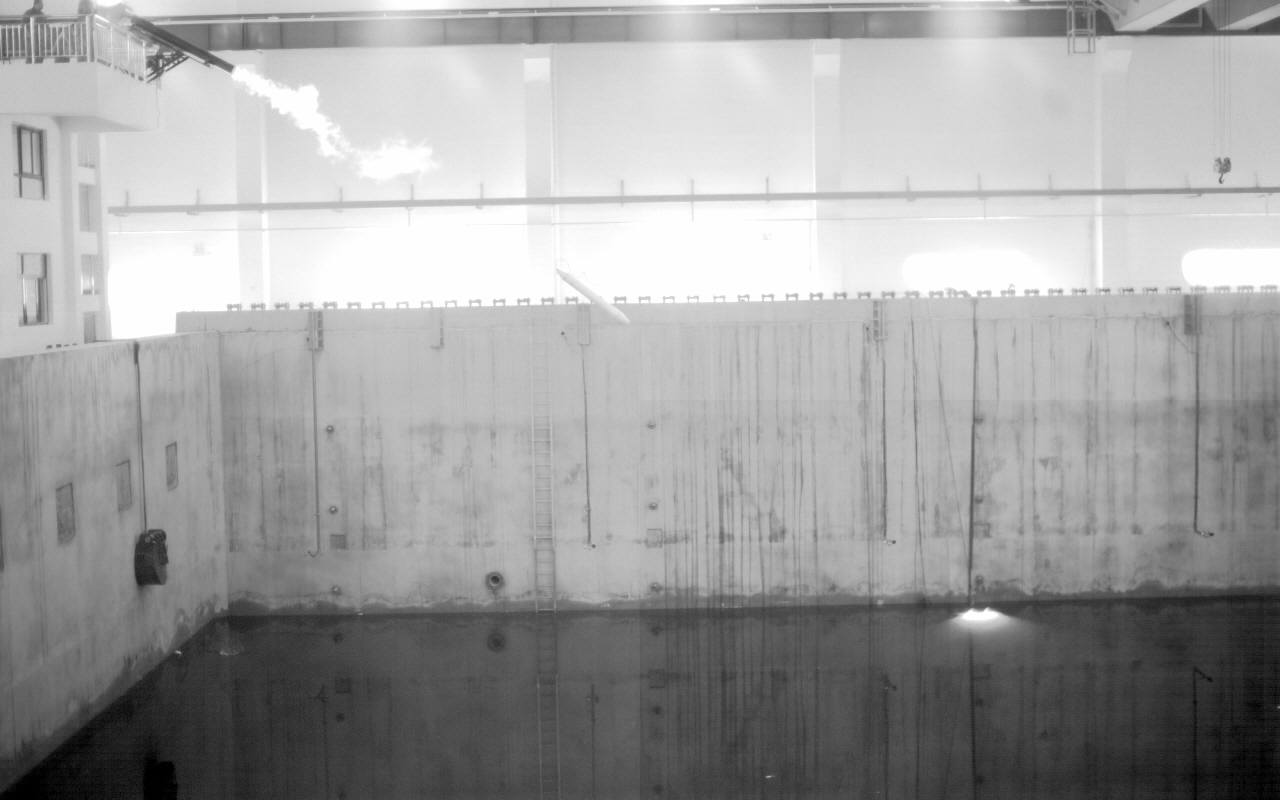

Supplement: S1 File — (ZIP) [file pone.0178461.s003.zip › image data in the air/9.tif]

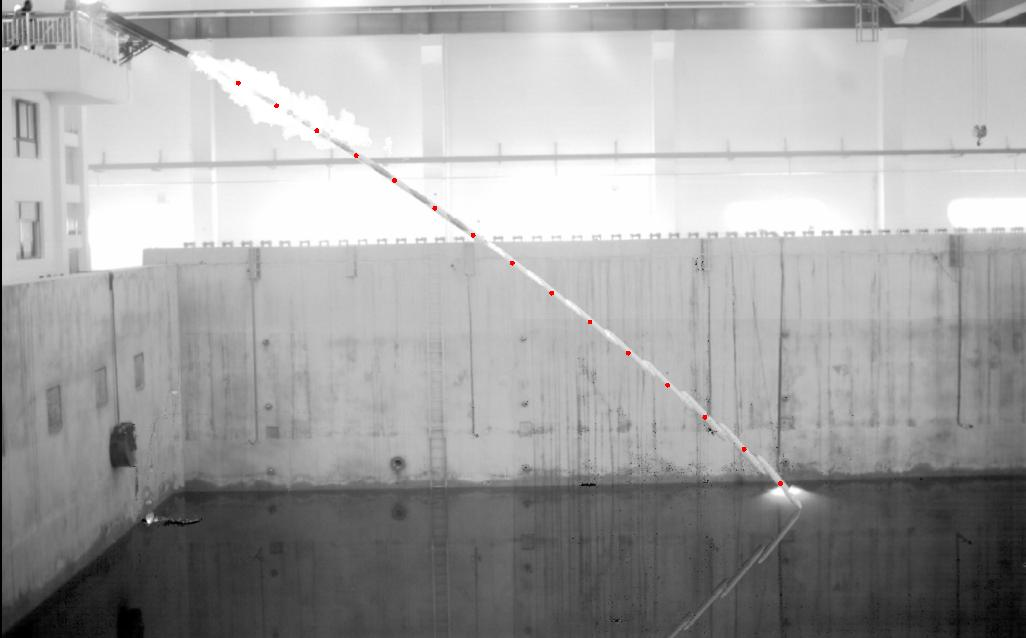

Supplement: S1 File — (ZIP) [file pone.0178461.s003.zip › image data in the air/the full trajectory in the air.tif]

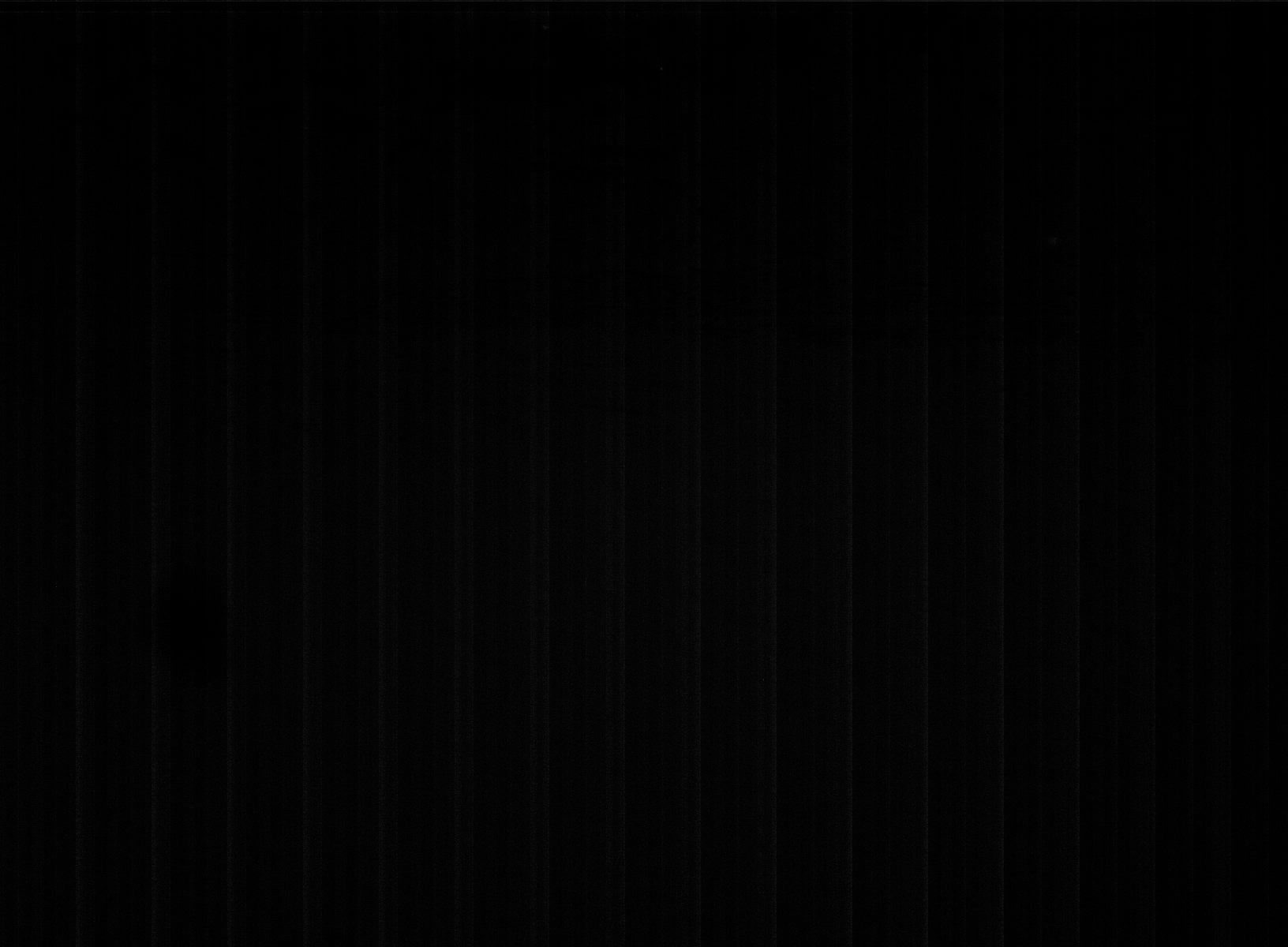

Supplement: S2 File — (ZIP) [file pone.0178461.s004.zip › underwater image data/1.tif]

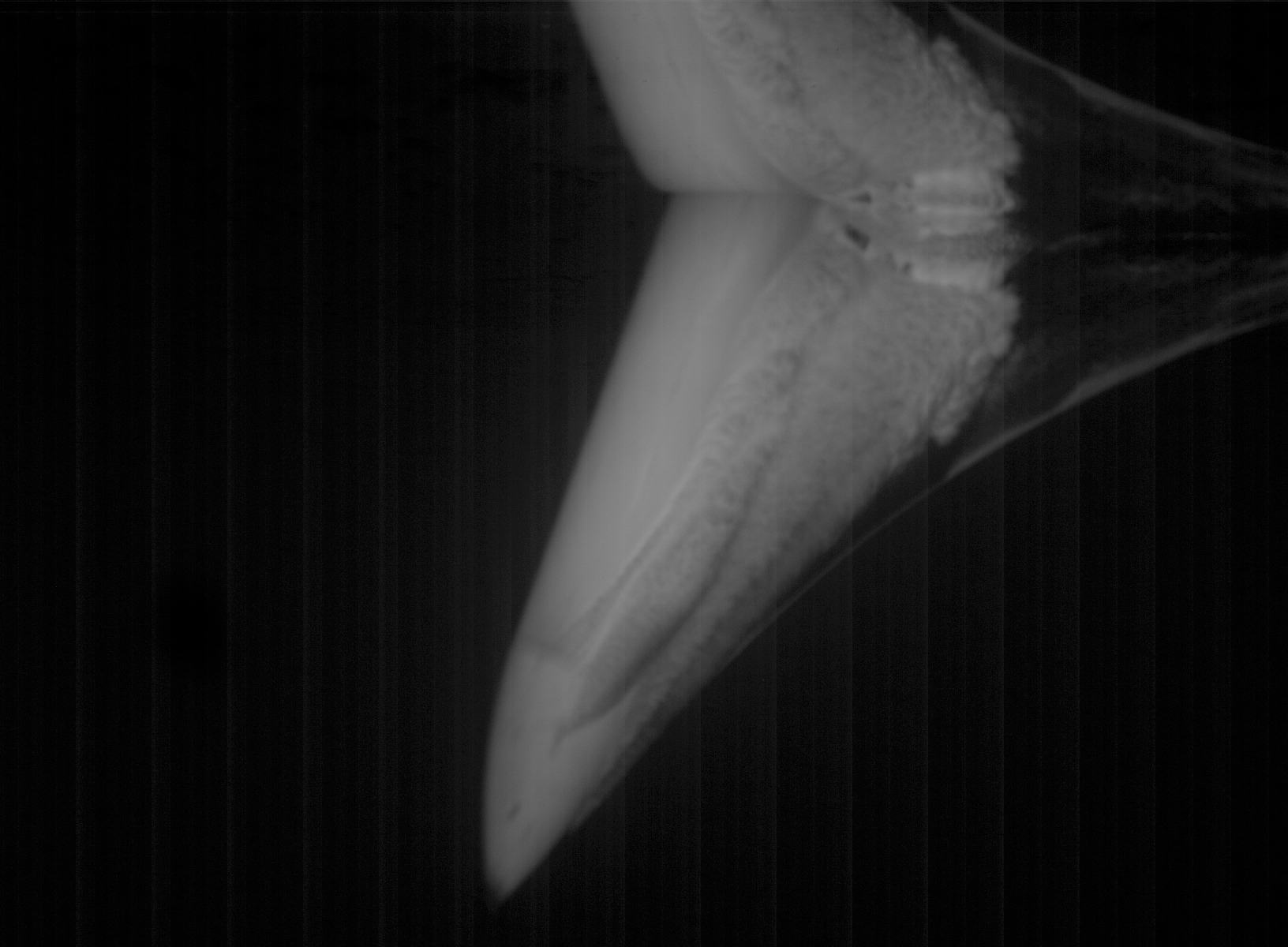

Supplement: S2 File — (ZIP) [file pone.0178461.s004.zip › underwater image data/10.tif]

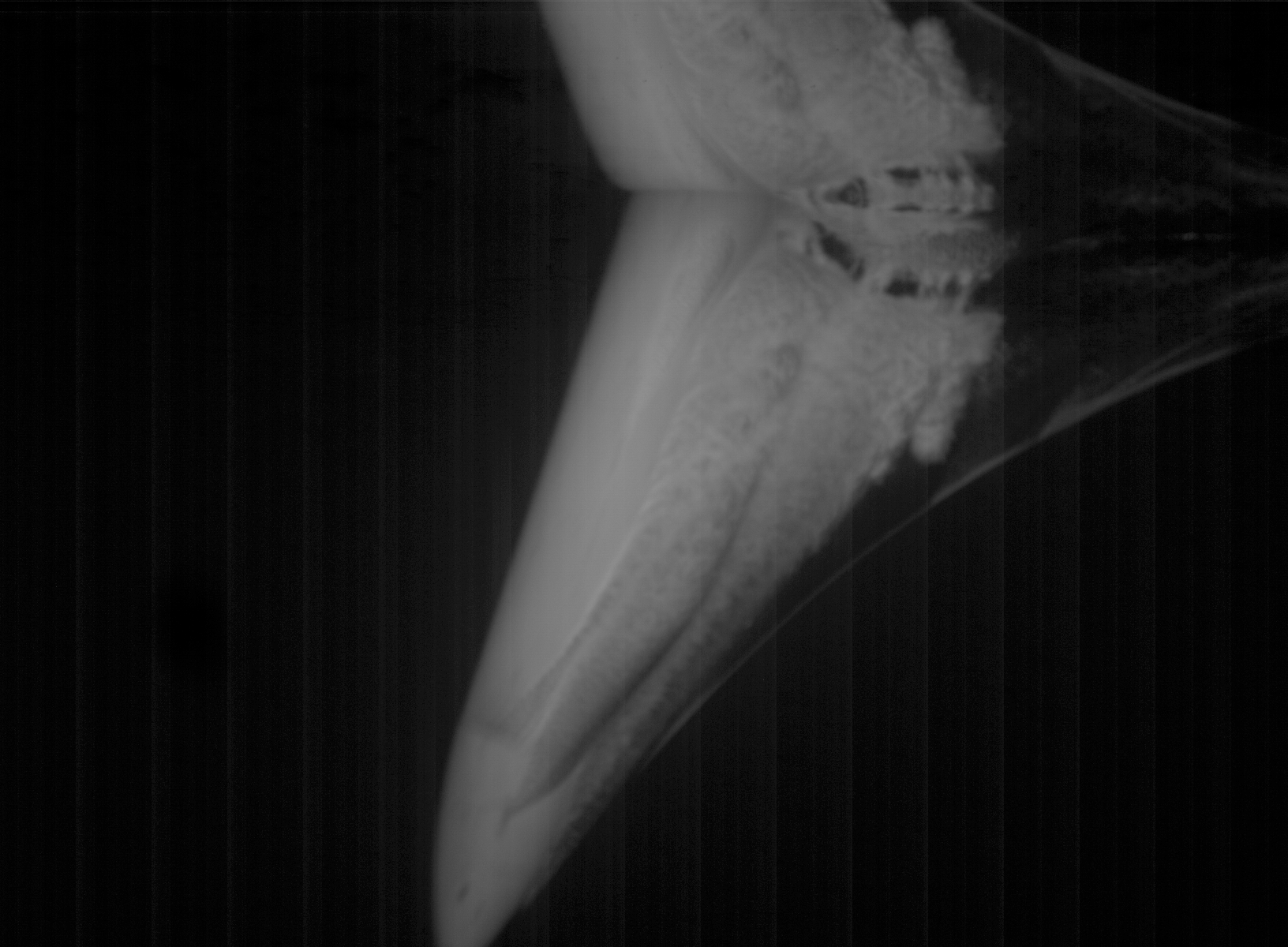

Supplement: S2 File — (ZIP) [file pone.0178461.s004.zip › underwater image data/11.tif]

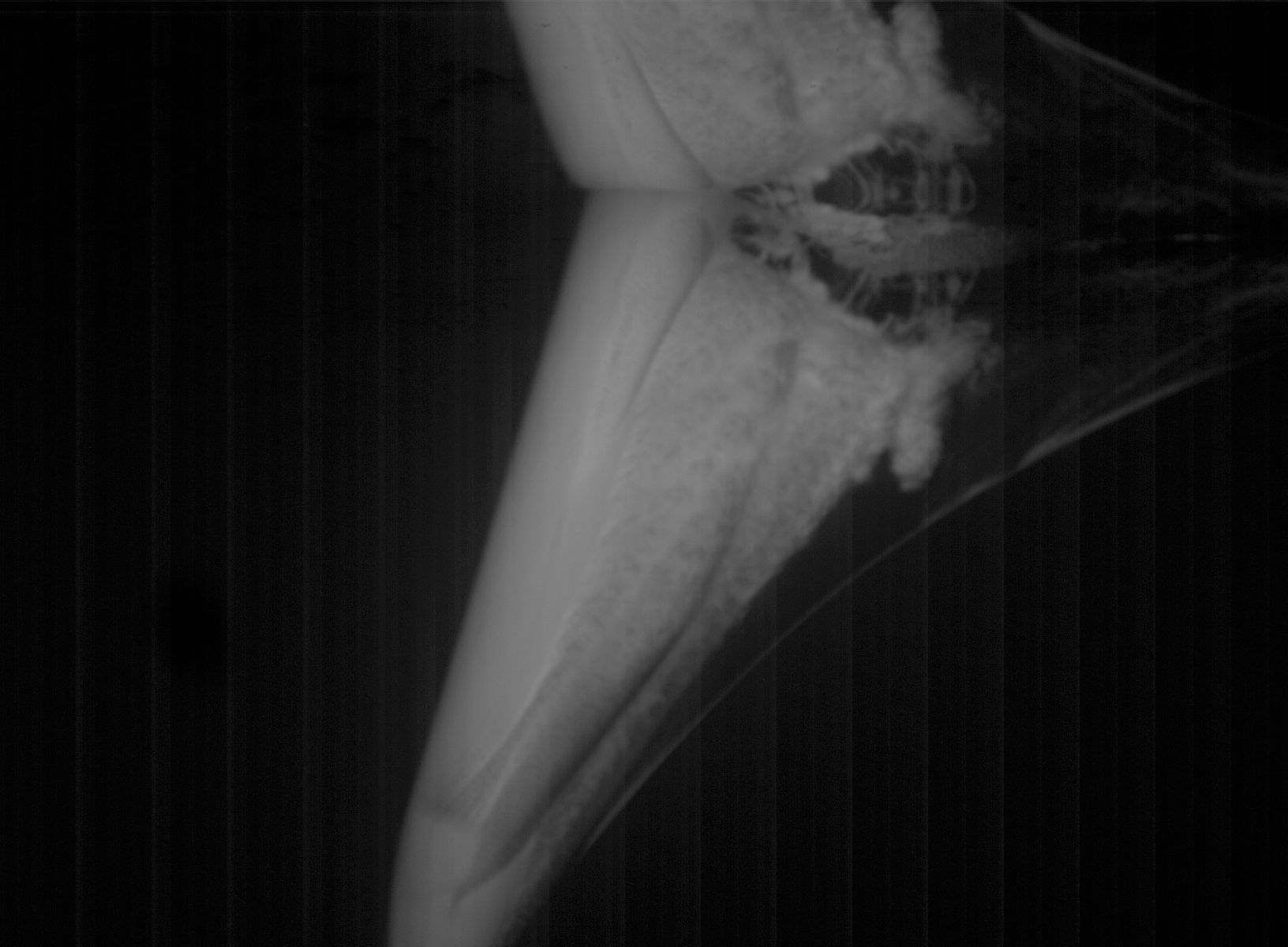

Supplement: S2 File — (ZIP) [file pone.0178461.s004.zip › underwater image data/12.tif]

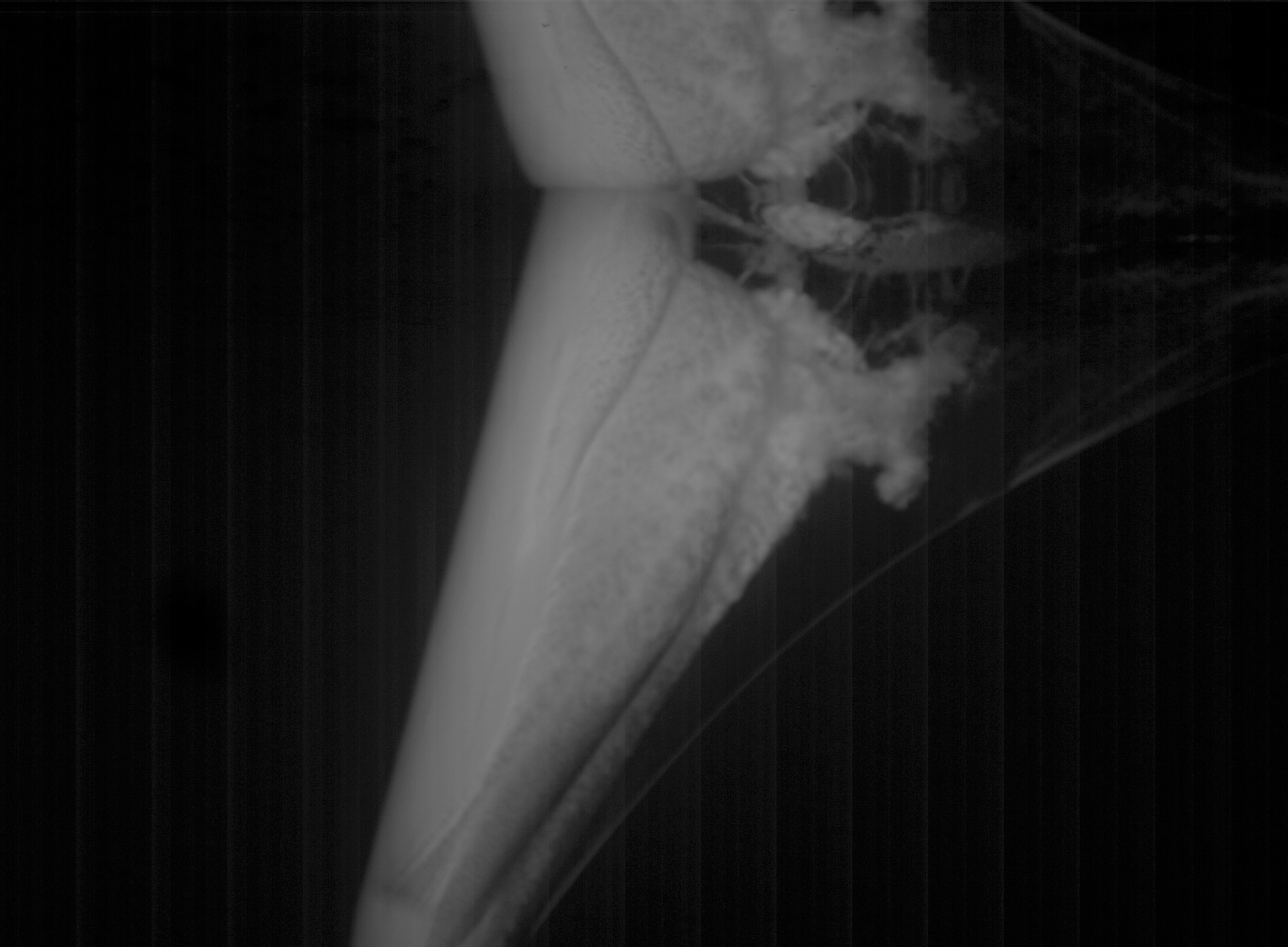

Supplement: S2 File — (ZIP) [file pone.0178461.s004.zip › underwater image data/13.tif]

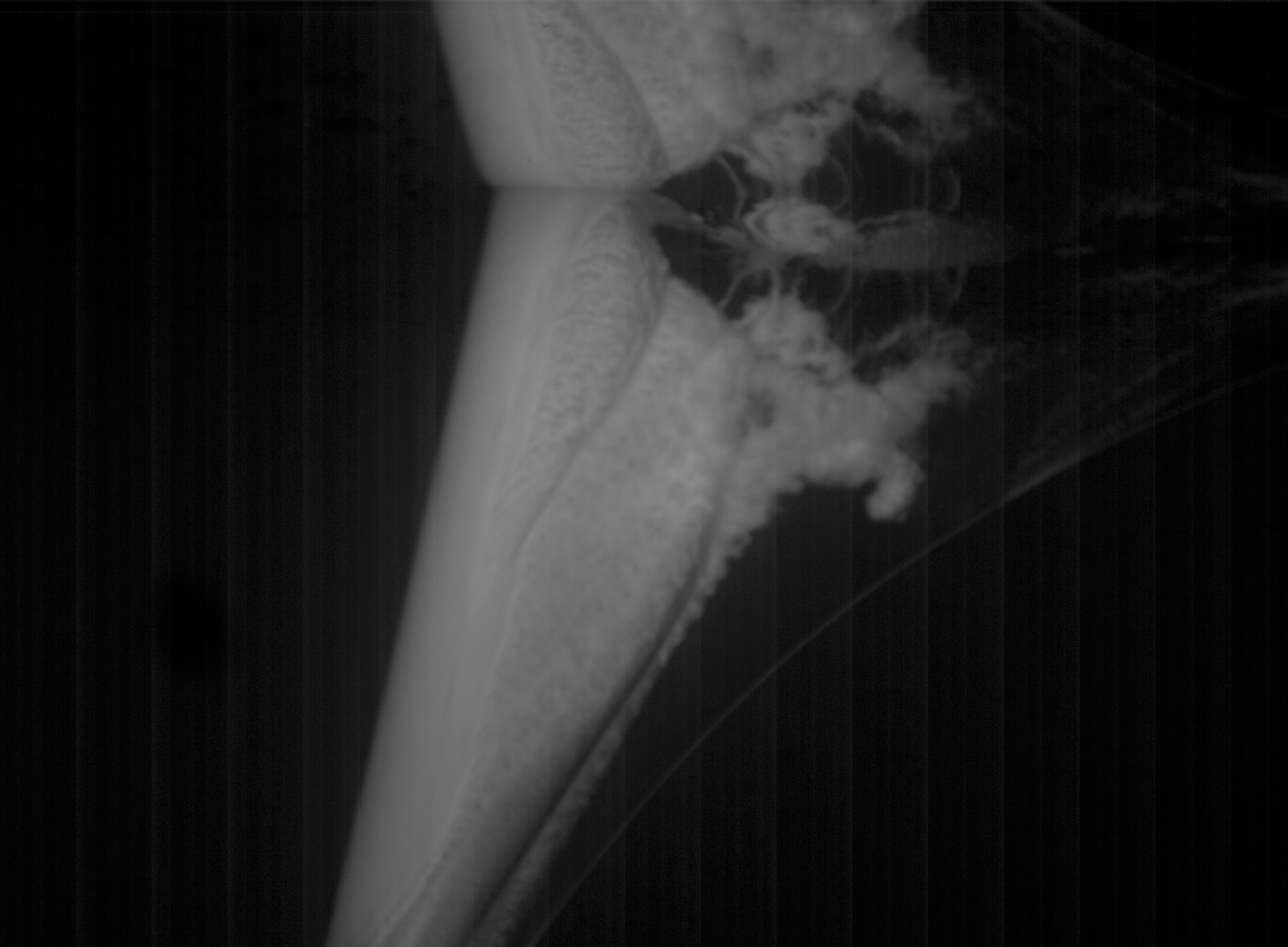

Supplement: S2 File — (ZIP) [file pone.0178461.s004.zip › underwater image data/14.tif]

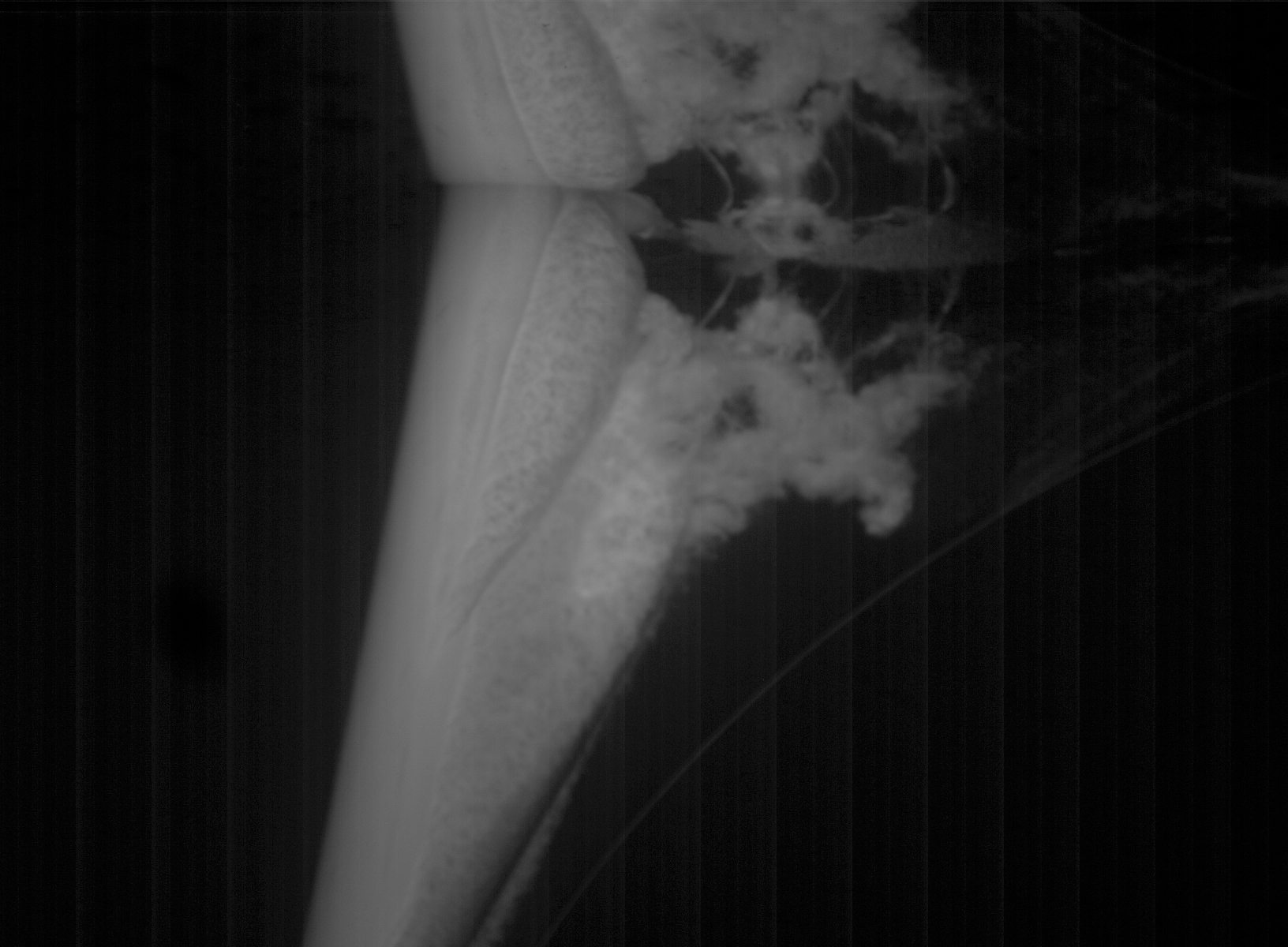

Supplement: S2 File — (ZIP) [file pone.0178461.s004.zip › underwater image data/15.tif]

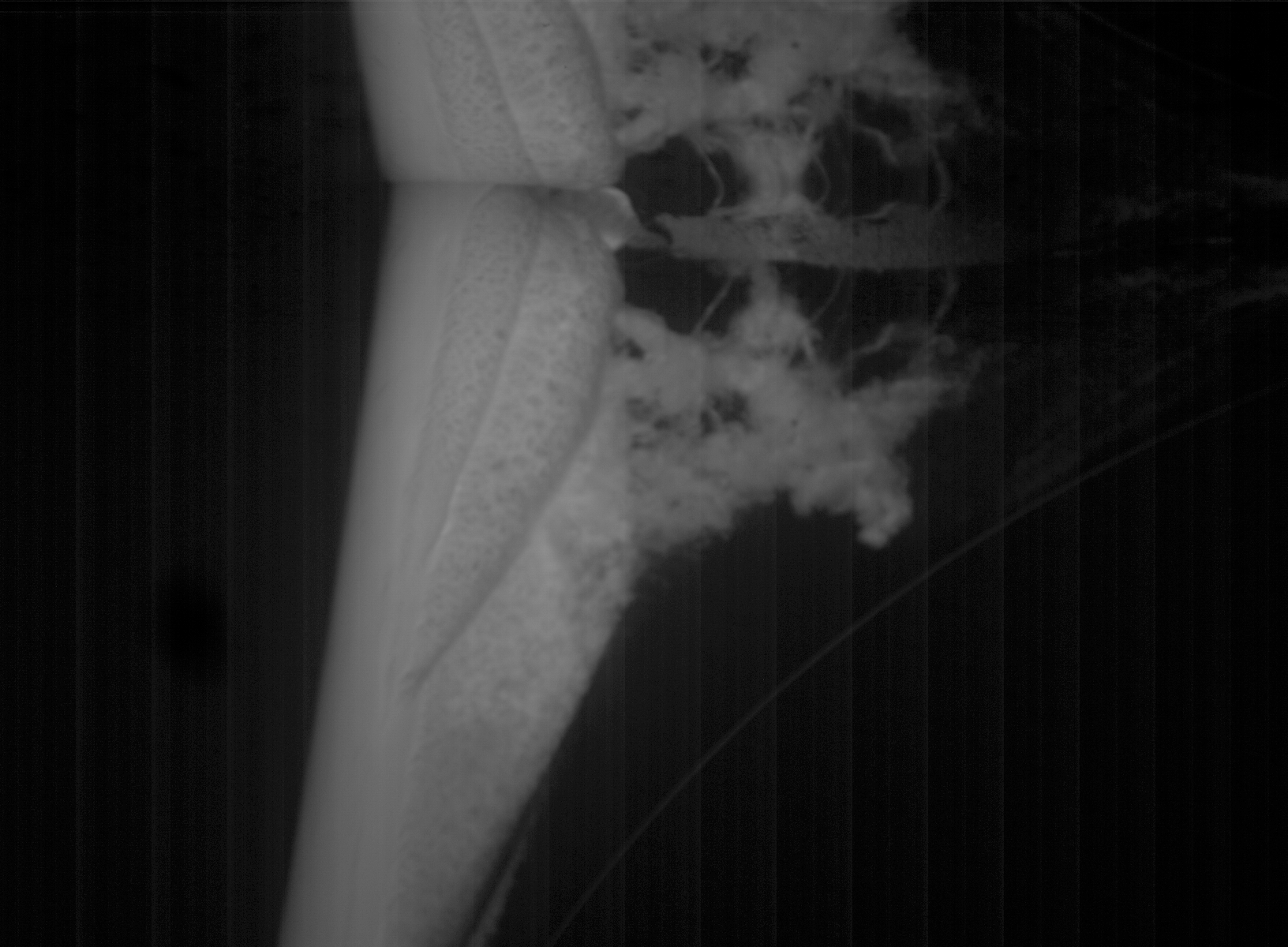

Supplement: S2 File — (ZIP) [file pone.0178461.s004.zip › underwater image data/16.tif]

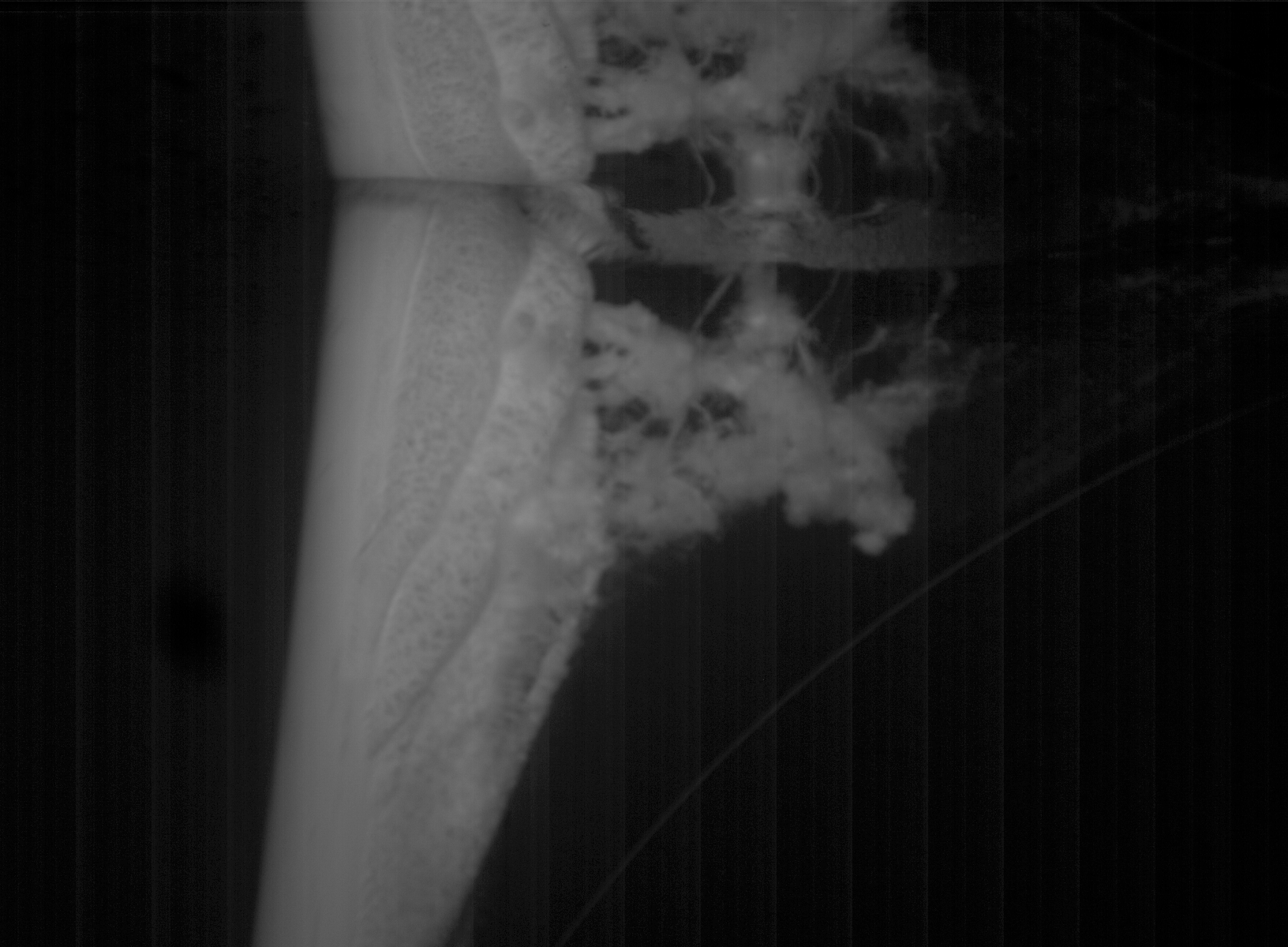

Supplement: S2 File — (ZIP) [file pone.0178461.s004.zip › underwater image data/17.tif]

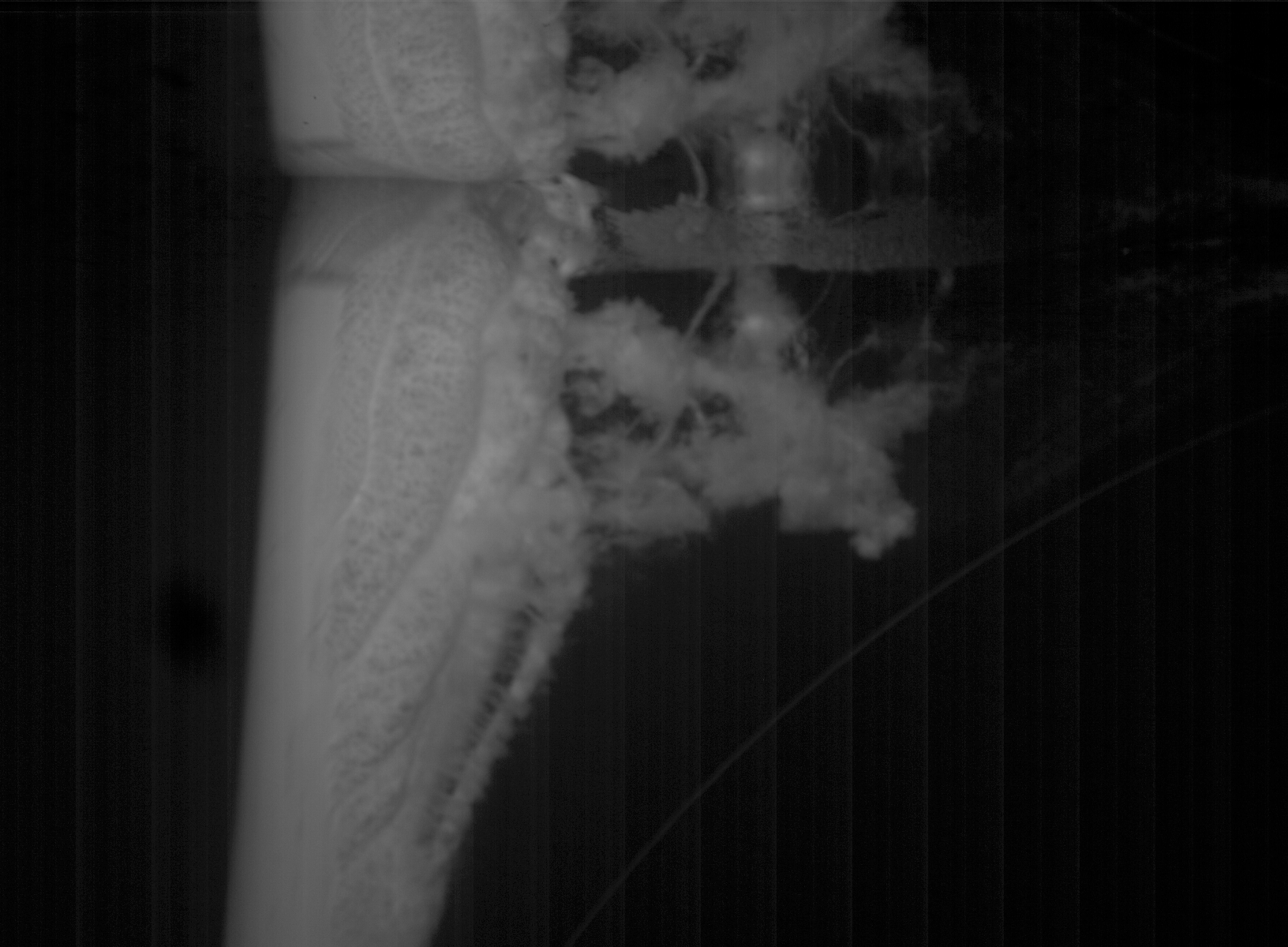

Supplement: S2 File — (ZIP) [file pone.0178461.s004.zip › underwater image data/18.tif]

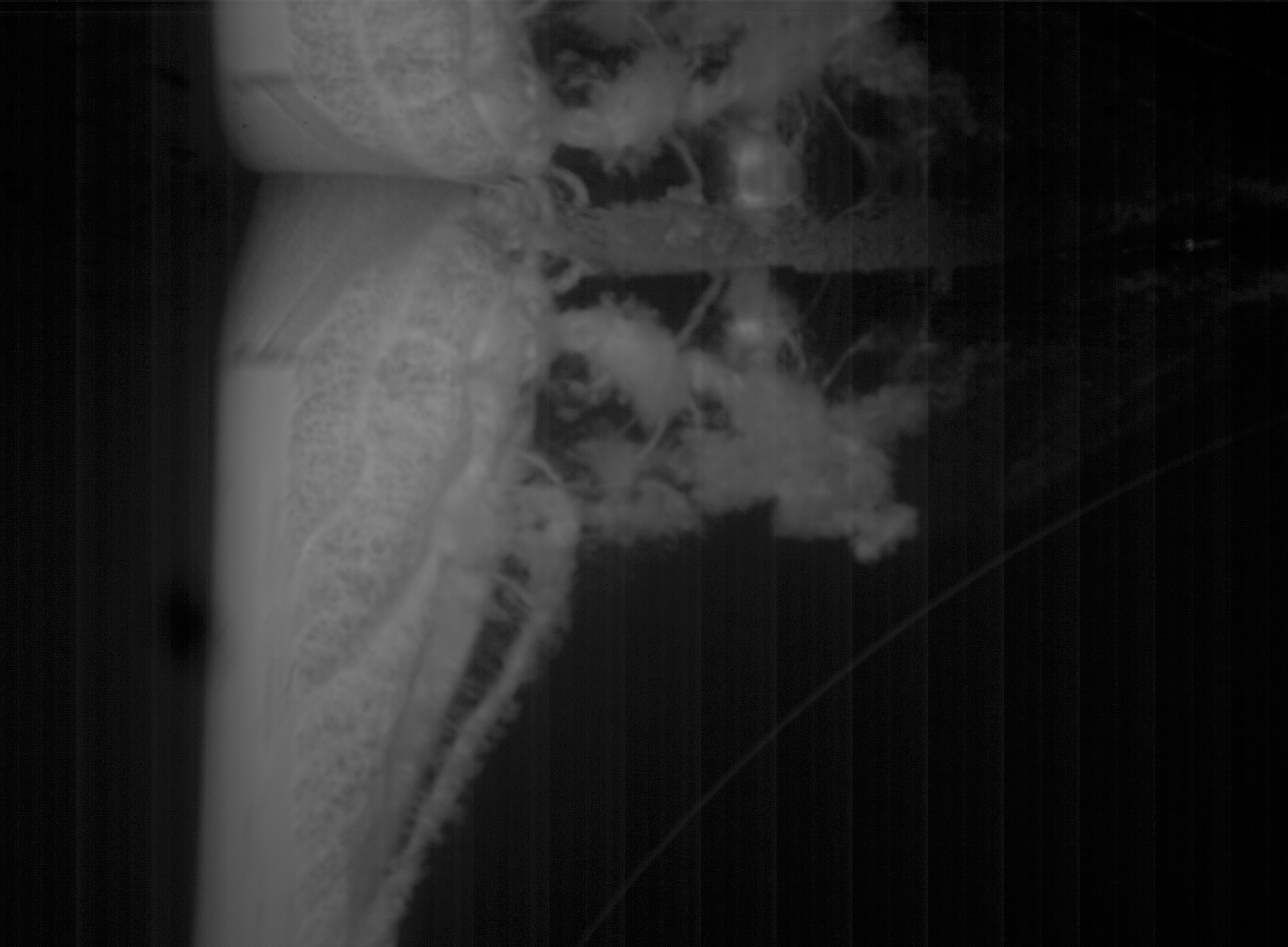

Supplement: S2 File — (ZIP) [file pone.0178461.s004.zip › underwater image data/19.tif]

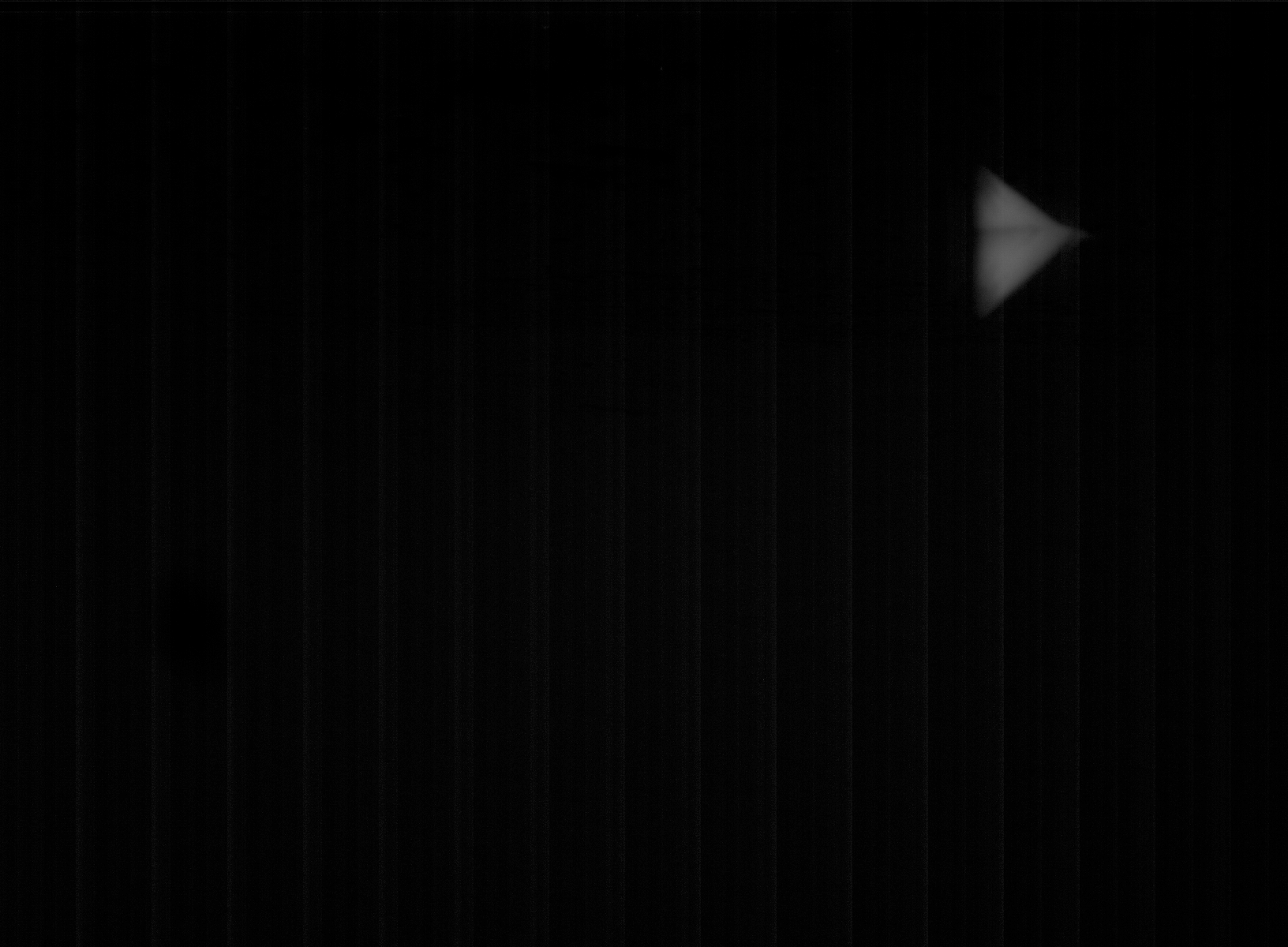

Supplement: S2 File — (ZIP) [file pone.0178461.s004.zip › underwater image data/2.tif]

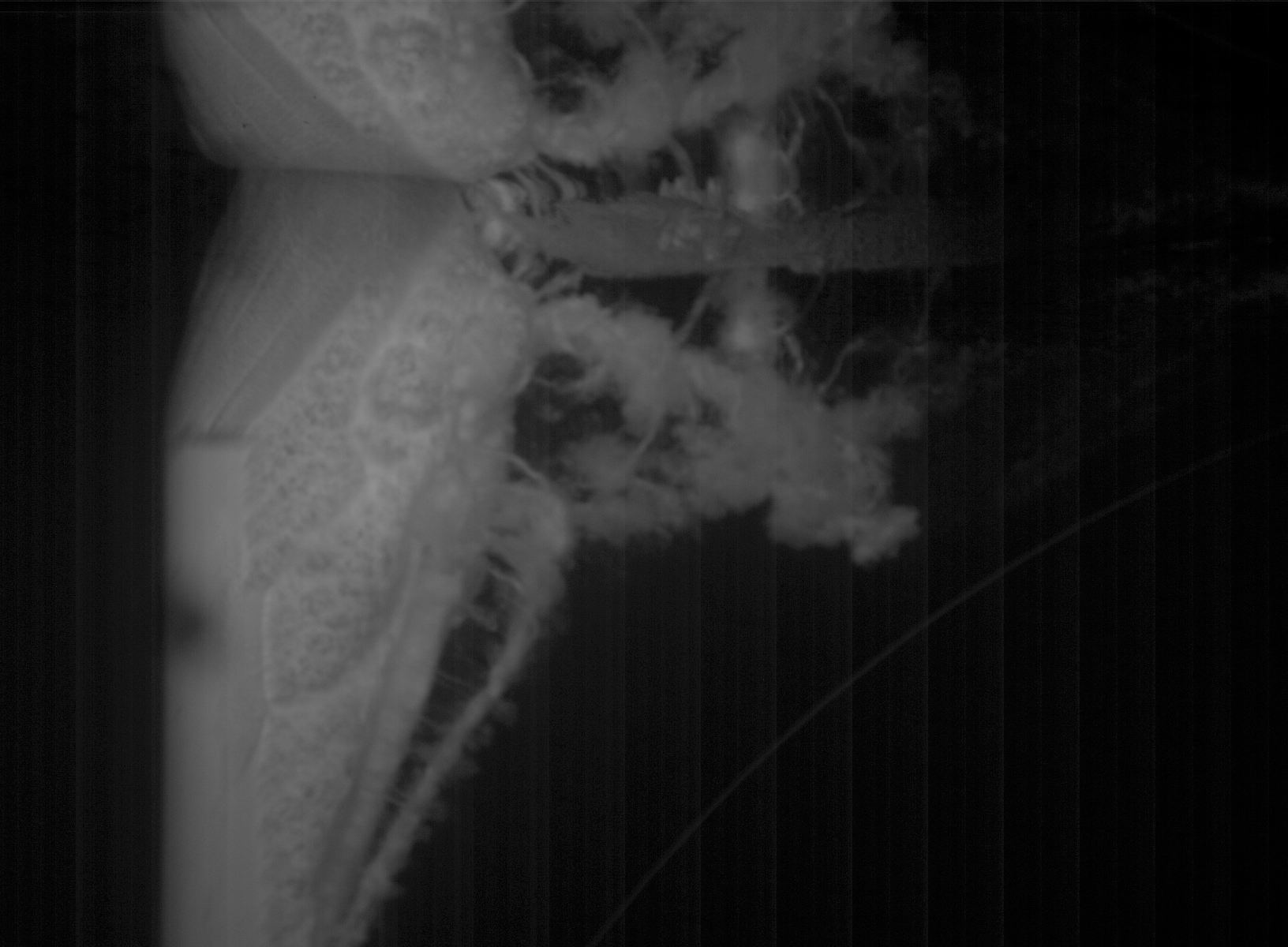

Supplement: S2 File — (ZIP) [file pone.0178461.s004.zip › underwater image data/20.tif]

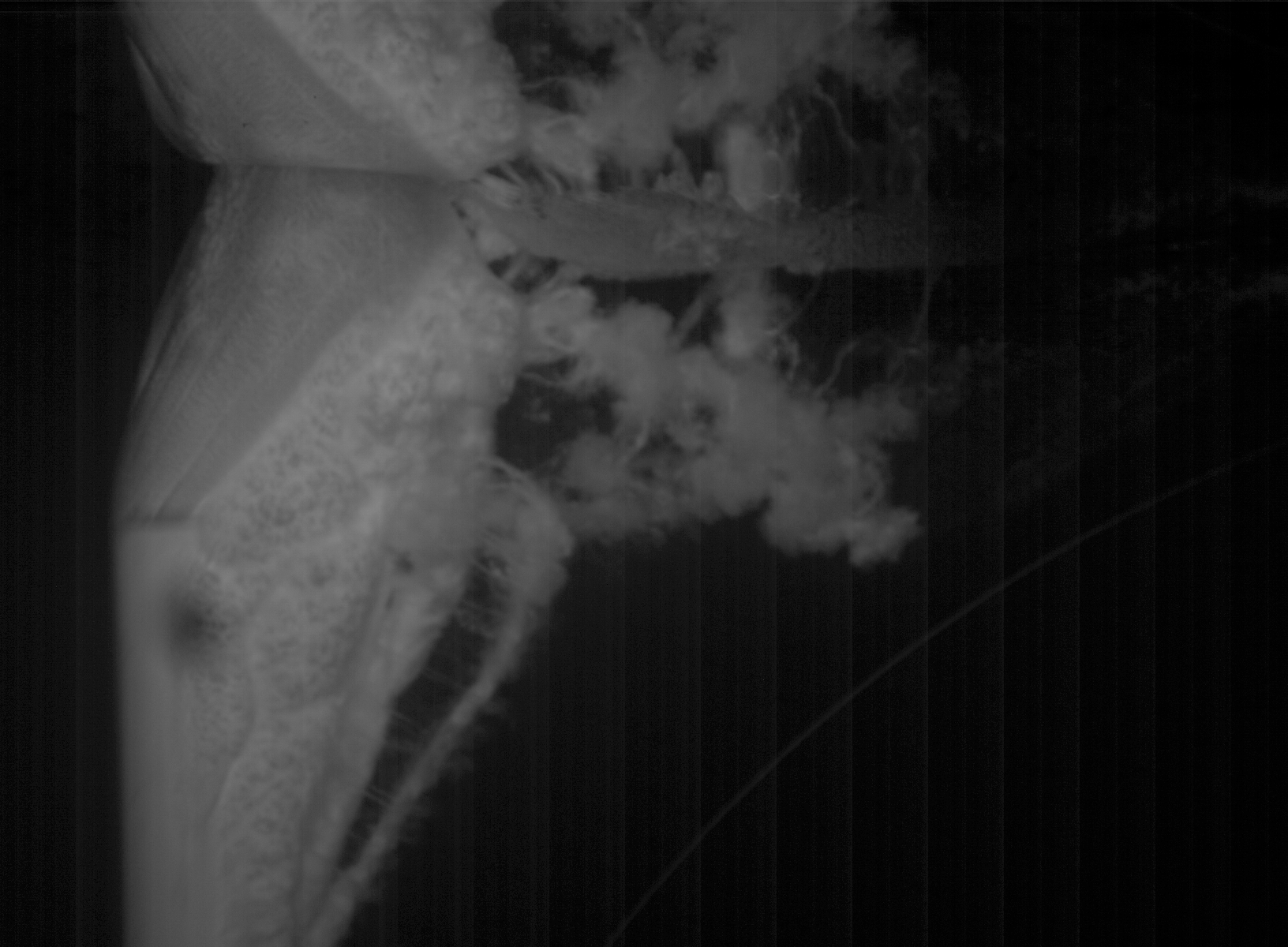

Supplement: S2 File — (ZIP) [file pone.0178461.s004.zip › underwater image data/21.tif]

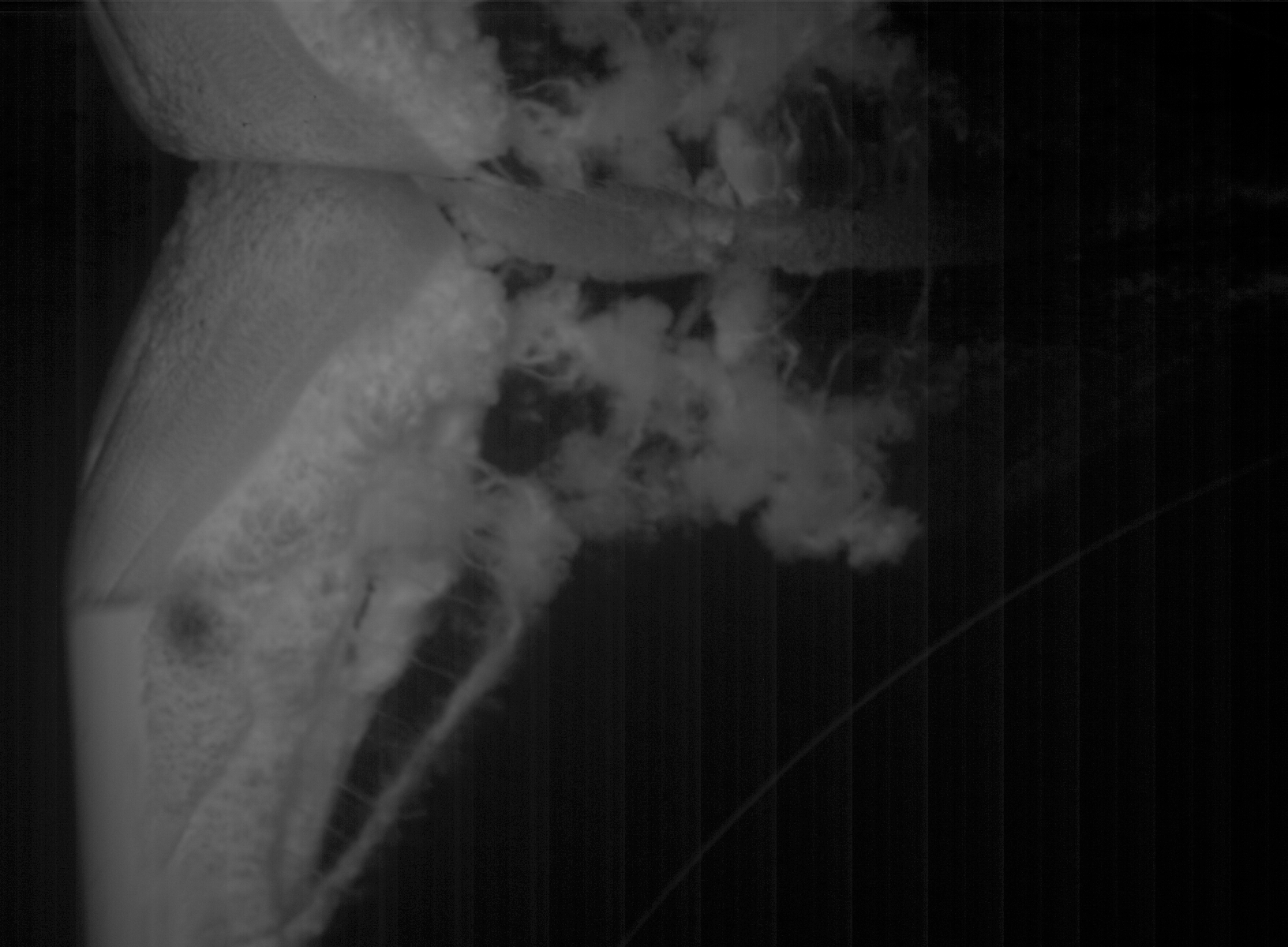

Supplement: S2 File — (ZIP) [file pone.0178461.s004.zip › underwater image data/22.tif]

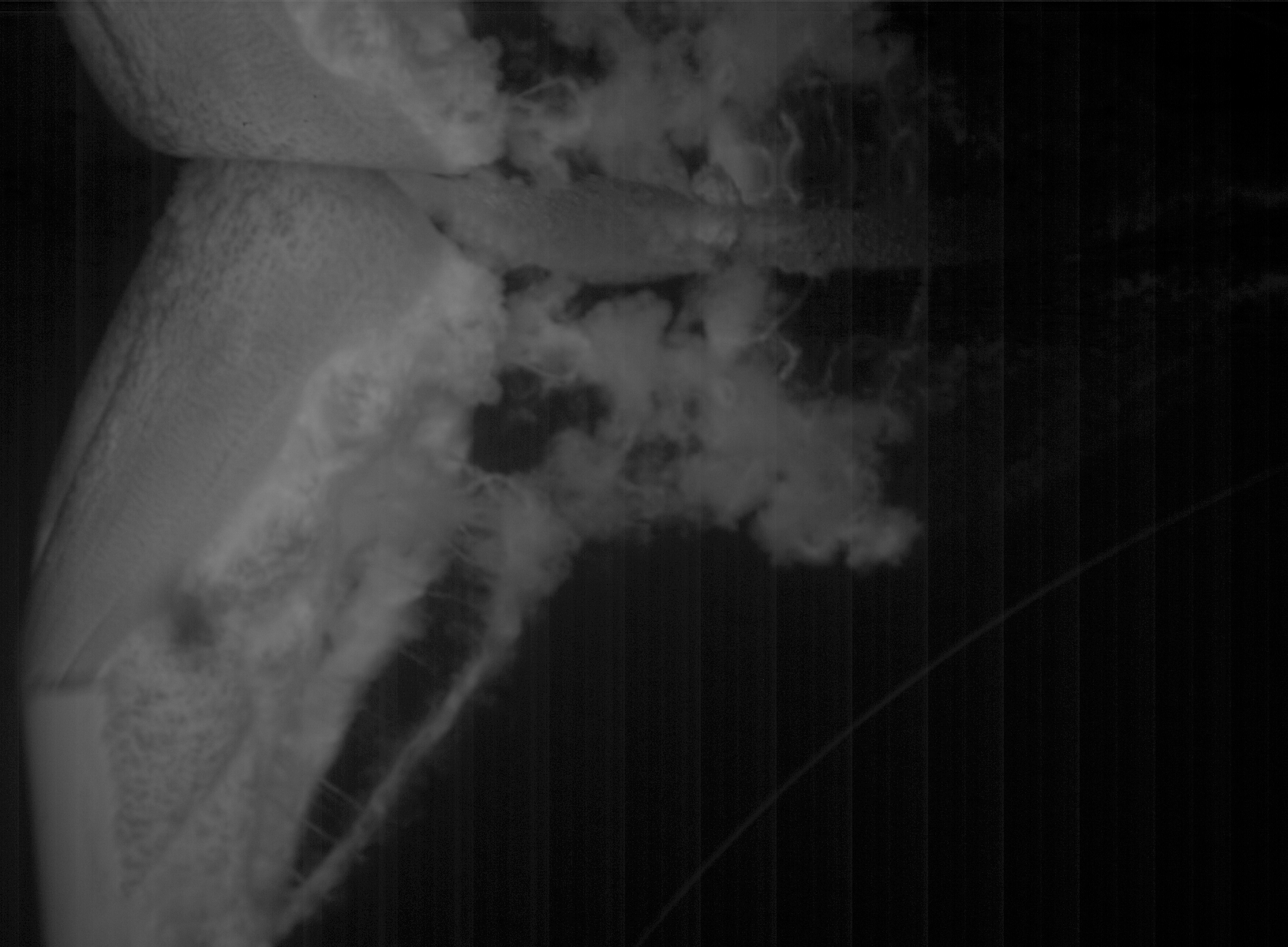

Supplement: S2 File — (ZIP) [file pone.0178461.s004.zip › underwater image data/23.tif]

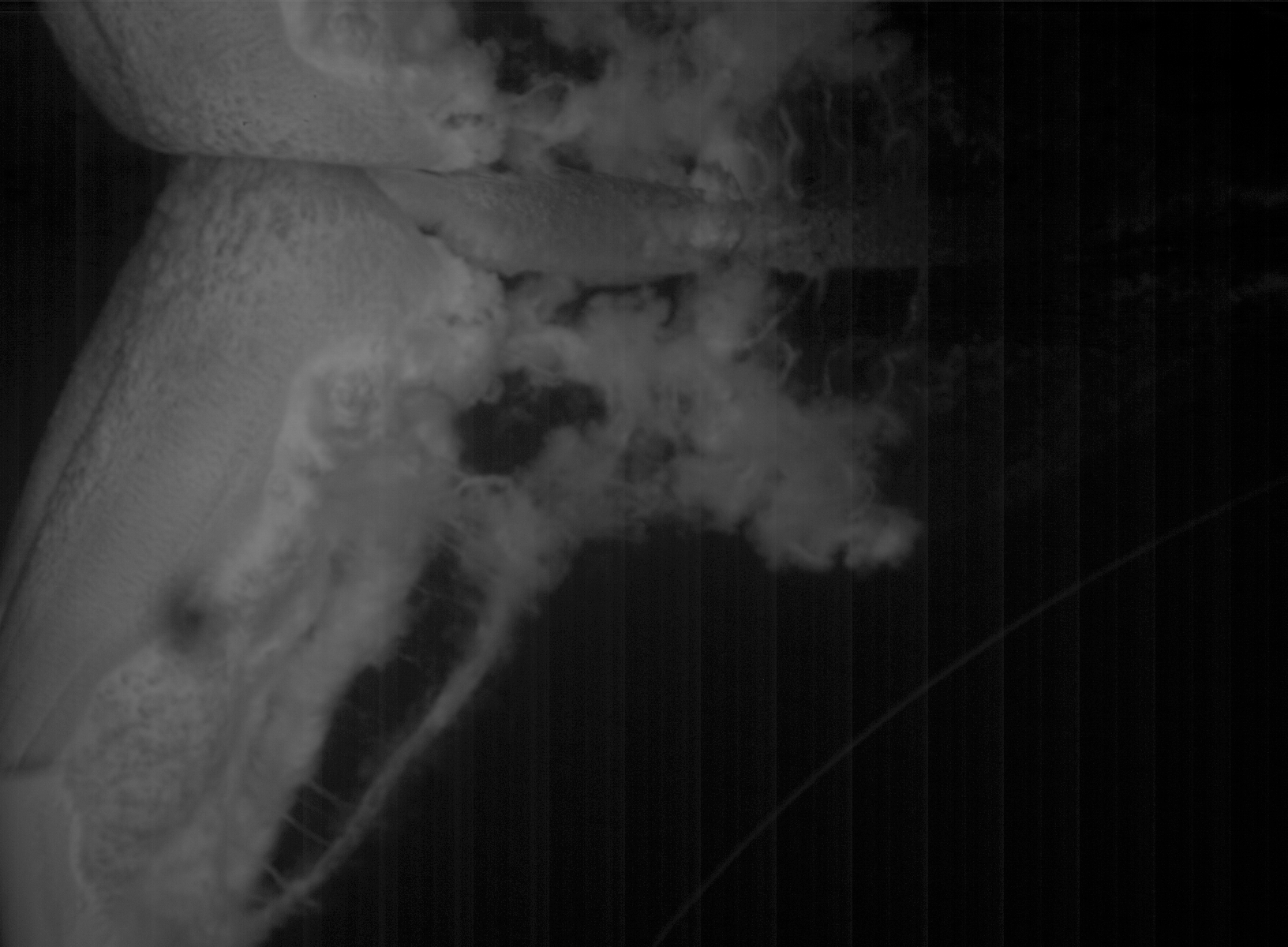

Supplement: S2 File — (ZIP) [file pone.0178461.s004.zip › underwater image data/24.tif]

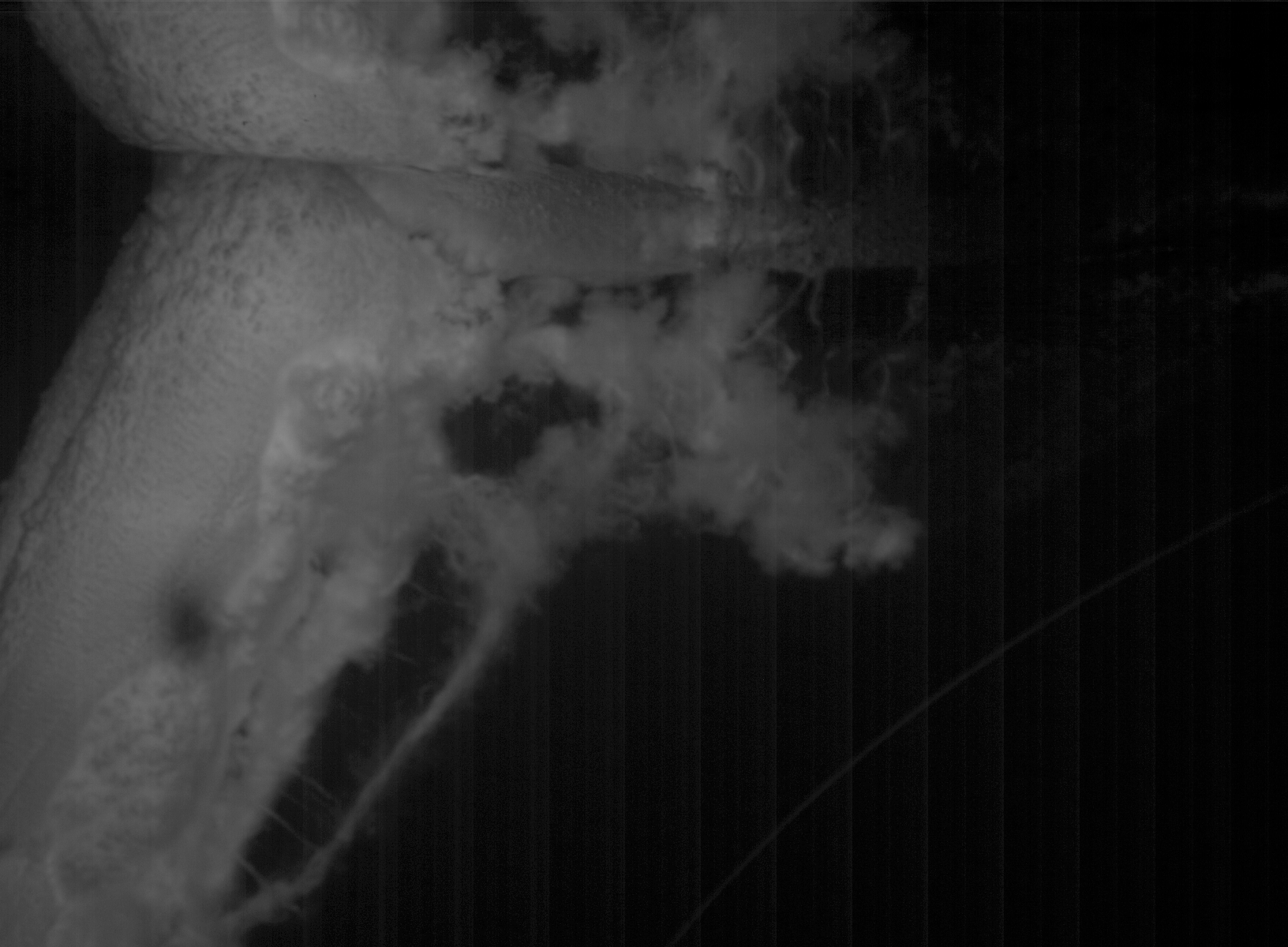

Supplement: S2 File — (ZIP) [file pone.0178461.s004.zip › underwater image data/25.tif]

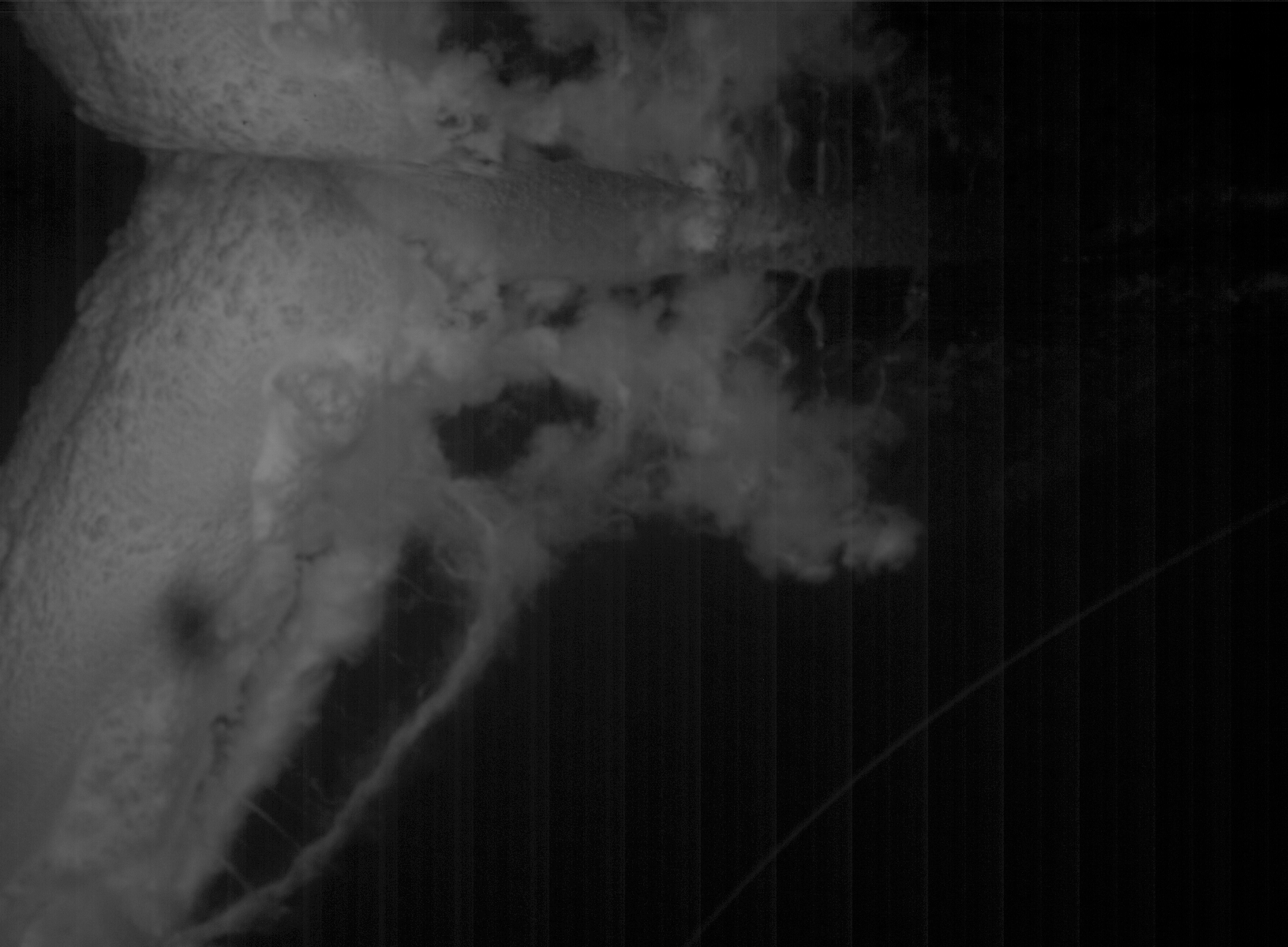

Supplement: S2 File — (ZIP) [file pone.0178461.s004.zip › underwater image data/26.tif]

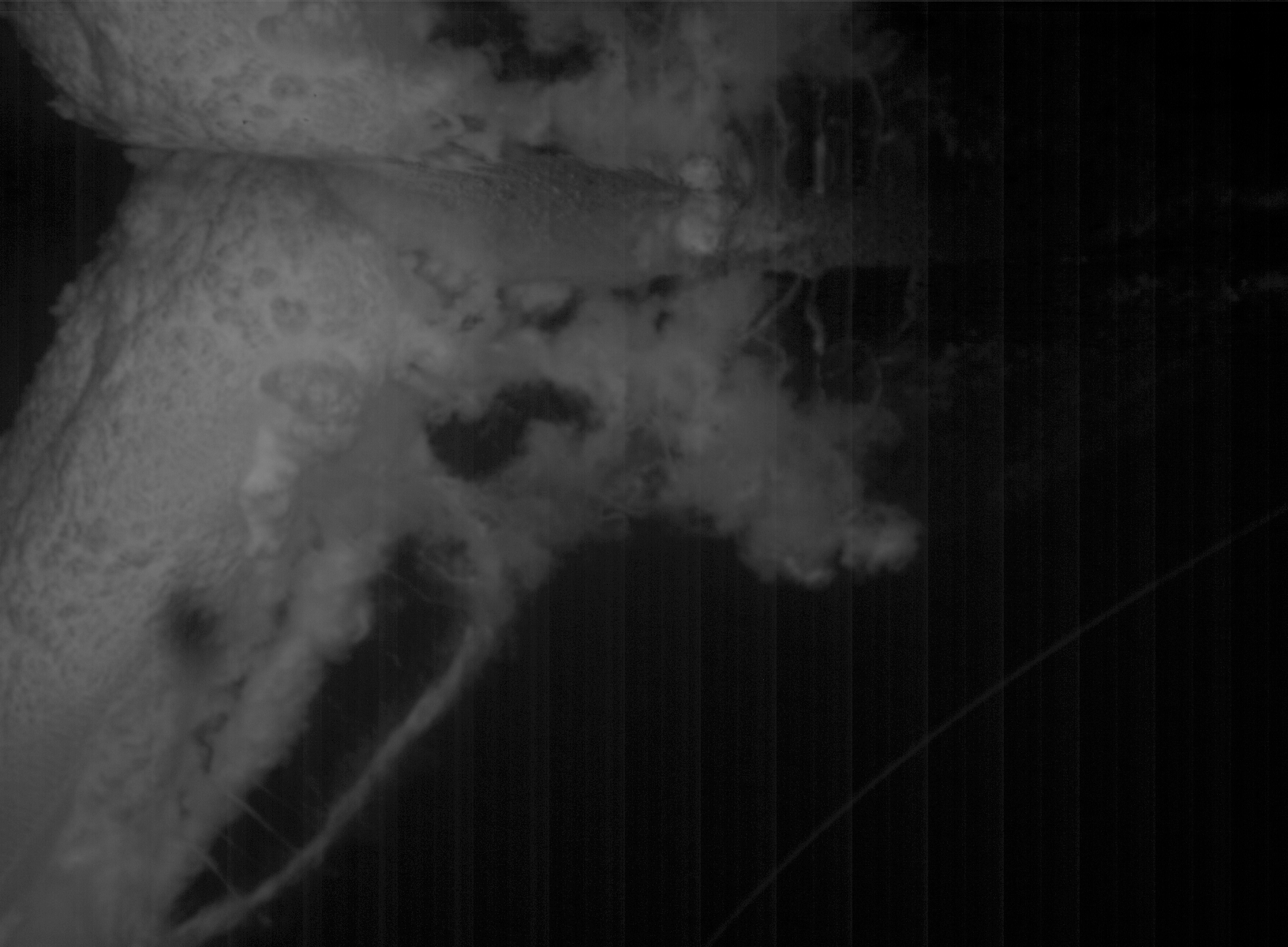

Supplement: S2 File — (ZIP) [file pone.0178461.s004.zip › underwater image data/27.tif]

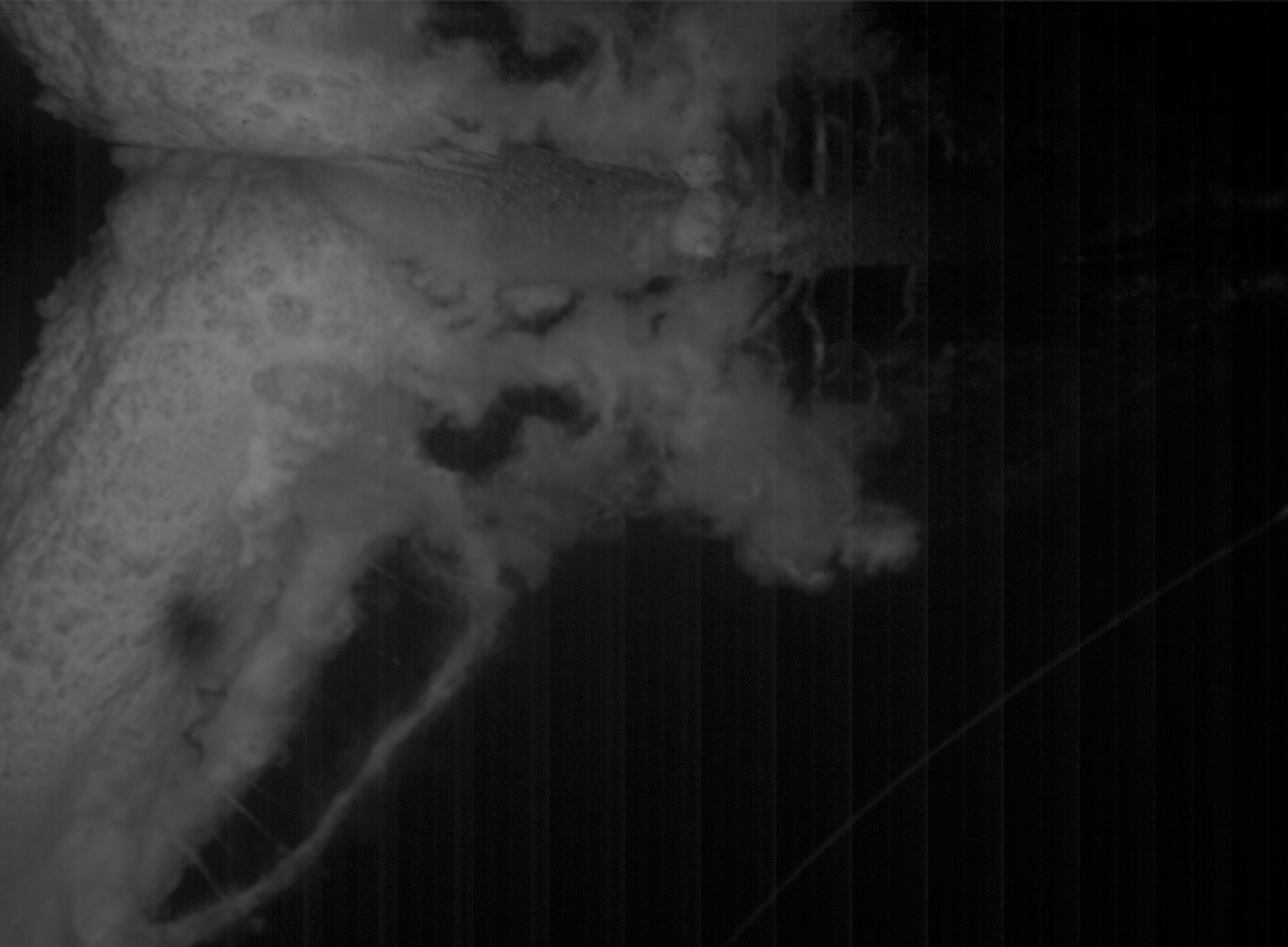

Supplement: S2 File — (ZIP) [file pone.0178461.s004.zip › underwater image data/28.tif]

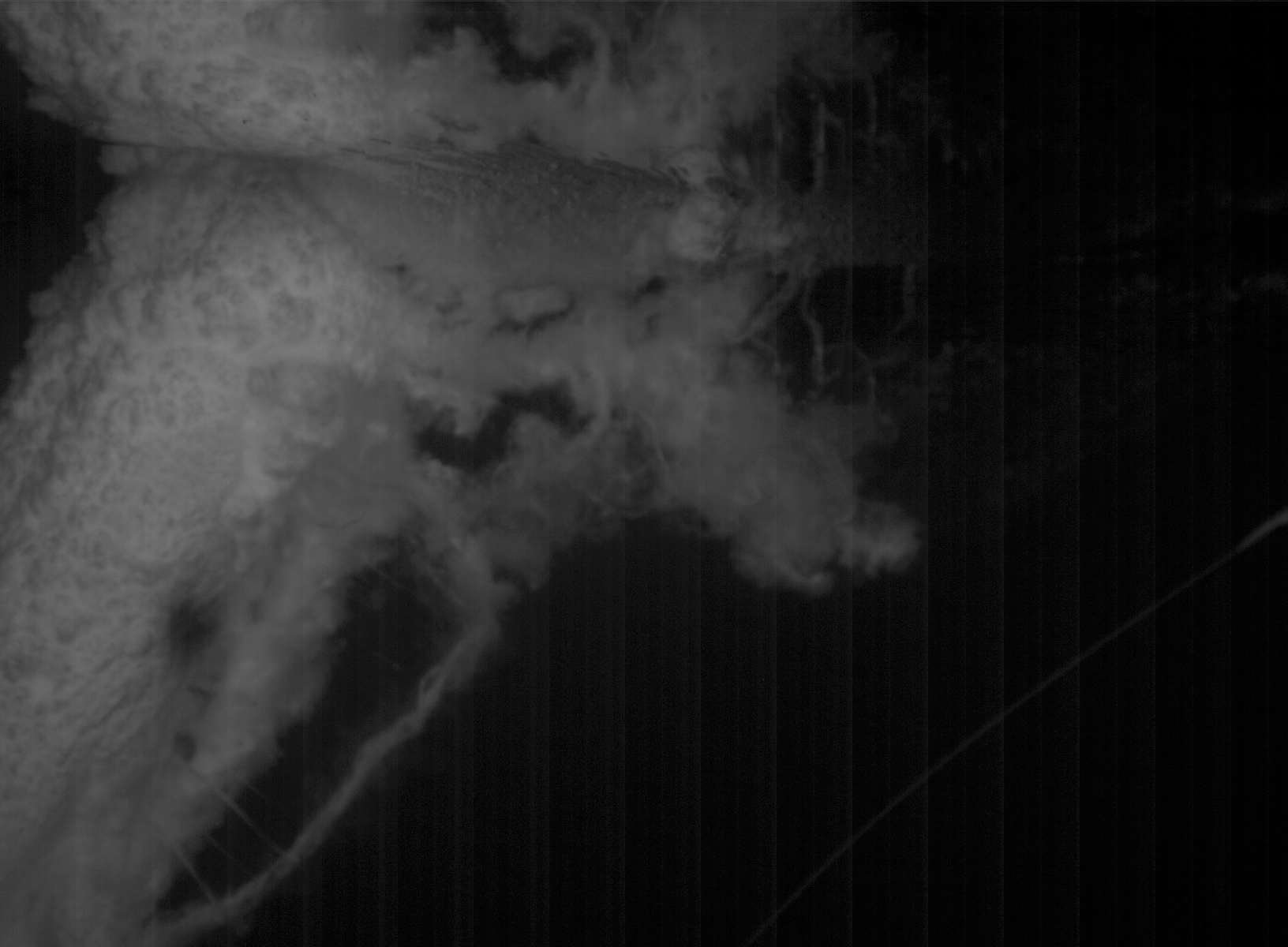

Supplement: S2 File — (ZIP) [file pone.0178461.s004.zip › underwater image data/29.tif]

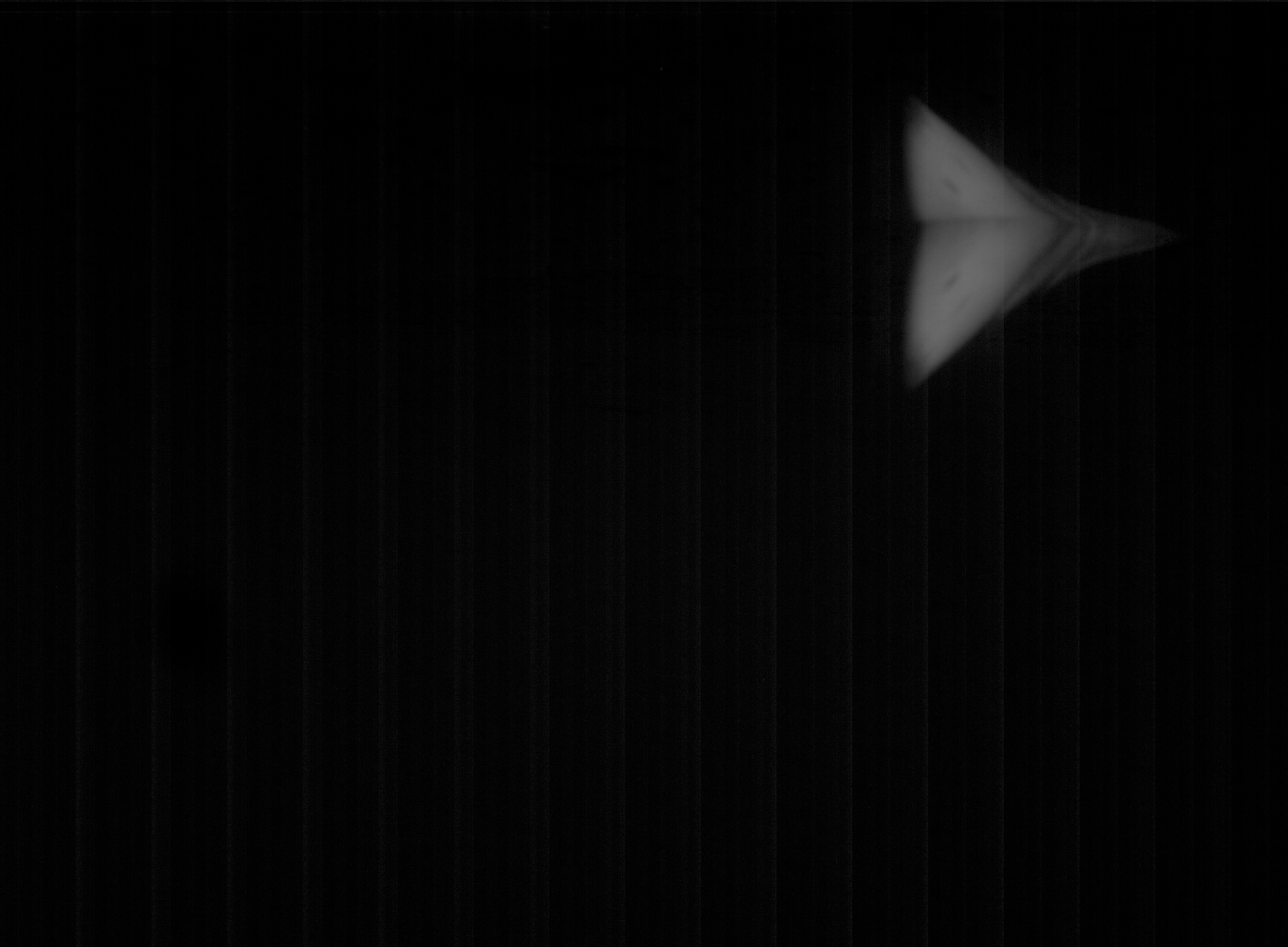

Supplement: S2 File — (ZIP) [file pone.0178461.s004.zip › underwater image data/3.tif]

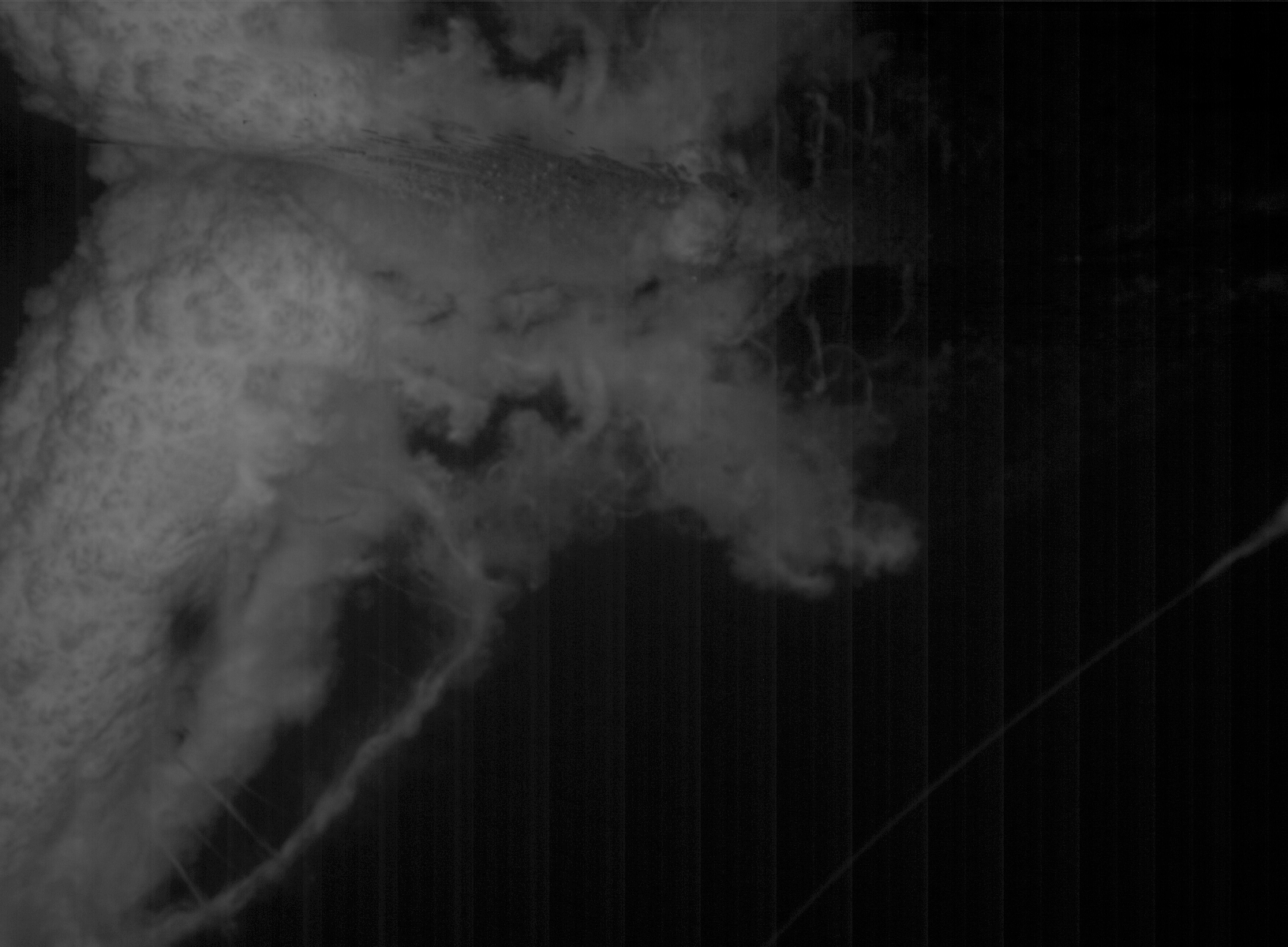

Supplement: S2 File — (ZIP) [file pone.0178461.s004.zip › underwater image data/30.tif]

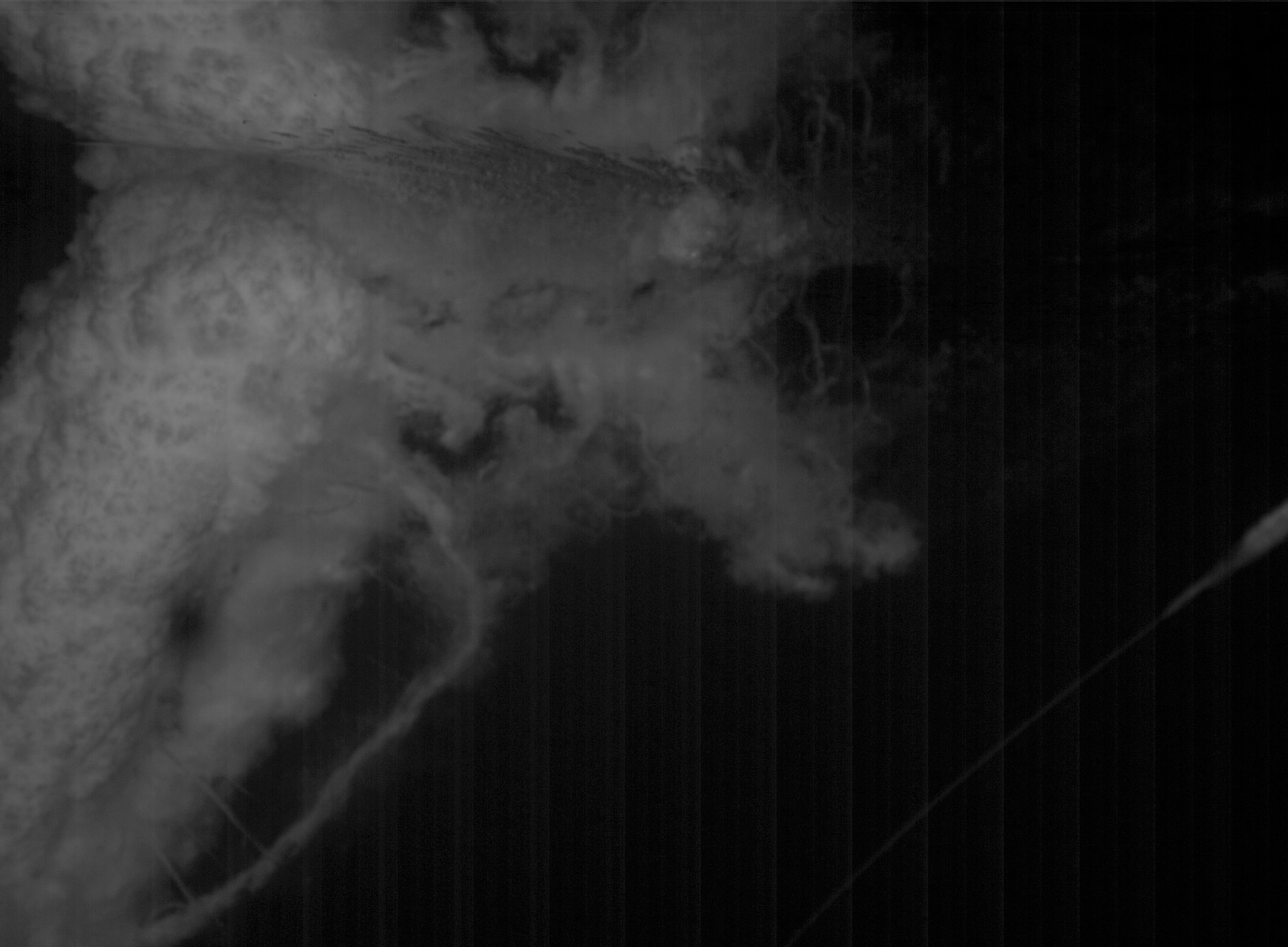

Supplement: S2 File — (ZIP) [file pone.0178461.s004.zip › underwater image data/31.tif]

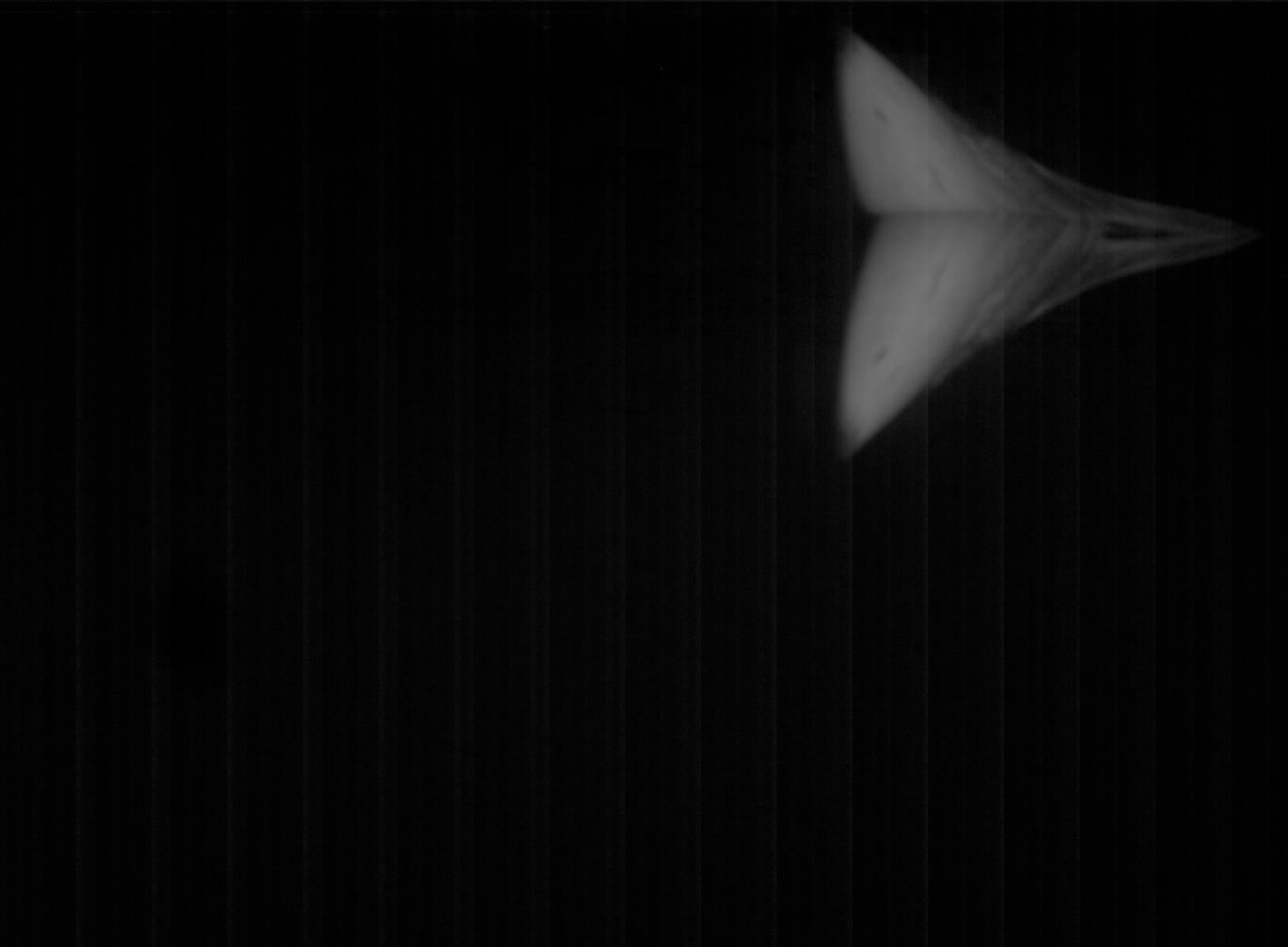

Supplement: S2 File — (ZIP) [file pone.0178461.s004.zip › underwater image data/4.tif]

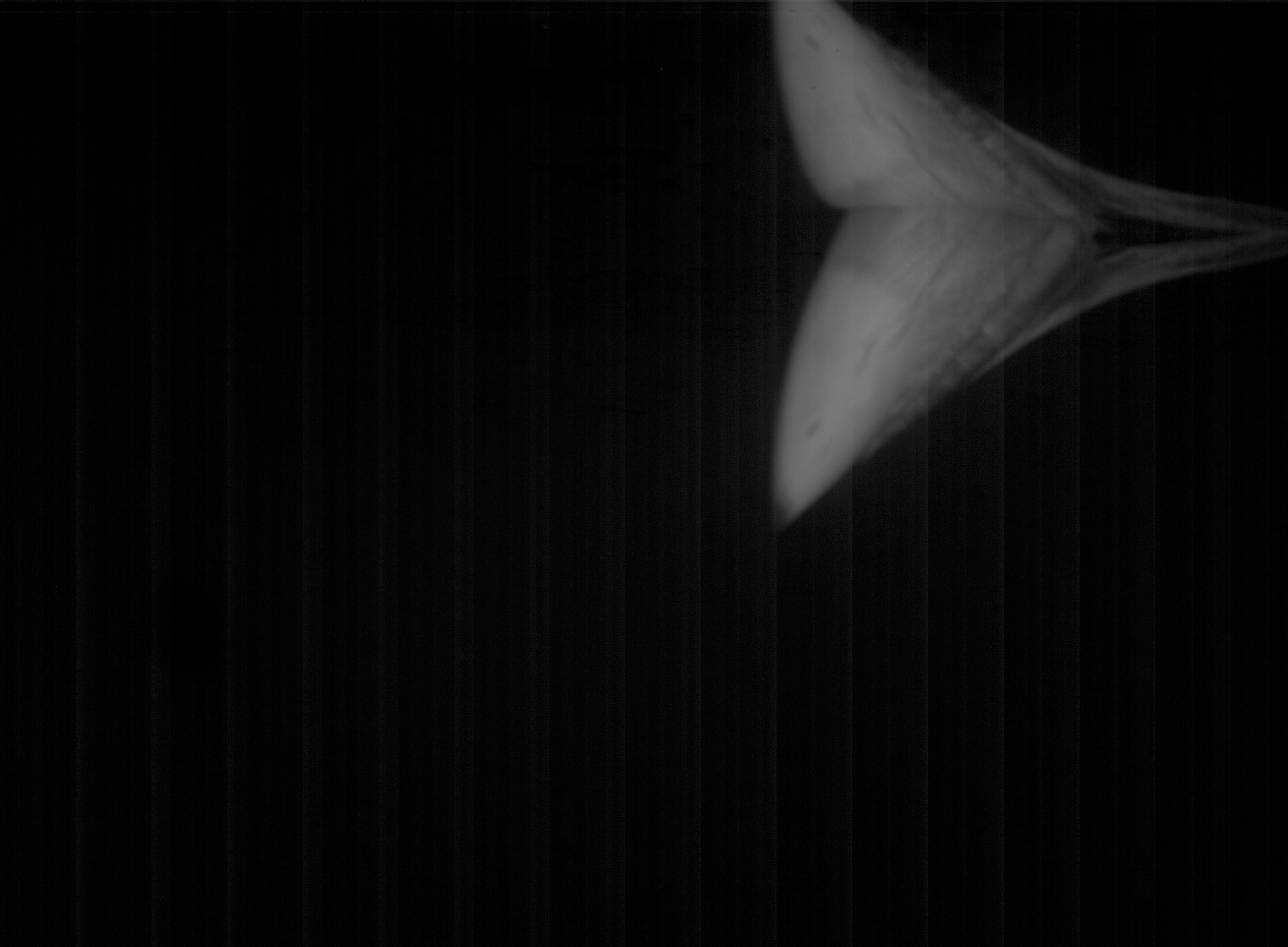

Supplement: S2 File — (ZIP) [file pone.0178461.s004.zip › underwater image data/5.tif]

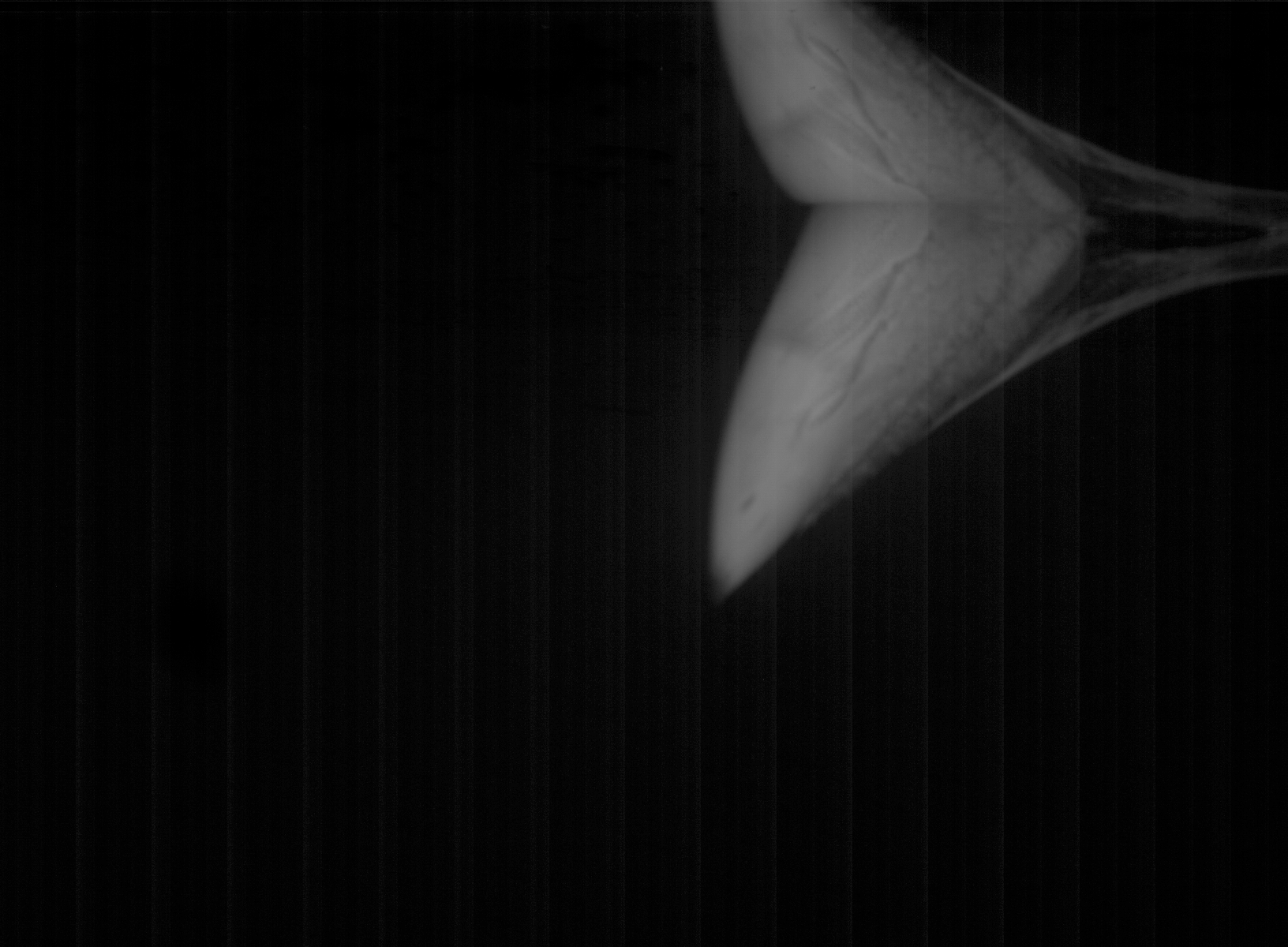

Supplement: S2 File — (ZIP) [file pone.0178461.s004.zip › underwater image data/6.tif]

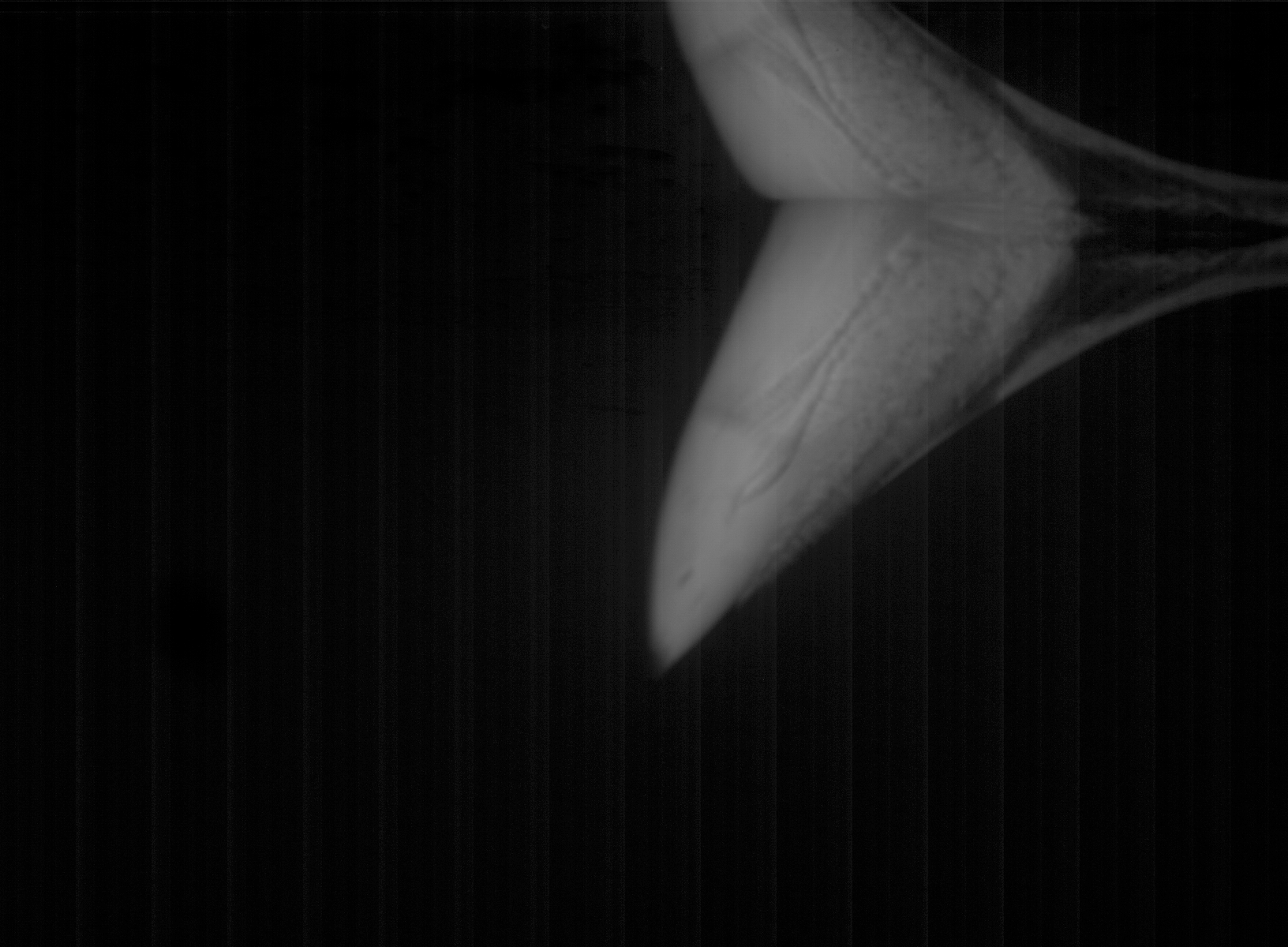

Supplement: S2 File — (ZIP) [file pone.0178461.s004.zip › underwater image data/7.tif]

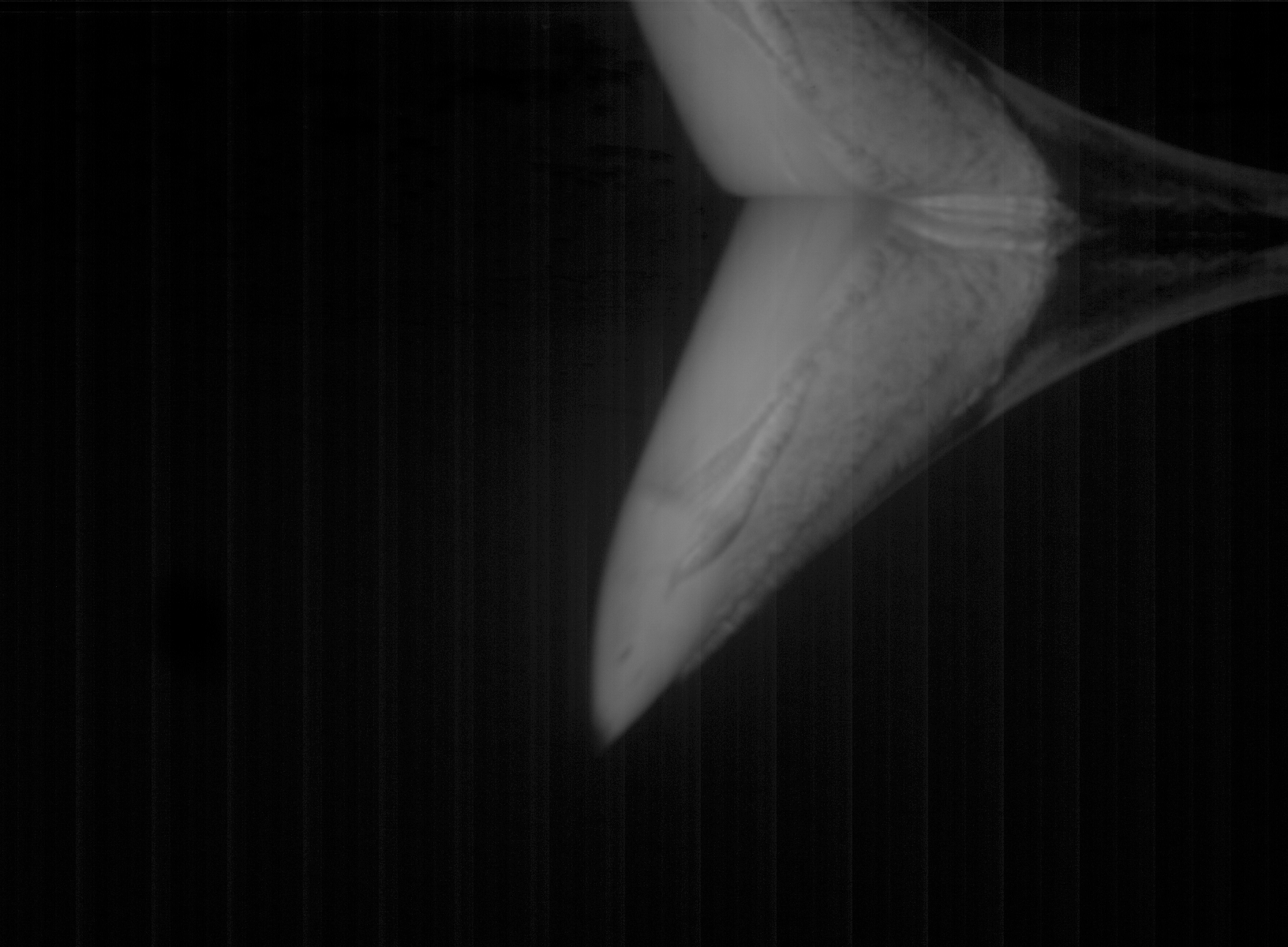

Supplement: S2 File — (ZIP) [file pone.0178461.s004.zip › underwater image data/8.tif]

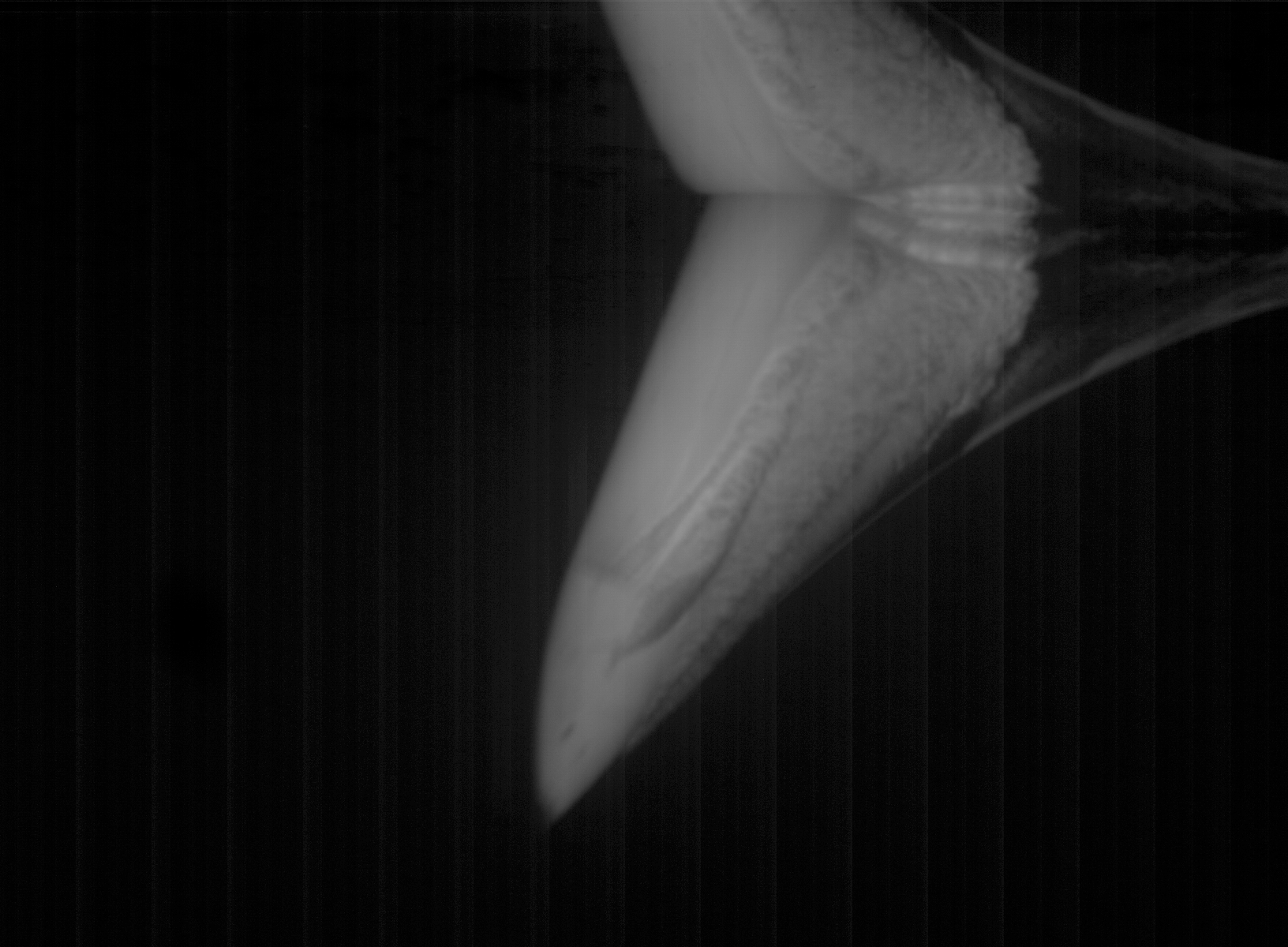

Supplement: S2 File — (ZIP) [file pone.0178461.s004.zip › underwater image data/9.tif]

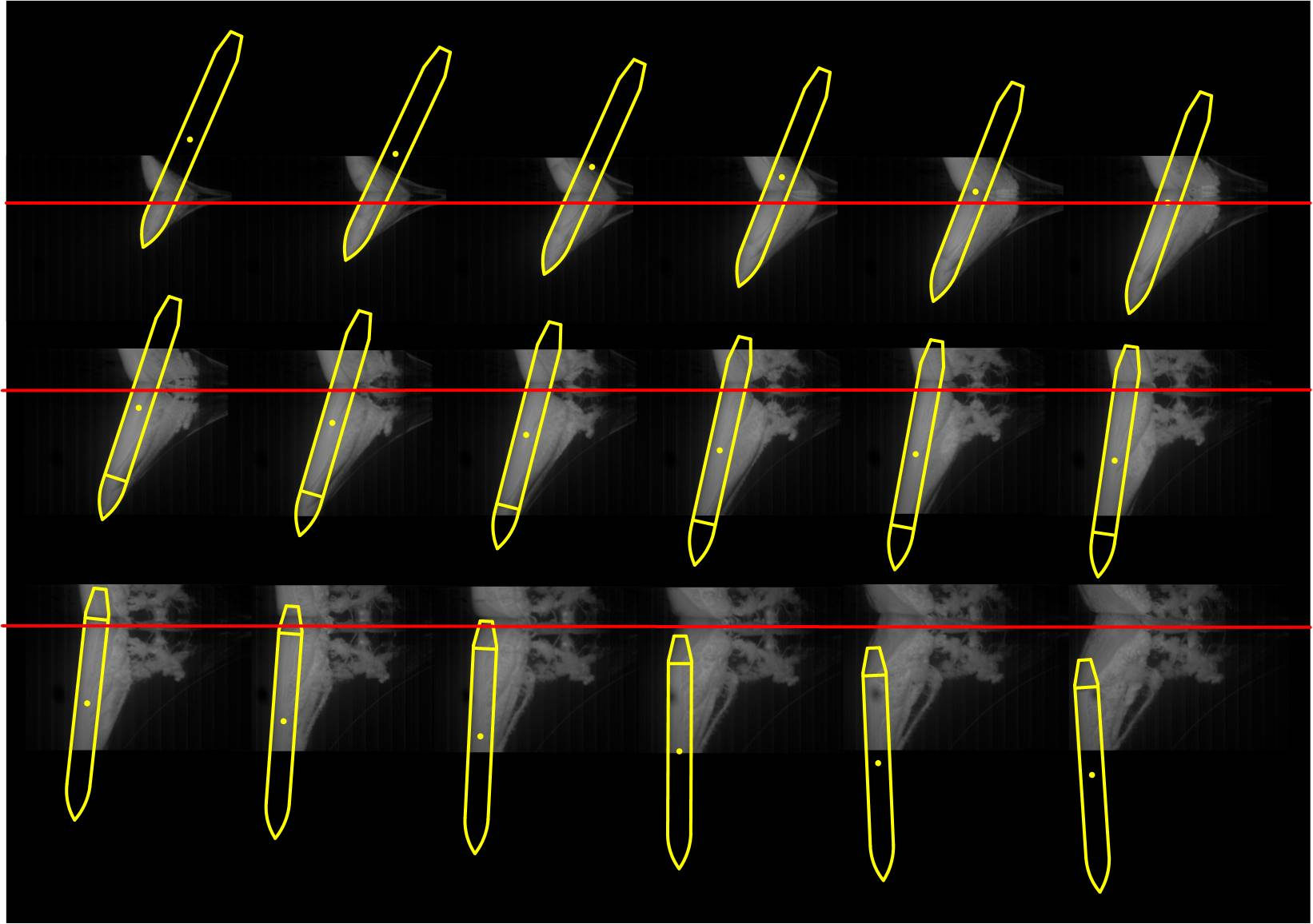

Supplement: S2 File — (ZIP) [file pone.0178461.s004.zip › underwater image data/Complete process_1.tif]

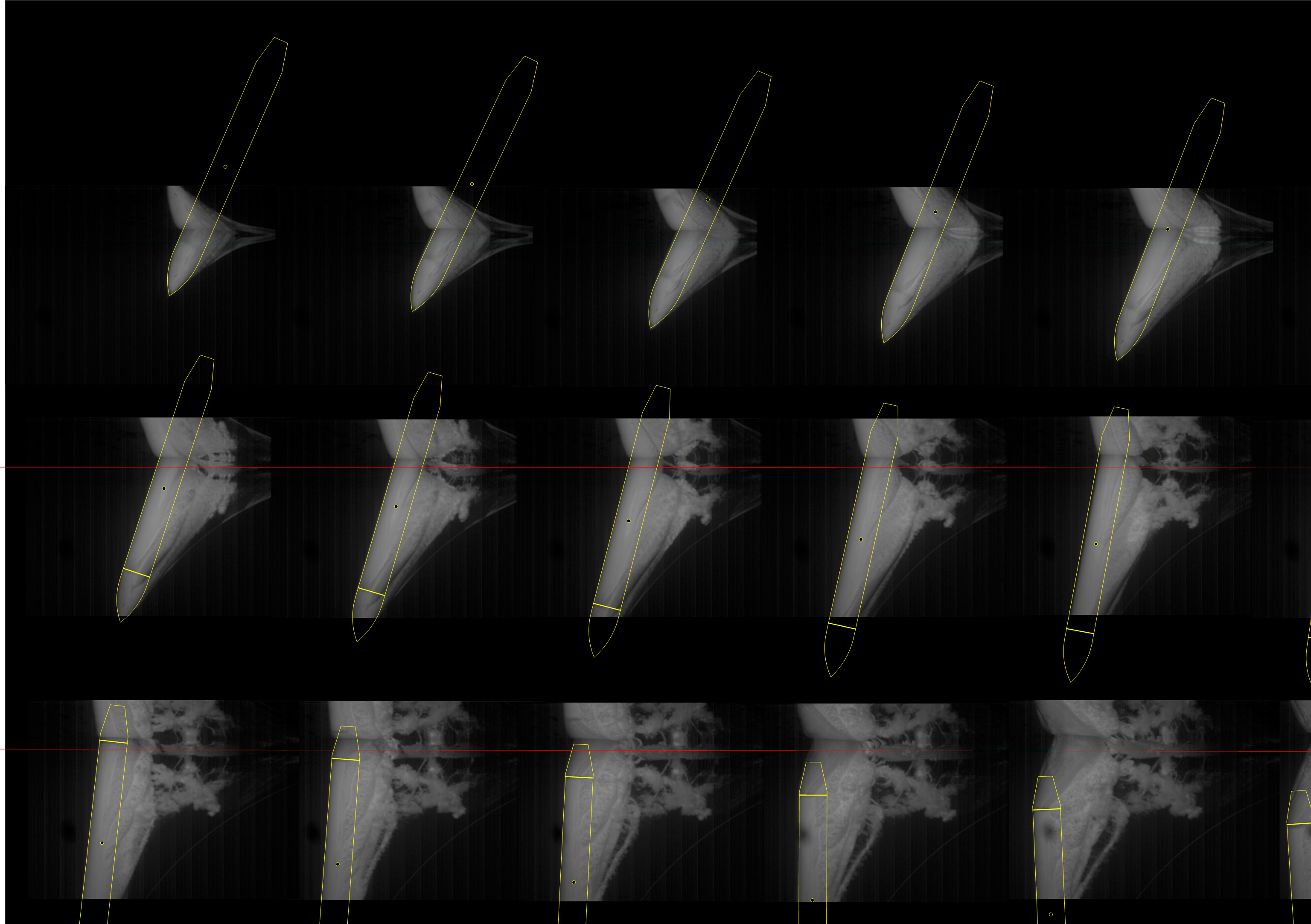

Supplement: S2 File — (ZIP) [file pone.0178461.s004.zip › underwater image data/Complete process_2.tif]

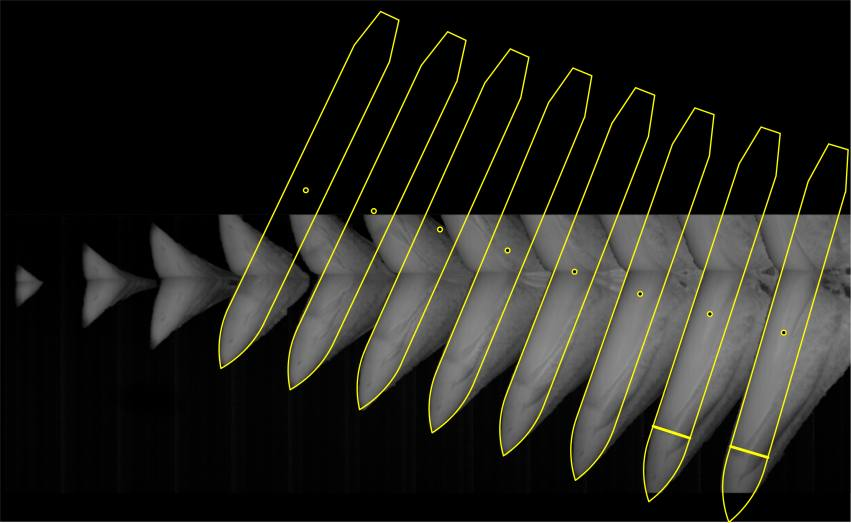

Supplement: S2 File — (ZIP) [file pone.0178461.s004.zip › underwater image data/Complete process_3.tif]

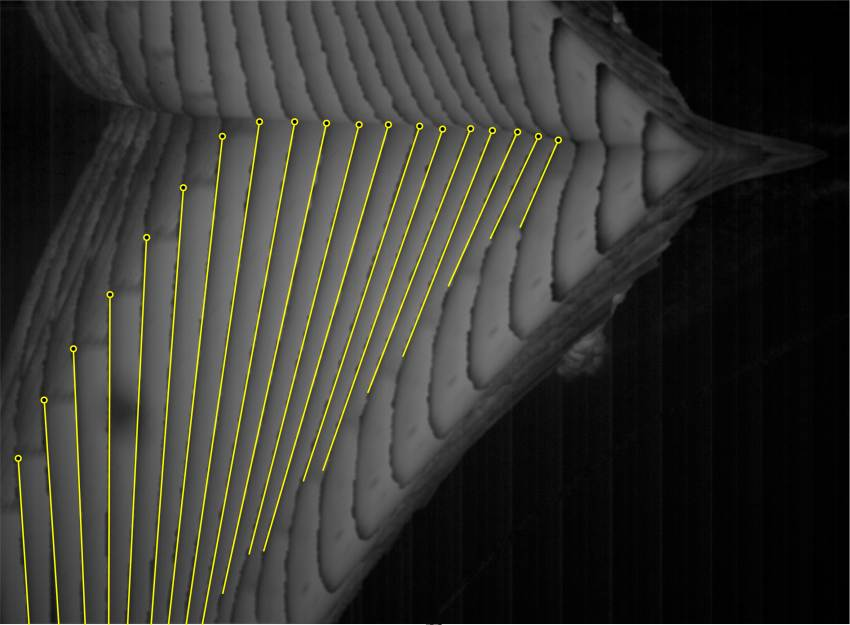

Supplement: S2 File — (ZIP) [file pone.0178461.s004.zip › underwater image data/the change of inclination angle.tif]
